# Introducing Borsantrazole: A Trifunctional Boron‐Based Pyrazole That Extends the Lifespan of Amyotrophic Lateral Sclerosis Mice

**DOI:** 10.1002/advs.76432

**Published:** 2026-07-26

**Authors:** Nitesh Sanghai, Rhonda Kelley, Ying Lao, M. Immanuel Reyes Madlangsakay, Prasanta Paul, M. Alejandra Llanes‐Cuesta, Jun‐Feng Wang, René P. Zahedi, Jiming Kong, Geoffrey K. Tranmer

**Affiliations:** ^1^ College of Pharmacy University of Manitoba Winnipeg Manitoba Canada; ^2^ Central Animal Care University of Manitoba Winnipeg Manitoba Canada; ^3^ Manitoba Centre for Proteomics and Systems Biology University of Manitoba Winnipeg Manitoba Canada; ^4^ Department of Internal Medicine University of Manitoba Winnipeg Manitoba Canada; ^5^ Department of Pharmacology and Therapeutics University of Manitoba Winnipeg Manitoba Canada; ^6^ Department of Biochemistry and Medical Genetics University of Manitoba Winnipeg Manitoba Canada; ^7^ Department of Human Anatomy and Cell Science University of Manitoba Winnipeg Manitoba Canada

**Keywords:** amyotrophic lateral sclerosis, borsantrazole (BSZ), neurodegeneration, proteomics, phosphoproteomics, superoxide dismutase 1, SOD1, SOD1‐G37R ALS animal model, oxidative stress

## Abstract

**Background:**

Amyotrophic lateral sclerosis (ALS) is considered a highly complex, heterogeneous, fatal disease with a high unmet medical need that affects multiple pathophysiological pathways and has no known singular cause. Oxidative stress, however, is implicated as a central player in the progression of ALS and other neurodegenerative diseases. To date, only two FDA‐approved drugs, Edaravone, an antioxidant, and Riluzole, an antiglutamatergic, have been widely used clinically, albeit with modest effects on the clinical course of ALS disease progression. Additionally, preclinical studies of both drugs in ALS mouse models have not shown any significant survival benefit, although some abatement in disease progression is observed and translated from preclinical animal models to clinical human studies.

**Methods:**

Using a trifunctional boron‐based drug design strategy, we have synthesized a pyrazole small molecule called Borsantrazole (BSZ) and evaluated BSZ in cell‐based experiments, acute and chronic toxicity models, and the SOD1‐G37R ALS mouse model. We have also evaluated untargeted global proteomic and phosphoproteomic changes induced by BSZ.

**Results:**

Borsantrazole (a small molecule that can selectively target oxidative stress) with favorable CNS drug like properties (low ER values of 0.9 and 1.0 at 1 and 10 µm, respectively, suggesting lower Pgp efflux liability and the LogD at pH 7.4 was within the range of 1.2 to 3.1, suggesting BSZ is lipophilic at pH 7.4.) shows no signs of treatment associated toxicity (acute or chronic (120 days)), significantly increases survival (whole animals, 15.1 days (*p* = 0.0326); males, 16.5 days; females, 13.7 days), rescued weight loss (27.1% control, 18.3% BSZ, *p* < 0.0001), delays symptom onset (whole animals, 24.9 days (*p* = 0.0011); males, 23.3 days; females, 26.4 days), delays disease onset (whole animals, 25.5 days (*p* = 0.0001); males, 21.1 days; females, 29.8 days) and affects global proteomic changes in the SOD1‐G37R mouse model of ALS. In total, 51 proteins were found to be significantly differentially expressed (*p* < 0.05), including Ca3, Gan, Cplx2, Lrp4, Sqstm1, and 29 phosphorylation sites were differentially expressed and considered statistically significant, including T317 and T72 for neurofilaments light and heavy chain, respectively.

**Conclusions:**

Herein, we report that BSZ has demonstrated a favorable safety profile and compelling *proof‐of‐concept* efficacy in a ALS mouse model and has the potential to become a disease‐modifying ALS therapeutic following further clinical development. Within a broader perspective of treatments for neurodegenerative diseases, BSZ offers a new paradigm for trifunctional small molecule targeting of oxidative stress that can mitigate neuronal deterioration and serve as a potential treatment.

## Graphical abstract

Herein we report a boron‐based pyrazole, (Borsantrazole ‐ a small molecule that selectively targets oxidative stress) that significantly increases survival, reduces weight loss, delays disease onset, and affects global protein changes in the SOD1‐G37R mouse model of ALS. Borsantrazole has demonstrated superior efficacy to currently approved therapies in comparison to historical ALS mouse model results and has the potential to become a ‘best‐in‐class’ ALS therapeutic.

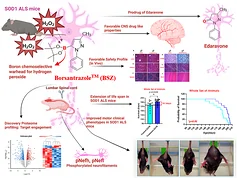

**Abbreviations**
ACN: acetonitrile
ALS: Amyotrophic lateral sclerosis
BSZ: Borsantrazole
CACS: Central Animal Care Services
Cplx2: Complexin 2
DDA: data‐dependent acquisition mode
DEE: diethyl ether
DMEM: Dulbecco's modified eagle medium
DOM: directed Ortho metalation
DTT: dithiothreitol
EDR: Edaravone
fALS: familial ALS
Gan/KLHL16: Gigaxonin
H2O2: hydrogen peroxide
HO•: hydroxyl radical
KNN: K‐nearest neighbor
KNN: K‐nearest neighbor
LMN: lower motor neurons
Lrp4: Low‐density lipoprotein receptor‐related protein 4
n‐BuLi: N‐butyl lithium
Nefh: Neurofilament Heavy Chain
Nefl: Neurofilament light chain
NSC: neuroblastoma‐spinal cord
NSC‐34: neuroblastoma‐spinal cord hybrid NSC‐34
OPB: 2‐oxo‐3‐(phenylhydrazono)butanoic acid
PCNC: primary cortical neuronal cell cultures
PCR: Polymerase Chain Reaction
Pgk1: Phosphoglycerate kinase 1
PINBOP: isopropoxy 4,4,5,5‐tetramethyl‐1,3,2‐dioxaborolane
RLZ: Riluzole
ROS: reactive oxygen species
sALS: sporadic ALS
Snx13: sorting nexin‐13
SOD1: superoxide dismutase 1
Sqstm1/p62: sequestosome 1
TFA: trifluoracetic acid
UMN: upper motor neurons
WHO: World Health Organization

## 1 Background/Introduction

Amyotrophic lateral sclerosis (ALS) is a relentlessly progressive motor neurodegenerative disease that results from the death of both upper motor neurons (UMN) and lower motor neurons (LMN) in the brain and spinal cord. ALS is not a single disease, as it is a highly complex, clinically heterogeneous, pathologically multifactorial neurodegenerative disease. ALS is a rare neurodegenerative disease with an ultimately fatal clinical endpoint [1, 2]. It is proposed that there will be a huge surge in ALS cases from 22 2801 in 2015 to 3 76 674 in 2040, representing an epidemiologic increase of 69% [3]. Since the discovery of ALS in 1869 by Jean‐Martin Charcot [1] and the landmark discovery of the SOD1 mutation by Rosen et al. in 1993 as a causal factor for the progression of human familial ALS (fALS) [4], only two FDA‐approved drugs, Edaravone (EDR), an antioxidant, and Riluzole (RLZ), an antiglutamatergic, have been widely available and clinically used, although with minimal effects on disease course [5, 6]. RLZ was approved for ALS in 1995, based on increased survival in two human clinical trial studies [7, 8], resulting in a modest extension of life of only 2–3 months [7]. However, RLZ has not shown any extension of survival in pre‐clinical assessment in mouse models of ALS, including transgenic hSOD1‐G93A [9, 10, 11, 12, 13, 14], low copy number hSOD1‐G37R line‐29 [15], FUS, and TDP‐43 [9, 16]. Another significant milestone was the approval of EDR by the FDA in 2017, which was tested pre‐clinically in a hSOD1‐G93A mouse model and demonstrated the significant neuroprotective pre‐clinical benefits of slowing motor decline, reducing neuronal oxidative stress and motor neuron loss, and mSOD1 deposition in the spinal cord, although no significant survival benefit was found. The likely mechanism of action of EDR is thought to be due to the reduction of free radicals, including lipid peroxides and hydroxyl radicals, however, the cellular and molecular mechanism of its action is unidentified and still under investigation and emerging [17, 18, 19]. Edaravone was first recognized in the 1980s as a small organic molecule free radical scavenger when it was developed in Japan for the treatment of acute stroke. After this landmark discovery, EDR became a versatile and potent antioxidant that has been found to modify multiple disease pathologies in both in vitro and in vivo models [20, 21, 22, 23, 24, 25, 26, 27, 28, 29, 30]. Both these drugs (EDR & RLZ) are currently the only first‐line “Hope” for the treatment of ALS, although with only modest disease‐modifying effects, as exemplified by the failure of these drugs to improve survival in ALS mouse models.

Until now, 185 SOD1 mutations have been discovered and are known to account for 20% of familial ALS (fALS) and 2%–7% of sporadic ALS (sALS) cases [31]. Mounting evidence has shown that both fALS and sALS are clinically and pathologically indistinguishable, and the clinical disease symptoms and progression of the disease are the same [32, 33]. Further, familial cases remain unrecognized with low disease penetrance in the ancestors of sporadic cases [34]. Evidence from both the preclinical transgenic mouse models of ALS and clinical studies from postmortem tissues of human ALS patients demonstrate that the oxidatively modified misfolding of human wtSOD1 [35, 36, 37, 38, 39, 40, 41, 42, 43, 44] and human mutSOD1 [45, 46, 47, 48, 49, 50, 51] share a common pathogenic pathway leading to the death of motor neurons. Therefore, oxidatively modified SOD1, a result of pathological cellular oxidative stress, acts as a common neurotoxic determinant in familial and sporadic forms of ALS [52, 53, 54, 55, 56]. Several studies from the past three decades deciphered the role of H_2_O_2_ in changing the thiol status of both wtSOD1 and mutSOD1, leading to the toxic gain of function implicated in the pathophysiology of ALS [46, 52, 56, 57, 58, 59, 60, 61]. Further, studies have shown that Cysteine 111 (Cys^111^) serves as a hot spot for oxidative modification under elevated concentrations of neurotoxic H_2_O_2_ [62, 63, 64, 65, 66, 67, 68, 69]. The acquired toxicity(ies) of both mutant and wild‐type human SOD1 initiate a plethora of aberrant oxidative redox cellular pathways in neuronal and non‐neuronal cells, resulting in progressive fatal paralysis due to degeneration of motor neurons [70]. This accumulating evidence from different studies is further supported by the presence of oxidative stress biomarkers during the disease progression of ALS, both in transgenic murine models of ALS [71, 72, 73, 74, 75, 76] and in patients with sporadic and familial ALS [71, 77, 78, 79, 80, 81, 82, 83], further confirming the role of free radical‐mediated oxidative damage of biomolecules and etiopathogenesis of global ALS. Emerging evidence from the findings mentioned above has corroborated our conjecture that oxidative stress caused by pathological H_2_O_2_ is a key hallmark in the pathology of ALS, and therefore, should be considered a principal target for ALS therapeutics. More recently, tofersen (Qalsody), an antisense oligonucleotide (ASO), has received accelerated approval from the FDA for SOD1‐associated ALS (SOD1‐ALS), supported by reductions in the surrogate biomarker neurofilament light chain (NfL) [84]. However, its clinical efficacy in slowing disease progression remains unproven, and concerns regarding its benefit–to–risk profile have emerged in recent trials [85]. Moreover, its reliance on intrathecal administration, due to limited BBB permeability [86], poses challenges for patient compliance compared with small molecules such as EDR and RLZ.

Herein, we present for the first time the novel invention of a chemoselective and H_2_O_2_‐activatable boronate warhead, Borsantrazole (BSZ) that serves as an antioxidant prodrug of EDR, with the demonstrated ability to modulate the propagation of oxidative stress and thereby modify clinical motor phenotypes, in addition to significantly extending survival, delaying disease and symptom onset, and rescuing weight loss in the SOD1‐G37R ALS mouse model. Simultaneously, BSZ treatment was found to modulate the differential expression of discovery‐based known and unknown (ALS‐associated) global proteins and phosphoproteins in the spinal cord of SOD1‐G37R ALS mice. Furthermore, the discovery of BSZ represents an important step forward in finding a potentially improved/disease modifying treatment for a high‐need fatal orphan disease. Overall, our de‐risked early phase drug development strategy was based on chemoselectively targeting the oxidative stress associated with neurodegeneration via boron as a mechanism to deliver a clinically validated ALS therapeutic, EDR, and develop an improved anti‐neurodegenerative agent with added (trifunctional) therapeutic benefit (vide infra).

## 2 Methods/Results

### 2.1 Green, Bio‐Inspired, Catalyst‐Free Method for the Synthesis of Edaravone from its Boron‐Based Prodrug Borsantrazole (BSZ)

Initially, we set out to illustrate the ability of our boron‐containing pyrazole, BSZ, to be converted into EDR, as a means to first validate the prodrug nature of BSZ, while also providing an alternate synthesis route to EDR. Our current transformation of EDR from its prodrug BSZ is based on a green, bio‐inspired, catalyst‐free transformation of BSZ into EDR. Synthesis of EDR involves chemoselective *ipso*‐hydroxylation of boron‐based prodrug BSZ under microwave conditions. Bio‐based green reagents that are biocompatible and non‐toxic, such as boronic acid ester, lactic acid, and hydrogen peroxide (H_2_O_2_) were utilized for the synthesis of EDR. To the best of our knowledge, no literature reports have detailed this green chemistry approach for the synthesis of EDR. Further, we propose that our chemical process mimics a chemoselective H_2_O_2_‐induced oxidative transformation of boron‐based prodrugs within a cellular environment undergoing oxidative stress during neurodegeneration. This concept can be further referred to as selective targeting of neurodegenerative disease within regions of high oxidative stress, as a target‐based approach to mitigating the progression of motor neurodegeneration in ALS, Figure 1.

**FIGURE 1:**
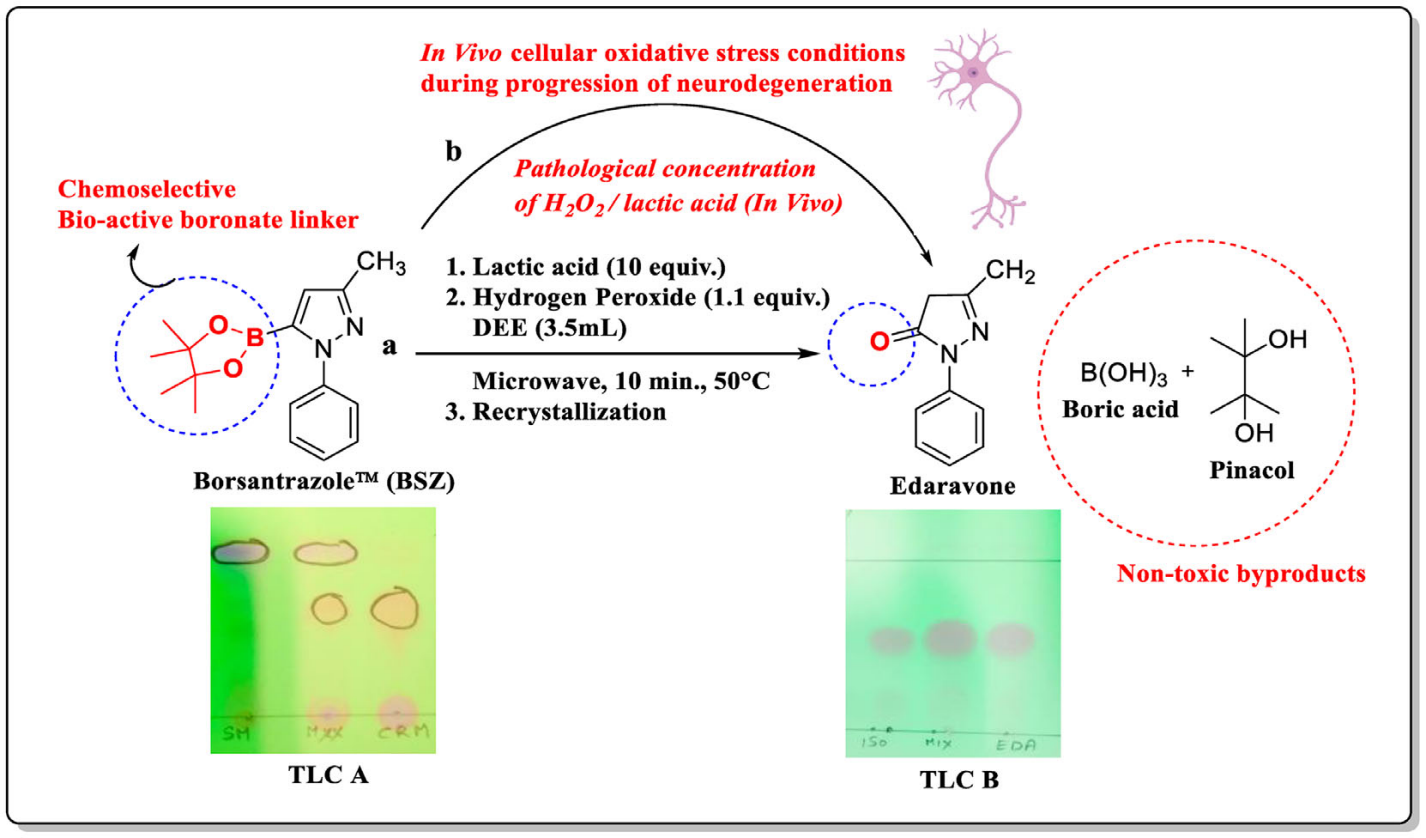
**Schematic illustration of a hydrogen peroxide (H_2_O_2_) triggered boron‐based prodrug approach**. Borsantrazole (BSZ), (a boron‐based prodrug of edaravone (EDR)) synthesized according to the scheme I (see Methods and Materials), is masked with a H_2_O_2_‐sensitive, pinacol boronate prodrug moiety, (a) The BSZ prodrug is oxidatively transformed/deprotected in the presence of H_2_O_2_ and lactic acid under microwave conditions in situ to form EDR, (b) BSZ prodrug a is proposed to be bio‐transformed into EDR (b), in vivo on exposure to pathological concentration of H_2_O_2_, with or without lactate, as associated with the oxidative stress pathology of ALS. 1A‐B shows examples of a chemical reaction between B5‐EDR analogue a and H_2_O_2_ to produce EDR (b) and corresponding TLC plots. TLC A: SM = starting material; Mix = mixture of SM and CRM; CRM = Crude Reaction Mixture; TLC B: ISO = isolated product; Mix = mixture of isolated product and EDR; and EDR = Edaravone.

### 2.2 Putative Mechanism for Biotransformation of Boron‐Based EDR Prodrug BSZ Into Edaravone

Figure 2 highlights the proposed mechanism for the in vivo biotransformation of BSZ into Edaravone following reaction with hydrogen peroxide, or other suitable ROS, and lactate/lactic acid. Under pathological concentrations, BSZ is transformed into EDR in vivo, allowing BSZ to serve as an in vivo prodrug of EDR, while also consuming an equivalent of ROS in the process, providing for added therapeutic benefit over EDR itself. Additionally, the in vivo biotransformation would also generate boric acid as a by‐product, a known neuroprotectant. Overall, the proposed in vivo biotransformation provides the basis for our therapeutic approach, wherein a boron‐based pyrazole reacts with ROS, mitigating oxidative stress (initial therapeutic effect), while simultaneously generating EDR in vivo (i.e. EDR prodrug and secondary therapeutic effect), and producing boric acid as a by‐product (tertiary therapeutic effect). We propose, and as the results below outline, BSZ has the potential to become a disease modifying ALS therapeutic, through our trifunctional boron‐based prodrug approach.

**FIGURE 2:**
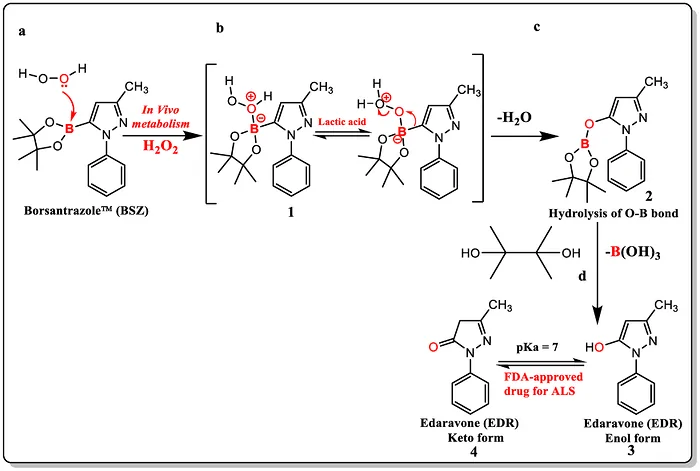
**Schematic proposed mechanism to show how BSZ could act as a redox regulator prodrug under an oxidative cellular environment (in vivo biotransformation with H_2_O_2_) for the generation of EDR**. The mechanism involves (**a)** Nucleophilic attack from the increased concentration of pathological hydrogen peroxide (H_2_O_2_) inside the cells during oxidative stress to the empty *p*‐orbital of the electron‐deficient boron (B) atom to form a tetrahedral boronate complex (**1**). (**b)** The boronate complex **1** undergoes a rearrangement/migration of the C‐B sigma bond onto the adjacent oxygen atom facilitating the loss of water molecule. Lactic acid in step **b** can create an acidic environment facilitating the reaction in the forward direction to form **2**. (**c)** Finally, hydrolysis O–B bond to afford deprotected *ipso*‐hydroxylation product to yield deprotected alcohol **3** and the by‐product of pinacol and boric acid. (**d)** The pKa of EDR is 7.0, and it exists as a solid in keto tautomer form **4**. However, its tautomeric enol form **3** exists as a highly unstable anion in aqueous solution.

### 2.3 Hydrogen Peroxide (H_2_O_2_) Scavenging Ability of Borsantrazole (BSZ)

It was hypothesized that EDR prodrug BSZ could scavenge H_2_O_2_ during its H_2_O_2_‐mediated chemoselective biorthogonal boronate oxidation. We therefore investigated the ability of BSZ to scavenge H_2_O_2_ using Amplex Red assay. The addition of BSZ at the concentration of (500–50 µm) resulted in a markedly significant reduction in the concentration of H_2_O_2_. Around 176 times the concentration of H_2_O_2_ is scavenged by 500 µM of both EDR and BSZ. Further, 352 times and 704 times of H_2_O_2_ is scavenged by both EDR and BSZ at the concentrations of 250 and 125 µm simultaneously. In contrast, EDR and BSZ at the concentration of 50 µM marginally reduced the concentration of H_2_O_2_. However, the H_2_O_2_ scavenging activity at 50 µM of both EDR and BSZ is found to be significant, with a greater mean difference of 24.56 and 13.90, respectively, against positive H_2_O_2_ control. These observations demonstrate that BSZ readily reacts following chemoselective biorthogonal chemistry with H_2_O_2_ to exhibit efficient sequestration of H_2_O_2_ Figure 3.

**FIGURE 3:**
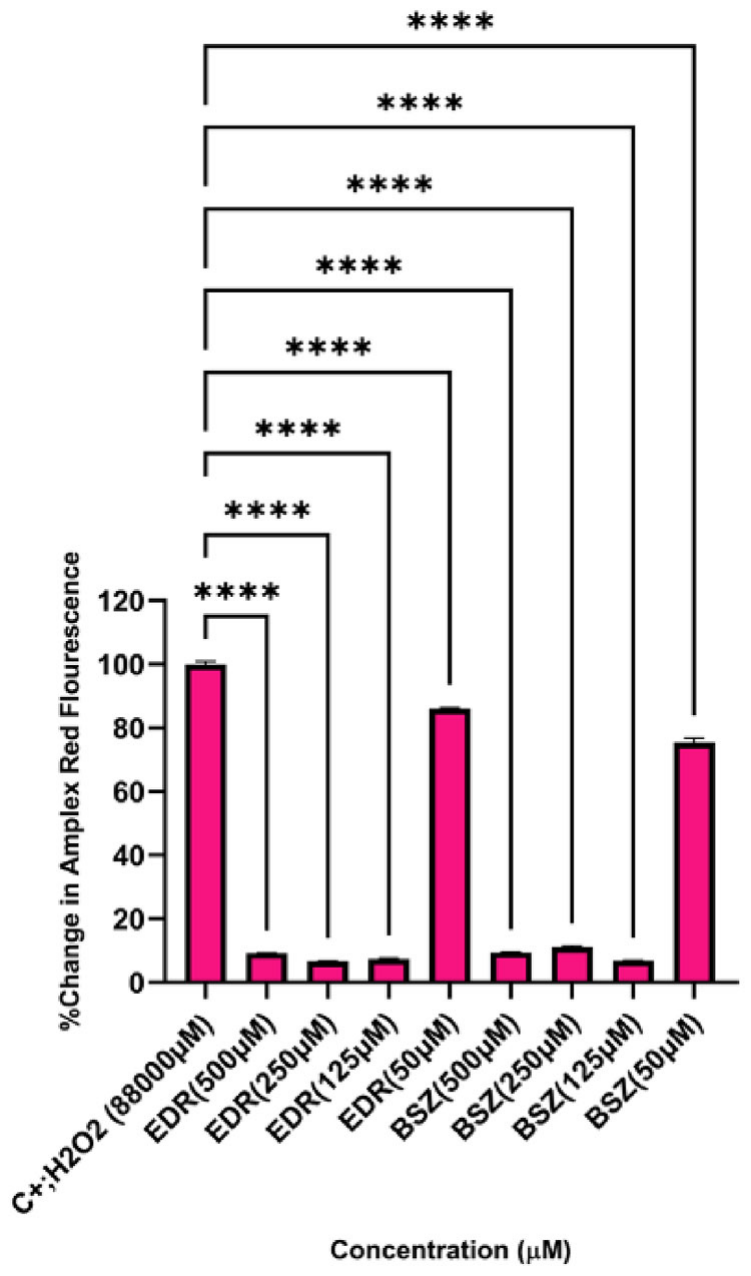
**Evaluation of hydrogen peroxide (H_2_O_2_) scavenging ability of Edaravone (EDR) and Borsantrazole (BSZ) using Amplex Red Assay**. Data are presented as a mean value ± standard error of the mean (error bars); where (a) *n*=18 data points or sample size; data were analyzed using one‐way AVONA followed by Dunnett's multiple comparisons test for statistical analysis, with the significance level set at *p < 0.05*. Asterisks denote statistically significant differences with (****p < 0.0001 versus H_2_O_2_ positive control (C+; H_2_O_2_). Data are representative of three independent experiments, and each measurement or dose was tested six times.

### 2.4 Effect of BSZ on Neurotoxicity/Cell Viability of PCNC

As demonstrated in Figure 4a, BSZ had similar cell viability results as EDR and did not appear to decrease cell viability at concentrations of 1 and 10 µm. Only at much higher concentrations of 25 µm, both EDR and BSZ show diminished cell viability. Thus, based on these results, BSZ does not appear to reduce cell viability to a greater degree than EDR. As shown in Figure 4a, lower concentrations (10 and 1 µm) of BSZ, and EDR showed good cell viability of around 100% compared to controls. Moreover, higher concentrations (25 µm) of EDR and BSZ showed around (70% ± 10%) decrease in cell viability on PCNC compared to control (DMSO). Further, we investigated the effects of EDR prodrug BSZ on the neurotoxicity/cell viability of NSC‐34 cells. As shown in Figure 4b, both EDR and EDR prodrug BSZ increased viability with no cytotoxicity or neurotoxicity compared to control groups. In addition, EDR and BSZ showed around 90%–100% viability in almost every concentration (1–100 µm). Finally, the application of EDR and BSZ from 1 to 100 µm showed little cytotoxicity toward NSC‐34 motor neuronal cells. Based on these cell viability results of prodrug BSZ, we investigated the neuroprotective ability of BSZ against neurotoxic H_2_O_2_ challenge.

**FIGURE 4:**
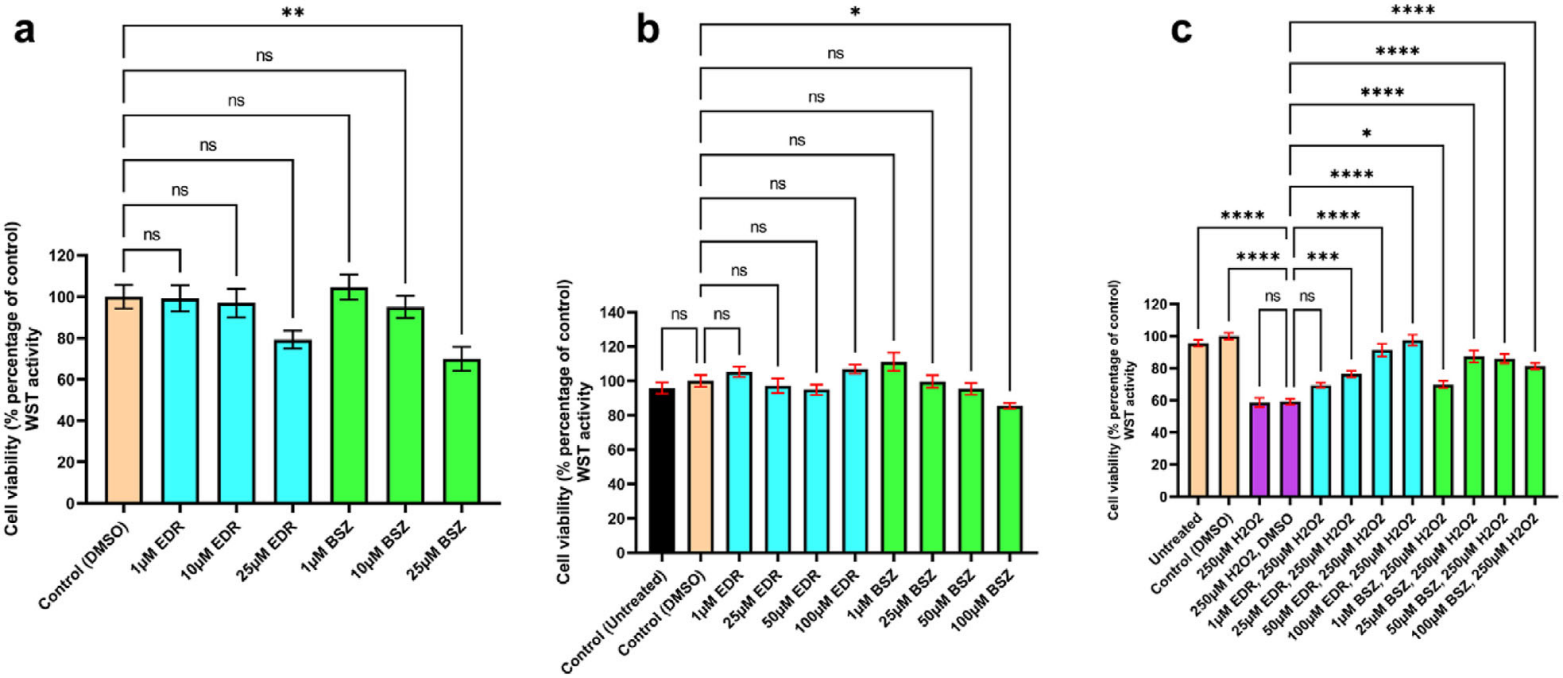
**(a, b, c). Evaluation of neurotoxicity profile of Edaravone (EDR) and Borsantrazole (BSZ) on a. primary cortical neuronal cell cultures (PCNC). b. neuroblastoma‐spinal cord hybrid NSC‐34 cells using WST‐8 analysis (a marker of neurotoxicity). c. Evaluation of neuroprotective profile of EDR and BSZ on neuroblastoma‐spinal cord hybrid NSC‐34 cells against hydrogen peroxide (H_2_O_2_) induced neurotoxicity using WST‐8 analysis (a marker of neurotoxicity)**. Data are presented as a mean value ± standard error of the mean (error bars); where, (a) *n* = 12 (b) *n* = 9 data points or sample size; data were analyzed using one‐way AVONA followed by Dunnett's multiple comparisons test for statistical analysis with the significance level set at *p < 0.05*. Asterisks denote statistically significant differences with (*p<0.05, **p = 0.01, ***p = 0.001 and ****p < 0.0001 and *ns*=non‐significant versus control DMSO). Data are representative of (a) two independent experiments, and each measurement or dose was tested six times, whereas, (b) three independent experiments and each measurement or dose was tested in triplicate.

### 2.5 Neuroprotective Effect of BSZ on NSC‐34 Cells

As shown in Figure 4c, prophylactic treatment of both EDR and BSZ protected NSC‐34 cells from loss of cell viability induced by 250 µm H_2_O_2_ and is neuroprotective against H_2_O_2_‐induced oxidative stress. BSZ showed almost equal viability at a 50 µm dose compared to EDR. Moreover, BSZ showed better neuroprotection in scavenging the neurotoxic effects of H_2_O_2_ compared to EDR at lower doses of 25 µm. This suggests that BSZ is more effective than EDR in scavenging H_2_O_2_ at lower doses. In addition, EDR prodrug BSZ showed better neuroprotection in scavenging the neurotoxic effects of H_2_O_2_ compared to EDR at the lowest dose of 1 µm; suggesting, again, that BSZ is more effective than EDR in scavenging neurotoxic effects of H_2_O_2_ at lower doses. With these results, we can conclude that BSZ, in comparison to EDR, demonstrated higher neuroprotection at lower doses of 1 and 25 µm and equal neuroprotection at 50 µm. At higher doses (i.e., 100 µm), BSZ showed a small decrease in cell viability (10%–15%), meaning EDR may be a better neuroprotectant at higher concentration compared to BSZ, however, in vivo drug concentrations of 100 µm are unlikely to occur.

### 2.6 A Single Dose (Acute Toxicity Assessment) of Borsantrazole (BSZ) and 120 Daily Doses (Chronic Toxicity Assessment) of BSZ for 120 Days With Longitudinal Monitoring Demonstrates a Satisfactory Safety Profile

#### 2.6.1 Morphological Alteration

First, we investigated the safety profile of the newly synthesized prodrug of EDR, BSZ in vivo. As shown in Figure 5, a single dose and 120 doses for 120 days daily with 10 mg/kg body weight of BSZ with longitudinal monitoring for 14 and 120 days, respectively, showed no treatment‐associated deaths or adverse events. Daily general observations showed normal appearance and normal general behavior with well‐conditioned body condition. Furthermore, both the acute and chronic treatment of BSZ did not cause changes to the skin, fur color, eyes, mucous membrane, nor the occurrence of secretions and excretions, and motor activities. IP administration of BSZ (10 mg/kg/day) daily for 120 days, with (a total of 120 IP IJ, and 1200 mg of total dose over 120 days), was seemingly well tolerated by wildtype SOD1 mice. Moreover, BSZ‐treated mice developed no clinical signs of toxicity, including a decrease in mean weight, hunched posture, orbital tightening, piloerection, nor low activity compared to vehicle‐treated (1:20, DMSO: PBS) mice.

**FIGURE 5:**
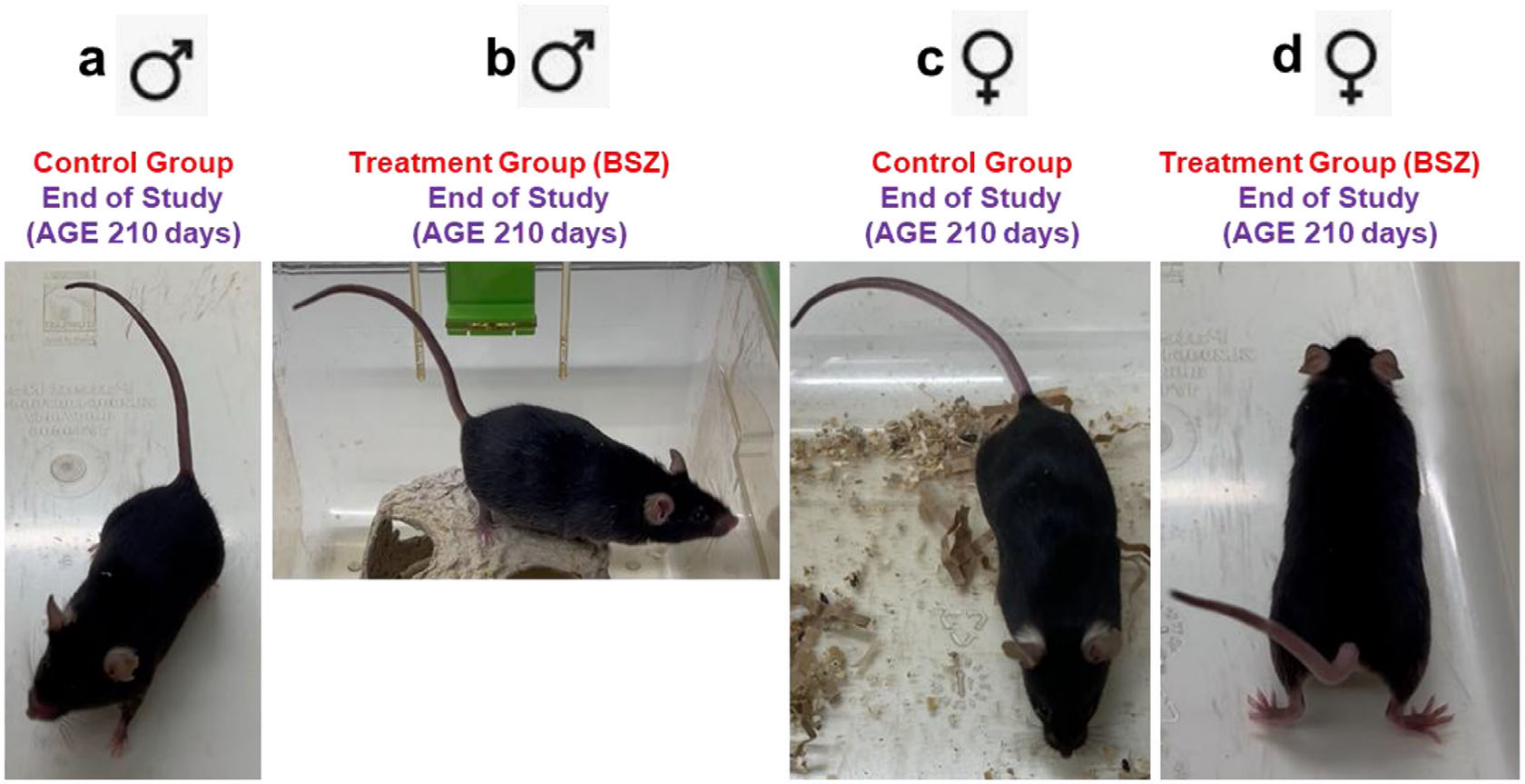
**Chronic BSZ treatment showed no BSZ‐associated clinical signs of toxicity in both sexes**. Borsantrazole (BSZ) was administered intraperitoneally to age‐ and sex‐matched wild‐type SOD1 mice (120 doses, 10 mg/kg/day bodyweight) for 120 days with daily longitudinal monitoring until the age of 210 days, end of study. The subset of control‐treated (1:20, DMSO: PBS) male mice (a) and female mice (c) showed no clinical signs of toxicity in terms of hunched posture, orbital tightening, piloerection, low activity/respiration, and weight loss compared to the BSZ treated male (b) and female mice (d). Both groups exhibited normal posture and activity at the end of the study.

#### 2.6.2 Assessment of Body Weight

Figure 6 highlights our acute and chronic toxicity evaluation plan, with corresponding longitudinal monitoring (14 days and 120 days) and *n* = 6 for each group (3 male and 3 female). As shown in Figure 7, a single dose and 120 daily doses for 120 days with 10 mg/kg bodyweight of BSZ with longitudinal monitoring for 14 and 120 days, respectively, did not induce any abnormal changes in the body weight of the mice. Moreover, there was no significant difference in the changes in body weight between the control group and the treatment groups after 120 daily doses, which further supports the absence of toxicity.

**FIGURE 6:**
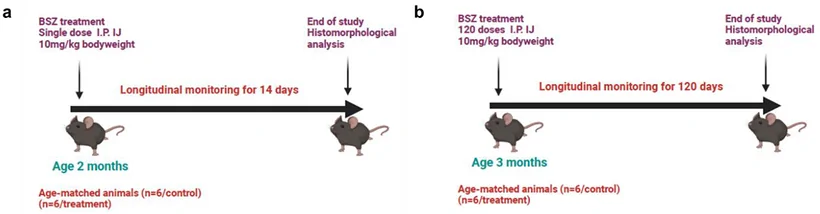
(a) Diagram depicting a single‐dose acute toxicity treatment study plan. (b) Diagram depicting 120 doses of chronic toxicity treatment study plan for 120 daily doses.

**FIGURE 7:**
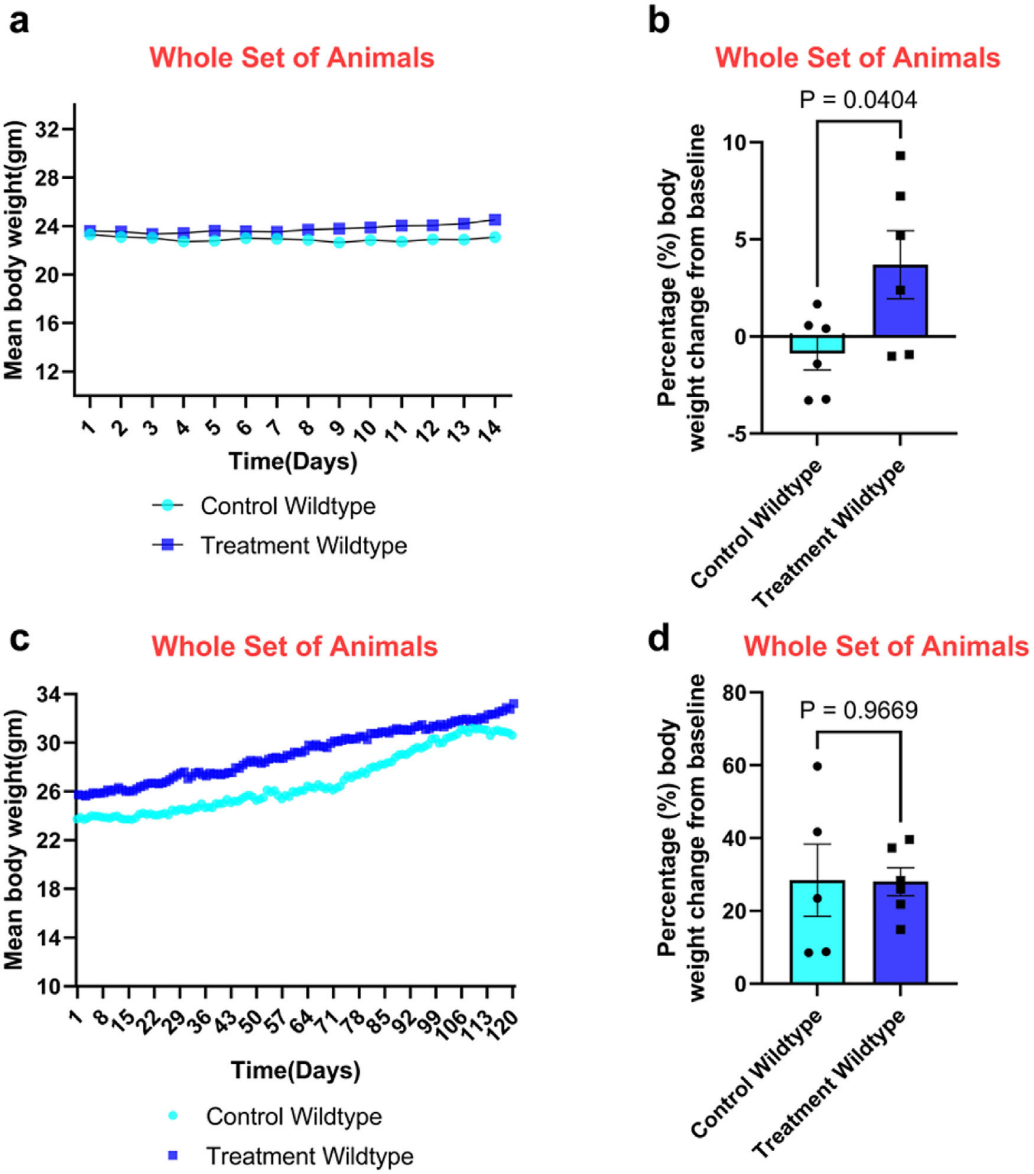
**Results of acute and chronic toxicity assessments on mean body weight over 14 days (acute) and 120 days (chronic) starting at 60 and 90 days of age, respectively. (a) Mean body weight for control and treated groups over a 14‐day period following a single dose. (b)** Two‐tailed (unpaired t‐test) of the percentage mean body weight change from baseline (initial weight) showing that BSZ increases the percentage mean body weight from ‐0.88% for wildtype (WT) SOD1 age‐matched whole set of animals (control/*n *= 6 mice), including (*n* = 3 males and *n* = 3 females) to 3.7% (BSZ/*n* = 6 mice), including (*n* = 3 males and *n* = 3 females).(**c) Mean body weight for control and treated groups over 120 days with 120 daily doses. (d)** Two‐tailed (unpaired *t*‐test) of the percentage mean body weight change from baseline (initial weight) showing that BSZ does not increases the percentage mean body weight 28% for wildtype (WT) SOD1 age‐matched whole set of animals (control/*n* = 5 mice), including (*n* = 2 males and *n* = 3 females) to 28.4% (BSZ/*n* = 6 mice), including (*n* = 3 males and *n* = 3 females). Data are presented as percentage body weight mean difference from baseline (*n* = 12) for the whole set of animals. Where, *n* =  number of animals of the designated genotype. Data were analyzed using a two‐tailed (unpaired t‐test), with a significance set at *p < 0.05*. Here, *p = 0.0404*, *p* = 0.9669 with *p < 0.05 and ns respectively versus control wildtype (WT) SOD1 animals.

In the acute toxicity monitoring (Figure 7a,b), the average initial body weight of the control group (*n* = 6) was 23.31 g, while the treatment group (*n* = 6) averaged 23.61 g. Additionally, in the chronic toxicity monitoring (Figure 7c,d), the average initial body weight for the control group (*n* = 5) was 23.74 g, compared to 25.75 g for the treatment group (*n* = 6). This indicates that the mice in the acute cohorts had very similar average weights at the beginning; however, a notable initial weight difference was observed in the chronic toxicity study, with the treatment group (*n* = 6) being 8.4% heavier than the control group (*n* = 5). It is also noteworthy that after 14 days of monitoring in the acute toxicity cohorts (following a single dose), the mean percentage change in body weight from baseline (initial weight) was 3.7% in the treatment group (*n* = 6). In contrast, the control group (*n* = 6) experienced a decrease of ‐0.88%. These results account for less than a 5% difference between the two groups, and although statistically significant (*p* = 0.04, Figure 7b), the larger variation in the weight gain of the treated mice (two mice >5%, but <10%) could account for this statistical difference between the control and treated groups. However, based on our 120‐day chronic safety study, histological data, and literature reports detailing that weight changes greater than 10% are typically biologically relevant, we believe that the acute safety study was successful. Furthermore, after 120 days of treatment in the chronic toxicity cohorts, the mean percentage change in body weight from baseline (initial weight) was found to be nearly the same (28%) with no significant difference found between the two groups. Our findings from both the acute and chronic toxicity studies show that a lack of biologically relevant percentage mean weight changes from baseline demonstrates that the BSZ group, both in acute and chronic toxicity, did not experience a large reduction in body weight compared to the controls. These results suggest that there is no weight‐based indication of toxicity from BSZ in any group (Figure 7a–d).

#### 2.6.3 Histopathological Findings

As shown in Figures 8 and 9, a single dose and 120 daily doses for 120 days with 10 mg/kg body weight of BSZ with longitudinal monitoring for 14 and 120 days, respectively, exhibited no signs of treatment‐associated hematological toxicity in tissues (Liver, kidney, spleen, lungs, heart, and brain) tissues from both sexes (males/females). H&E staining images of all six major organs as mentioned showed no significant abnormalities difference between the histology of the control and treated (BSZ) group, with no signs of overt degeneration, inflammation, and necrosis in any of the examined tissues.

**FIGURE 8:**
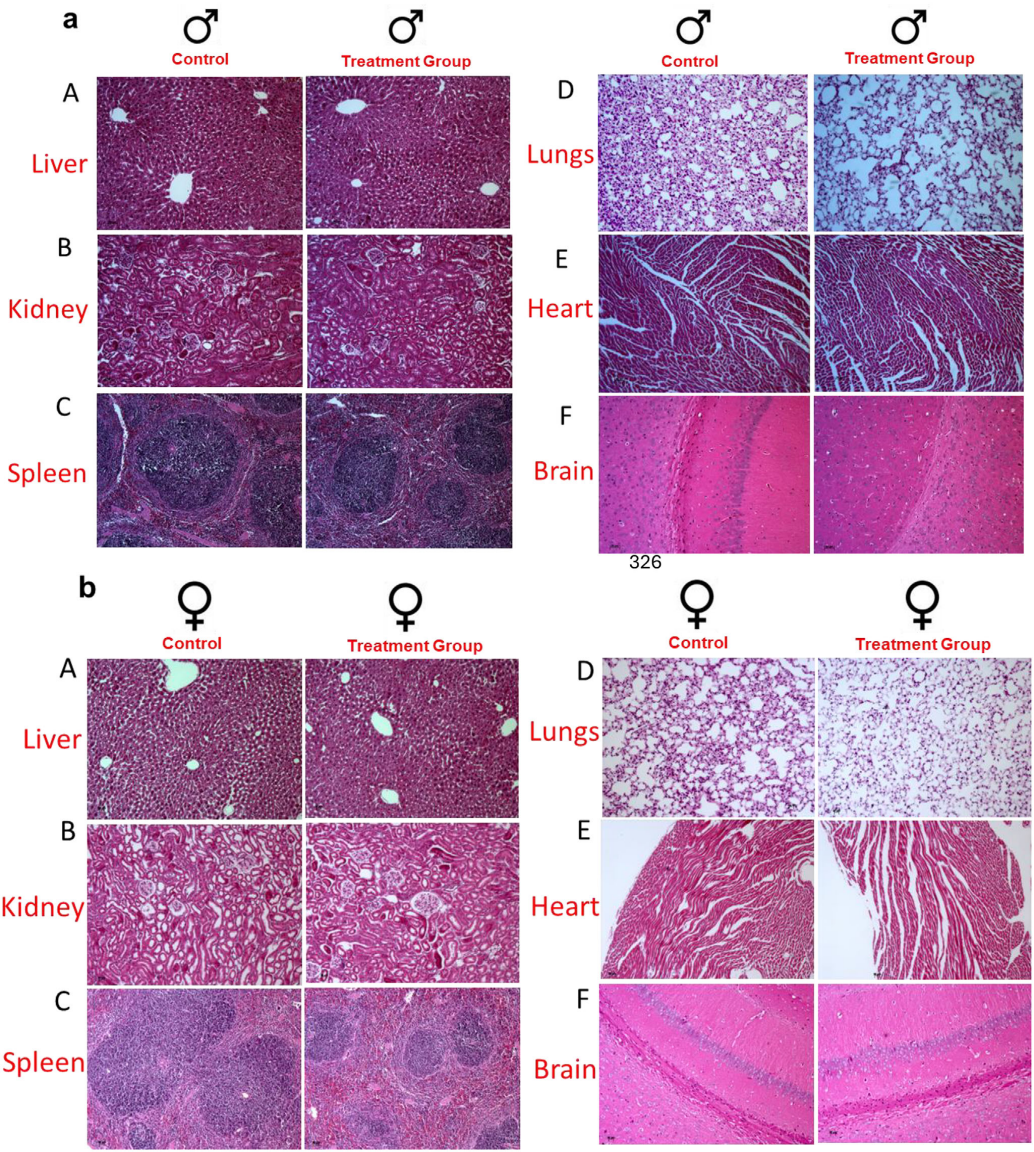
**(a,b). Photomicrographs of Hematoxylin and Eosin (H&E) stained sections of wildtype (WT) SOD1, male and female mice from acute toxicity evaluation**. Beginning at 60 days of age, WT female mice received a single intraperitoneal (IP) injection of BSZ at a dose of 10 mg/kg bodyweight. The mice were longitudinally observed and monitored for 14 days. At the experimental endpoint, animals were euthanized and tissues, including brain, heart, kidney, liver, lung, and spleen, were collected for analysis. In the H&E staining images, the nucleus is blue, and the cytoplasm is red. Collagen fibers show a varying red color. There were six mice (3 male and 3 female) in each group, with original magnification 10× (Scale bar represents 20 𝜇𝑚). All the images were taken with Microscope: Axioskop 2 mot plus microscope using AxioVision software version 4.8 (Carl Zeiss, Inc., Thornwood, NY).

**FIGURE 9:**
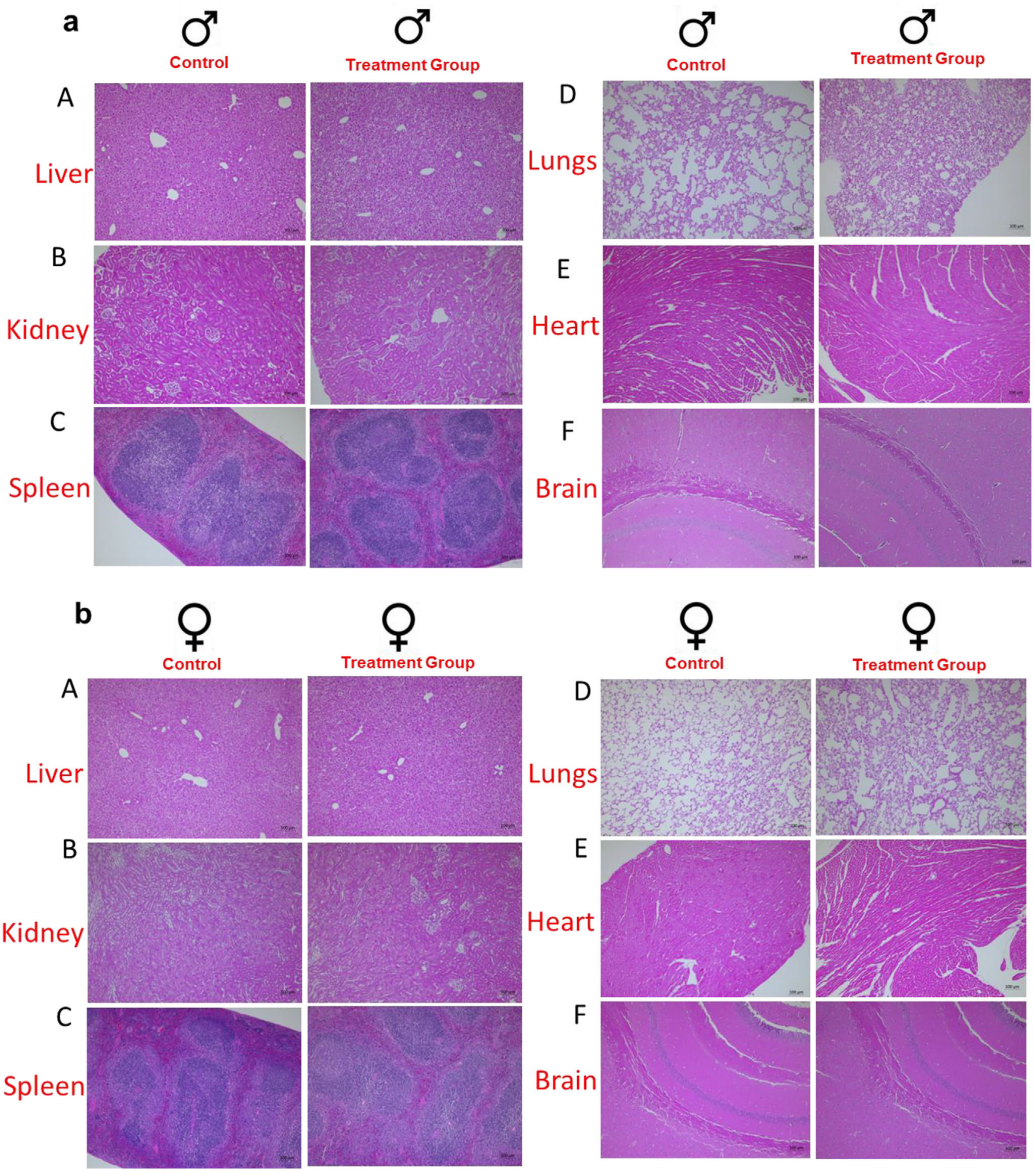
**(a,b). Photomicrographs of Hematoxylin and Eosin (H&E) stained sections of wildtype (WT) SOD1, male and female mice from chronic toxicity evaluation**. At 3 months of age, WT, male, and female mice received 10 mg/bodyweight of BSZ daily for 120 days (a total of 120 doses). The mice were longitudinally observed and monitored for 120 days. At the experimental endpoint, animals were euthanized and tissues, including brain, heart, kidney, liver, lung, and spleen, were collected for analysis. In the H&E staining images, the nucleus is blue, and the cytoplasm is red. Collagen fibres show a varying red color. There were six mice (3 male and 3 female) in each group, with original magnification 10 × (Scale bar represents 100 µm). H&E staining images of such organs showed no difference between the histology of the control and treated (BSZ) group, with no signs of overt degeneration, inflammation, and necrosis in any of the above‐examined tissues. All the images were taken with Microscope: Zeiss Imager M2 microscope using ZEN 3 Pro software with camera AxioCam HRc—color.

### 2.7 Longitudinal Preclinical Study with Borsantrazole (BSZ) Delayed Disease Onset (Age to Peak Body Weight) in the SOD1‐G37R Mouse Model

Disease onset was retrospectively defined as the age at which the mice reached peak body weight before weight began to decline. As shown in Figure 10, daily intraperitoneal injection (IP, IJ) with BSZ starting pre‐symptomatically from the age of 90 days, until the humane endpoint (end‐stage), delayed the disease onset in the age‐matched whole set of animals and both sexes (male/female) of SOD1‐G37R ALS mice to a statistically significant degree, *p* < 0.05. For the whole set of animals, *n* = 12/group, including 6 male and 6 female, both in the vehicle‐treated (control) and BSZ‐treated group, the mean disease onset age differed significantly between the vehicle‐treated (129.7 days) and BSZ‐treated group (155.2 days). Further, the disease onset was significantly delayed by around 25.5 days in the BSZ treatment group compared to the control group. On sexual segregation, we found that BSZ treated males (*n* = 6), and females (*n* = 6) exhibited a delay of onset (21.1 days) and (29.8 days)compared to the control group respectively, with a larger effect size in males (*p* = 0.0008) compared to females (*p* = 0.0151). Sex segregation revealed a sex‐specific therapeutic effect of BSZ in males compared to females, suggesting differences in sex‐specific efficacy of BSZ in delaying the progression of early symptoms in ALS mice. Although disease onset for males had greater statistical significance (*p* = 0.0008 males vs. *p* = 0.0151 females), female mice had a greater protracted latency of onset (21.1 days males vs 29.8 days females) compared to controls. The differences in statistical significance can largely be attributed to a single outlier female in each of the control and treatment groups. The disease onset data are also in tabulated form (Table 1) below.

**FIGURE 10:**
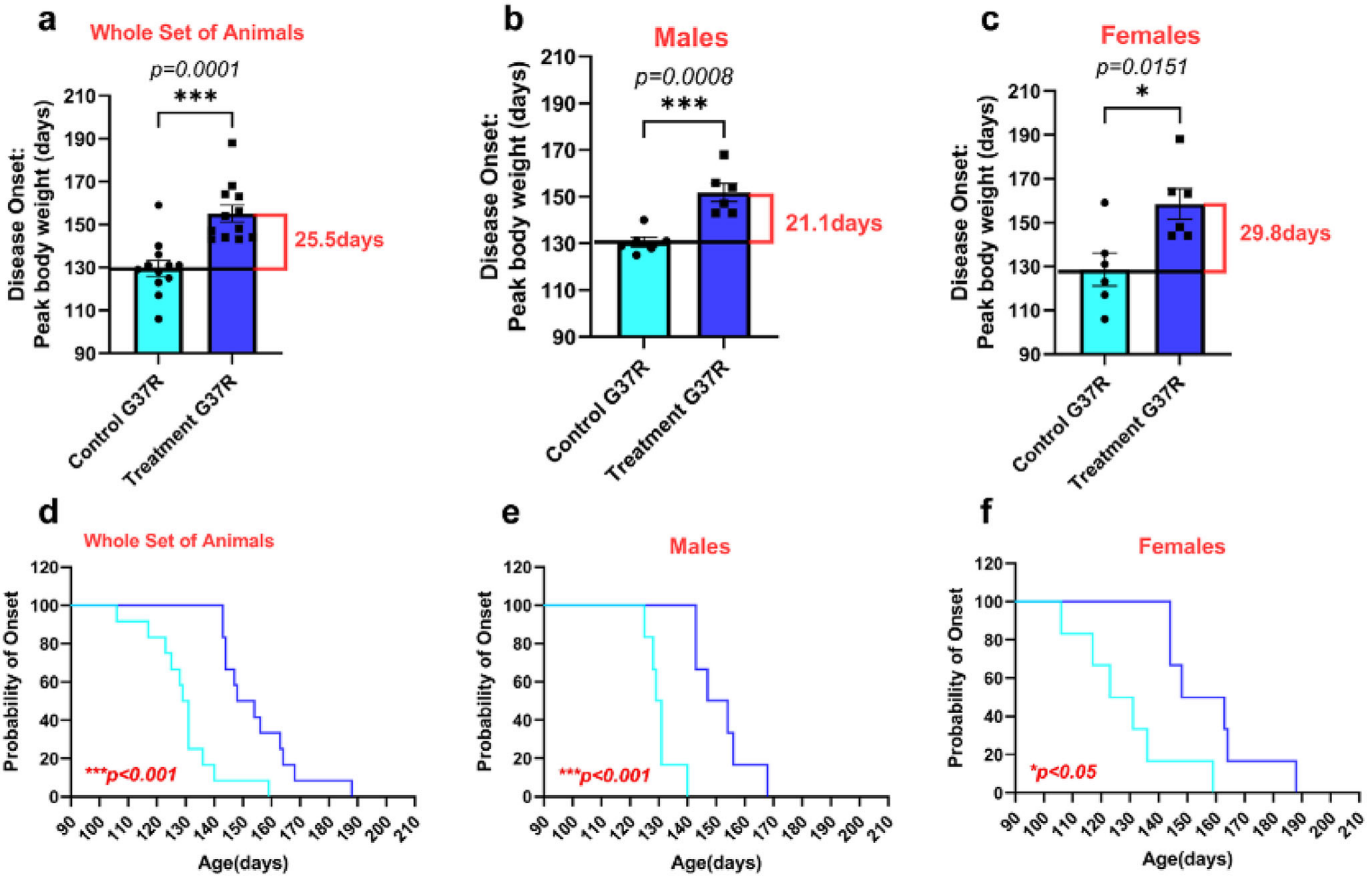
**Borsantrazole (BSZ) delays the disease onset (based on age to peak body weight before weight begins to decline). (a)** Two‐tailed (unpaired *t‐test*) of the mean onset age showing that BSZ delays the mean onset age from 129.7 days for G37R age‐matched whole set of animals (control/*n* = 12 mice), including (*n* = 6 males and *n* = 6 females) to 155.2 days (BSZ/*n* = 12 mice), including (*n* = 6 males and *n* = 6 females); (**b)** from 130.7 days for age‐matched G37R males (control/*n* = 6 mice), to 151.8 days for age‐matched G37R males (BSZ/*n* = 6 mice); (**c)** from 128.7 days for age‐matched G37R females (control/*n* = 6 females), to 158.5 days for age‐matched G37R females (BSZ/*n* = 6 females). Data are presented as mean ± SEM (*n* = 12) for the whole set of animals and *n* = 6 for males and females. *n* =  number of animals of the designated genotype. Data were analyzed using a two‐tailed (unpaired *t‐test*), with a significance set at *p<0.05*. Here, *p=0.0001, p=0.0008 and p=0.015*, with ***p<0.001, ***p<0.001 and *p<0.05 versus control G37R. (**d)** Kaplan–Meier curves of the probability of disease onset showing that BSZ delays the median onset from 130 days for age‐matched G37R whole set of animals (control/*n* = 12 mice), including (*n* = 6male and *n* = 6 female) to 151 days (BSZ/*n *= 12 mice), including (*n* = 6male and *n* = 6 female); **(e)** from 130 days for age‐matched G37R males (control/*n* = 6 mice), to 150.5 days for age‐matched G37R males (BSZ/*n* = 6 males); (**f)** from 127 days for age‐matched G37R females (control/*n* = 6 mice), to 155.5 days for age‐matched G37R females (BSZ/*n* = 6 females). Data are presented as mean ± SEM, *n* = number of animals of the designated genotype. Data were analyzed using a Kaplan‐Meir Log‐rank (Mantel‐Cox) in SOD1‐G37R ALS mice, with a significance set at *p < 0.05*. Here, *p = 0.0001, p = 0.0006 and p = 0.0107* with ***p < 0.001, ***p < 0.001 and *p < 0.05 versus control G37R.

**TABLE 1:** The total number of age‐matched animals with segregation of sex in different cohorts (control and treatment) used in the study including assessment of various parameters (life span, disease onset, symptom onset, weight loss (%), with the total number of intraperitoneal injections (IP, IJ) was given to both cohorts.

To study the therapeutic effects of Edaravone prodrug (NS‐1‐2) in the SOD1‐G37R mouse model of amyotrophic lateral sclerosis Protocol# 21‐014, AC11693													
Control Treatment (N=12) including age‐matched (6 males and 6 females)							NS‐1‐2 Treatment (N=12) including age‐matched (6 males and 6 females)						
Animal ID	Sex	Life span/Age of survival (days)	Total number of IP, IJ	Age to reach disease onset (days)	Age to reach symptom onset (days)	Weight Loss (%) at humane endpoint	Animal ID	Sex	Life span/Age of survival (days)	Total number of IP, IJ	Age to reach disease onset (days)	Age to reach symptom onset (days)	Weight loss (%) at humane endpoint
**R651RR**	**Male**	**195**	**106**	**131**	**180**	**Weight Loss>25%** **Weight Loss (%): 25.324**	**R627R**	**Male**	**201**	**112**	**143**	**199**	**Weight Loss<25%** **Weight Loss (%): 17.056**
**R644LL**	**Male**	**184**	**95**	**129**	**169**	**Weight Loss<25%** **Weight Loss (%): 23.52**	**R711R**	**Male**	**201**	**112**	**143**	**171**	**Weight Loss<25%** **Weight Loss (%): 18.91**
**R624R**	**Male**	**179**	**90**	**140**	**156**	**Weight Loss>25%** **Weight Loss (%): 33.6**	**R715LR**	**Male**	**205**	**116**	**156**	**184**	**Weight Loss<25%** **Weight Loss (%): 19.67**
**R712LL**	**Male**	**170**	**81**	**131**	**158**	**Weight Loss<25%** **Weight Loss (%): 24.60**	**R736L**	**Male**	**189**	**100**	**147**	**180**	**Weight Loss<25%** **Weight Loss (%): 20**
**R751L**	**Male**	**184**	**95**	**128**	**152**	**Weight Loss>25%** **Weight Loss (%): 26.17**	**R739RR**	**Male**	**195**	**106**	**154**	**193**	**Weight Loss<25%** **Weight Loss (%): 15.68**
**R738LL**	**Male**	**162**	**73**	**125**	**154**	**Weight Loss>25%** **Weight Loss (%): 27.85**	**R635L**	**Male**	**182**	**93**	**168**	**182**	**Weight Loss<25%** **Weight Loss (%): 18.306**
**R647LLR**	**Female**	**186**	**97**	**136**	**149**	**Weight Loss>25%** **Weight Loss (%): 32.107**	**R638L**	**Female**	**198**	**109**	**164**	**195**	**Weight Loss<25%** **Weight Loss (%): 18.543**
**R697LL**	**Female**	**187**	**98**	**106**	**163**	**Weight Loss>25%** **Weight Loss (%): 26.923**	**R639R**	**Female**	**203**	**114**	**163**	**203**	**Weight Loss<25%** **Weight Loss (%): 10.122**
**R699R**	**Female**	**198**	**109**	**131**	**186**	**Weight Loss<25%** **Weight Loss (%): 23.58**	**R605L**	**Female**	**197**	**108**	**188**	**196**	**Weight Loss<25%** **Weight Loss (%): 13.66**
**R705LR**	**Female**	**150**	**61**	**123**	**142**	**Weight Loss>25%** **Weight Loss (%): 26.90**	**R700LR**	**Female**	**204**	**115**	**148**	**195**	**Weight Loss<25%** **Weight Loss (%): 18.548**
**R703LL**	**Female**	**201**	**112**	**159**	**189**	**Weight Loss<25%** **Weight Loss (%): 24.88**	**R701L**	**Female**	**174**	**85**	**144**	**165**	**Weight Loss<25%** **Weight Loss (%): 23.34**
**R634LL**	**Female**	**139**	**50**	**117**	**127**	**Weight Loss>25%** **Weight Loss (%): 30.081**	**R640LL**	**Female**	**167**	**78**	**144**	**160**	**Weight Loss>25%** **Weight Loss (%): 25.64**

### 2.8 Borsantrazole (BSZ) Delayed the Progression of Symptom Onset, Defined as Age to Reach 10% Weight Loss, With Muscle Weakness, in SOD1‐G37R Mouse Model of ALS

Symptom onset or early symptomatic stage was also assessed in treated and control mice by measuring the age to reach a 10% loss of bodyweight based on the highest recorded weight (with muscle weakness). As shown in Figure 11, daily intraperitoneal injection (IP, IJ) with BSZ starting pre‐symptomatically from the age of 90 days until the humane endpoint (end‐stage) delayed the progression of early symptom onset to a statistically significant degree (*p* < 0.05) in the age‐matched whole set of animals and in both sexes (male/female) of SOD1‐G37R ALS mice (*n* = 12/group, (6 male and 6 female) in both the vehicle‐treated (control) and BSZ treated group. The mean symptom onset age (loss of 10% body weight) was found to be statistically distinct between the vehicle‐treated (160.4 days) and BSZ‐treated group (185.3 days), representing a significant delay of around 24.9 days in the BSZ treatment group compared to the control group. On sex segregation, we found that BSZ‐treated age‐matched males (*n* = 6), and age‐matched females (*n* = 6), exhibited a delay of early‐symptom onset of 23.3 days and 26.4 days compared to the control group, respectively, with a slightly larger effect size in females by 3.1 days, nevertheless, males demonstrated a significant effect (*p* = 0.0030) compared to females (0.0622). The lack of statistical significance can be attributed to the greater range in age at 10% loss in bodyweight for control females. One female control lost 10% bodyweight at 127 days of age, compared to one control female who lost 10% bodyweight at 189 days of age, giving a range of 62 days, while for treated females the age range was only 43 days, from 160 days (earliest 10% loss female) to 203 days (latest 10% loss female). Interestingly, the range in ages for 10% loss of bodyweight (earliest to latest) for males was only 28 days in both the control and treated groups (152 days to 180 days control; 171 days to 199 days treated, respectively). Sex segregation showed a therapeutic effect of BSZ in males compared to females, in terms of statistical significance, suggesting a sex‐specific efficacy of BSZ. Although based on probability of symptom onset analysis using a Kaplan‐Meier Log‐rank (Mantel‐Cox), both sexes were found to be statistically significant (*p* = 0.0033 males, *p* = 0.0195 females). The symptom onset data is also in tabulated form (Table 1) below.

**FIGURE 11:**
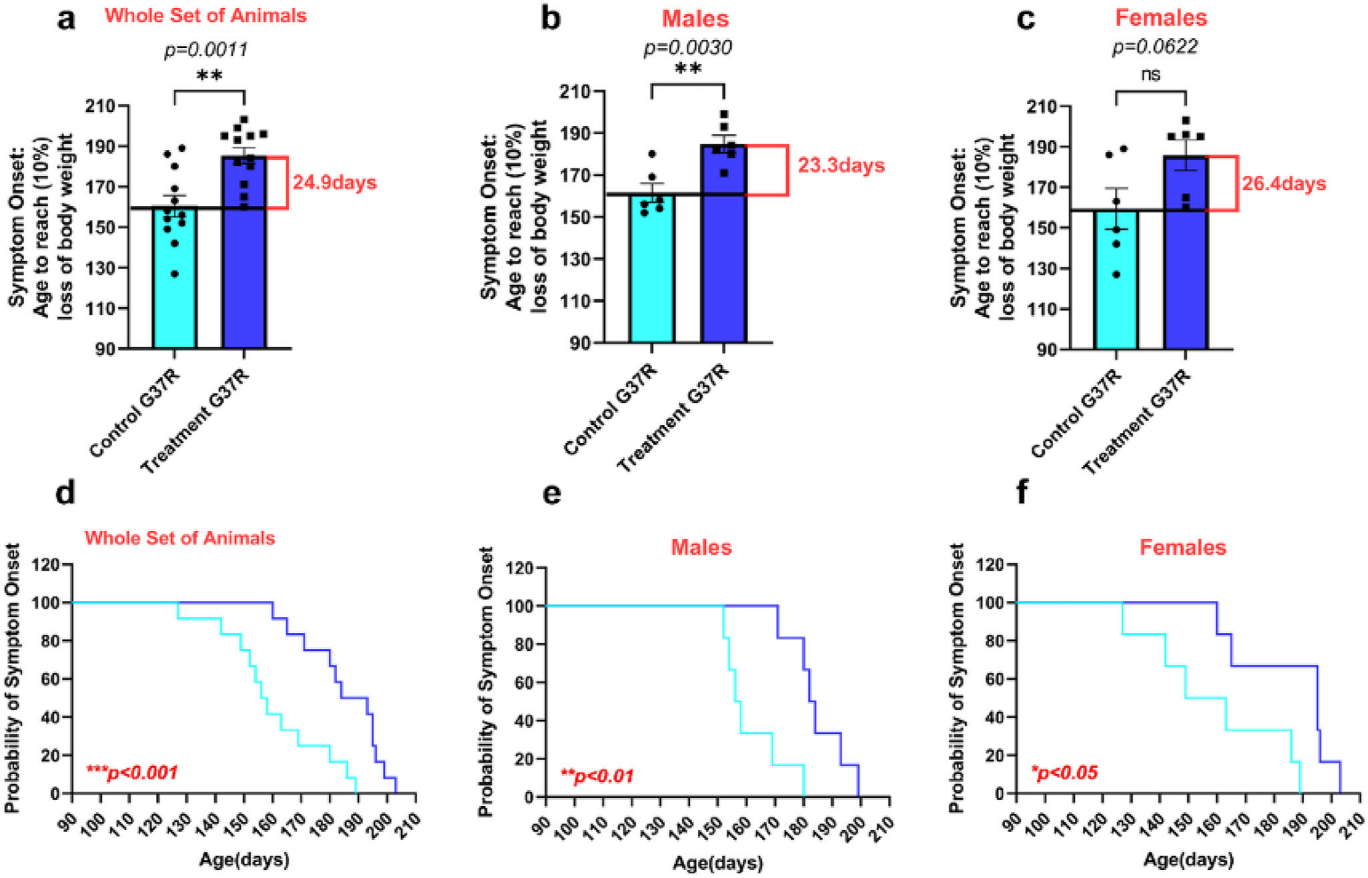
**Borsantrazole (BSZ) delays the symptom onset or early symptom progression (age to reach 10% weight loss, with muscle weakness). (a)** Two‐tailed (unpaired *t‐test*) of the mean onset age showing that BSZ delays the mean symptom onset age from 160.4 days for G37R age‐matched whole set of animals (control/*n* = 12 mice), including (*n* = 6 males and *n* = 6 females) to 185.3 days (BSZ/*n* = 12 mice), including (*n* = 6 males and *n* = 6 females); (**b)** from 161.5 days for age‐matched G37R males (control/n=6 mice), to 184.8 days for age‐matched G37R males (BSZ/*n* = 6 mice); (**c)** from 159.3 days for age‐matched G37R females (control/*n* = 6 females), to 185.7 days for age‐matched G37R females (BSZ/*n* = 6 females). Data are presented as mean ± SEM (*n* = 12), for the whole set of animals and (*n* = 6) for males and females. *n* = number of animals of the designated genotype. Data were analyzed using a two‐tailed (unpaired *t‐test*), with a significance set at *p < 0.05*. Here, *p = 0.0011, p = 0.0030 and p = 0.0622*, with **p < 0.01, **p < 0.01 and ns=non‐significant versus control G37R. (**d)** Kaplan–Meier curves of the probability of symptom onset showing that BSZ delays the median symptom onset from 157 days for age‐matched G37R whole set of animals (control/*n* = 12 mice), including (*n* = 6 male and *n* = 6 female) to 188.5 days (BSZ/*n* = 12 mice), including (*n* = 6 males and *n* = 6 females); **(e)** from 157 days for age‐matched G37R males (control/*n* = 6 mice), to 183 days for age‐matched G37R males (BSZ/*n* = 6 males); (**f)** from 156 days for age‐matched G37R females (control/*n* = 6 mice), to 195 days for age‐matched G37R females (BSZ/*n* = 6 females). Data are presented as mean ± SEM. *n* =  number of animals of the designated genotype. Data were analyzed using a Kaplan‐Meier Log‐rank (Mantel‐Cox) in SOD1‐G37R ALS mice, with a significance set at *p < 0.05*. Here, *p = 0.0009, p = 0.0033 and p = 0.0195* with ***p < 0.001, **p < 0.01 and *p < 0.05 versus control G37R.

### 2.9 Borsantrazole (BSZ) Prolongs the Life Span and Increases Survival in the SOD1‐G37R Mouse Model of ALS

As part of our current preclinical studies, we initiated the treatment of EDR analogue BSZ pre‐symptomatically at the age of 90 days until the age of 210 days, and monitored daily longitudinally for several significant humane endpoint indicators throughout ALS disease progression including: (a) Mouse unable to right itself in 15 s; (b) 25% loss of weight on the highest recorded weight; (c) Full paralysis of one or more hind limbs; (d) Loss of bladder functions; (e) Crusty eyes/loss of vision; and (f) Penile prolapse. Further, the minimum primary humane endpoint of survival/lifespan/end stage of the mice was defined as the point when the mice were unable to right themselves in 15 s, with some hind limb paralysis, or lost 25% of weight, which may also be accompanied by several other humane endpoint indicators of disease progression, as listed above.

As shown in Figure 12, daily intraperitoneal injection (IP, IJ) with BSZ starting pre‐symptomatically from the age of 90 days until the humane endpoint (end‐stage) significantly prolongs the life span (*p* < 0.05) in the age‐matched whole set of animals and in SOD1‐G37R ALS male mice. For the whole set of animals, *n* = 12/group, including 6 male and 6 female both in the vehicle‐treated (control) and BSZ‐treated group, the mean age of survival differed significantly between the vehicle‐treated (177.9 days) and the BSZ‐treated group (193 days). Further, the age of survival was significantly prolonged by around 15.1 days in the BSZ treatment group compared to the control group. On sex segregation, we found that BSZ‐treated age‐matched males (*n* = 6), and age‐matched females (*n* = 6) displayed an extension of life span (16.5 days) and (13.7 days) compared to the control group, respectively, with a larger effect size in males compared to females of 2.8 days. Statistically, males met significance with *p* = 0.0192, while females had *p* = 0.2972. The age range at endpoint for control males was 33 days, and treated males was 23 days, while for female controls it was 62 days, compared to treated females at 37 days, from earliest age at endpoint to latest age at endpoint (male controls, 162 days vs. 195 days; male treated, 182 days vs. 205 days; female controls, 139 days vs. 201 days; female treated 167 days vs. 204 days). Therefore, the lack of statistical significance in females can largely be attributed to the greater range in ages at endpoint for the control females, which have a much greater variability in ages at endpoint. Overall, sex segregation demonstrated a therapeutic effect of BSZ in males compared to females when considering a small extension in survival (2.8 days) and statistical significance, suggesting a sex‐specific efficacy of BSZ. Once again, upon closer inspection of the female cohort, the lack of statistical significance was largely due to the variance in the life span of female controls, ranging from 139 to 201 days (62 day span), as compared to male controls (162 to 195 days, 33 day span), whereas the range in ages for the female treated group was only 37 days, compared to 23 days for treated males. The survival age data are also in tabulated form (Table 1) below.

**FIGURE 12:**
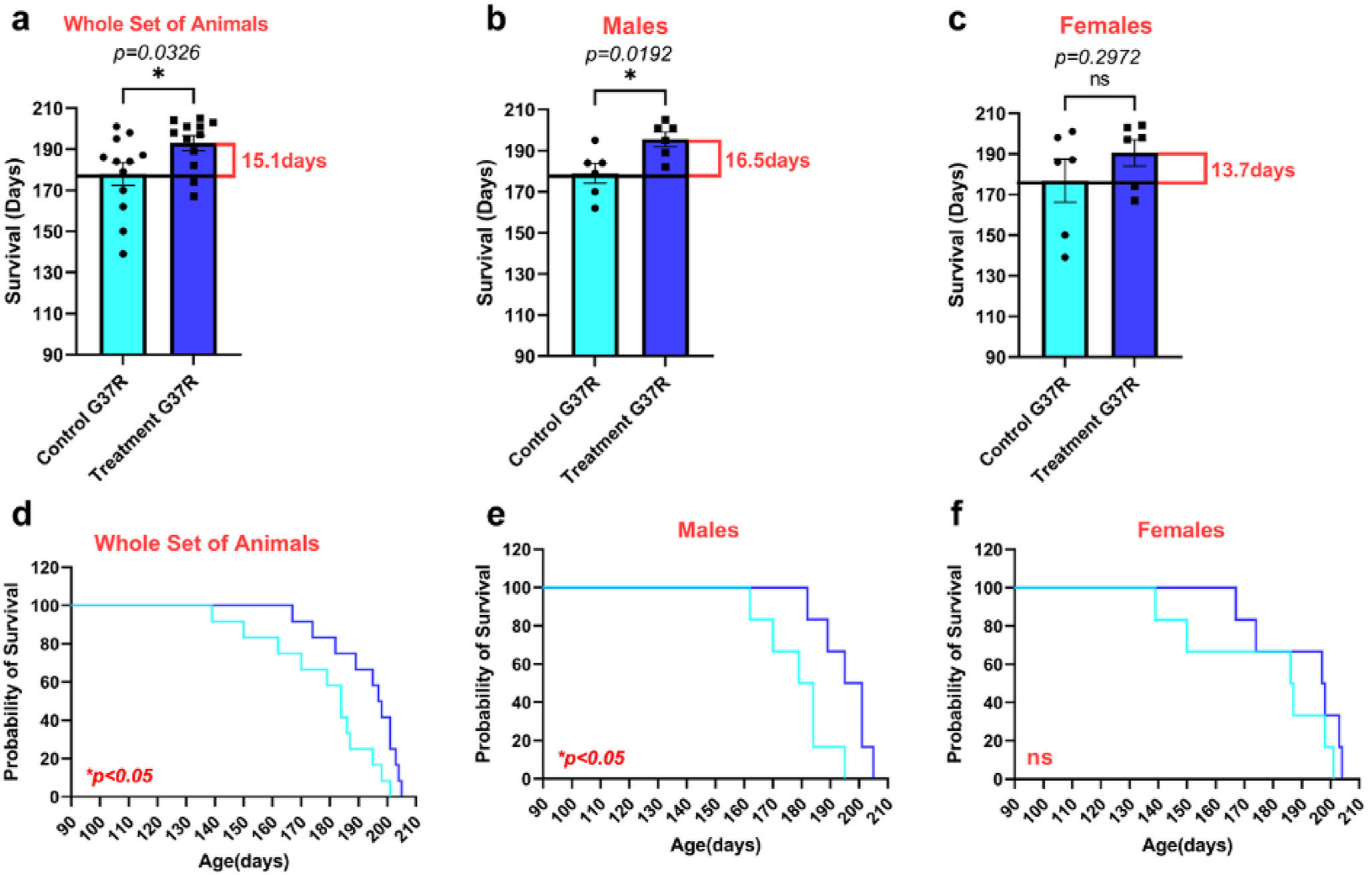
**Borsantrazole (BSZ) extends the life span in SOD1‐G37R ALS mice. (a)** Two‐tailed (unpaired *t‐test*) of the mean onset age showing that BSZ delays the mean symptom onset age from 177.9 days for G37R age‐matched whole set of animals (control/*n* = 12 mice), including (*n* = 6 males and *n* = 6 females) to 193 days (BSZ/*n* = 12 mice), including (*n* = 6males and *n* = 6 females); (**b)** from 179 days for age‐matched G37R males (control/*n* = 6 mice), to 195.5 days for age‐matched G37R males (BSZ/*n* = 6 mice); (**c)** from 176.8 days for age‐matched G37R females (control/*n* = 6 females), to 190.5 days for age‐matched G37R females (BSZ/*n* = 6 females). Data are presented as mean ± SEM (*n* = 12), for the whole set of animals and (*n* = 6) for males and females. *n* = number of animals of the designated genotype. Data were analyzed using a two‐tailed (unpaired *t‐test*), with a significance set at *p < 0.05*. Here, *p =0.0326 (whole set), p = 0.0192 (male) and p = 0.2972 (female)*, with *p < 0.05 and ns=non‐significant versus control G37R. (**d)** Kaplan–Meier curves of the probability of symptom onset showing that BSZ prolongs the median the median survival from 184 days for age‐matched G37R whole set of animals (control/*n* = 12 mice), including (*n* = 6male and *n* = 6 female) to 197.5 days (BSZ/*n* = 12 mice), including (n=6male and n=6 female); **(e)** from 181.5 days for age‐matched G37R males (control/*n* = 6 mice), to 198 days for age‐matched G37R males (BSZ/*n* = 6 males); (**f)** from 186.5 days for age‐matched G37R females (control/*n* = 6 mice), to 197.5 days for age‐matched G37R females (BSZ/*n* = 6 females). Data are presented as mean ± SEM. *n* = number of animals of the designated genotype. Data were analyzed using a Kaplan‐Meir Log‐rank (Mantel‐cox) in SOD1‐G37R ALS mice, with significance set at *p <0.05*. Here, *p = 0.0174 (whole set), p = 0.0232 (males) and p = 0.2322 (females)* with *p < 0.05 and ns versus control G37R.

### 2.10 Borsantrazole (BSZ) Prevents ALS‐Induced Weight Loss (Cachexia) in the SOD1 Linked Model of ALS

Initiating the treatment of EDR prodrug BSZ pre‐symptomatically at the age of 90 days until the age of 210 days modified the clinical disease phenotypes of ALS‐induced cachexia. Weight loss is regarded as the clinical prognostic marker for ALS disease progression due to muscle loss, fat loss, decreased caloric intake, and larger metabolic demand [87]. During the clinical progression of the disease after disease onset, the loss of motor neurons causes denervation, which leads to loss of muscle fibers resulting in a decrease in muscle mass. Rapid denervation‐induced weight loss has been recognized as an indicator of faster progression and shorter survival [87]. As part of our current animal studies, we evaluated weight loss from highest recorded weight to weight at humane endpoint/end stage for control versus treated. Body weight was monitored daily longitudinally starting from age 90 days until the age 210 days for a total of four months.

As shown in Figure 13, daily intraperitoneal injection (IP, IJ) with BSZ starting pre‐symptomatically from the age of 90 days until the humane endpoint (end‐stage) significantly prevented weight loss in the age‐matched whole set of animals and both sexes (male/female) of SOD1‐G37R ALS mice. For the whole set of animals, *n* = 12/group, including 6 male and 6 female in both the vehicle‐treated (control) and BSZ‐treated group. The mean percentage loss of weight differed significantly between the vehicle‐treated (27.41%) and BSZ‐treated group (18.2%), representing a reduction in the overall percent weight loss in the BSZ treatment group compared to the control of 8.838%. On segregation of sex, we found that BSZ‐treated age‐matched males (*n* = 6), and age‐matched females (*n* = 6) demonstrated significant prevention of percentage weight loss (8.574%) and (9.103%) compared to the control group respectively, with a larger effect size in males (*p* = 0.0004) compared to females (*p* = 0.0071). The survival age data are also in tabulated form (Table 1) below.

**FIGURE 13:**
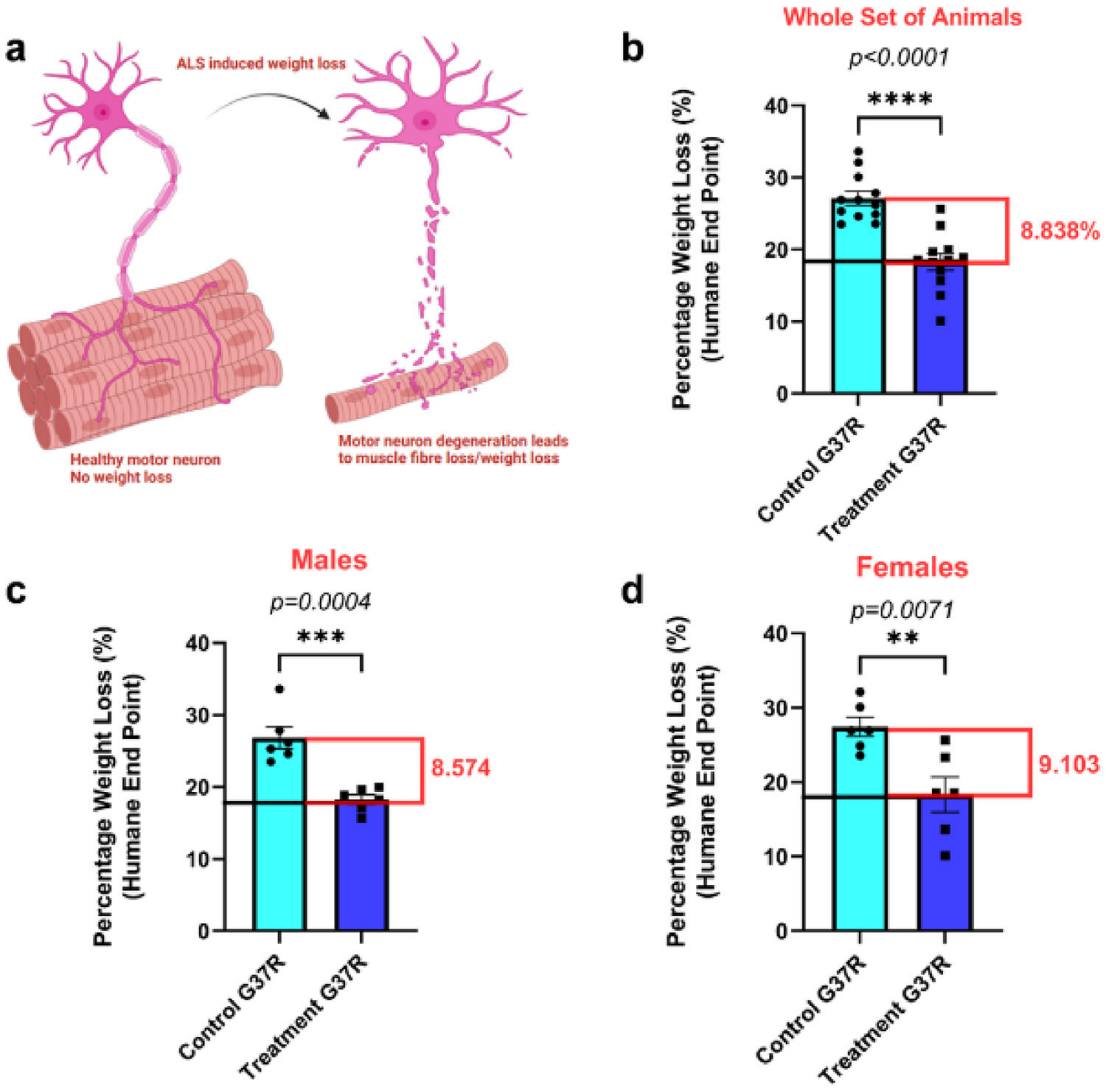
**Borsantrazole (BSZ) rescued weight loss in SOD1‐G37R ALS mice. (a)** Photograph showing progressive loss of motor neurons leads to loss in muscle fiber and hence, weight loss in ALS. (**b)** Two‐tailed (unpaired *t‐test*) of the mean percentage loss of body weight at the humane endpoint showing that BSZ prevented weight loss from 27.13% for G37R age‐matched whole set of animals (control/*n* = 12 mice) (including *n* = 6 males and *n* = 6 females) to 18.29% (BSZ/*n* = 12 mice), including *n* = 6 males and *n* = 6 females. (**c)** Weight loss of 26.84% for age‐matched G37R males (control/*n* = 6 mice), to 18.27% for age‐matched G37R males (BSZ/*n* = 6 mice). (**d)** Weight loss of 27.41% for age‐matched G37R females (control/*n* = 6 females), to 18.31% for age‐matched G37R females (BSZ/*n* = 6 females). Data are presented as mean ± SEM (*n* = 12), for the whole set of animals and (*n* = 6) for males and females. *n* = number of animals of the designated genotype. Data were analyzed using a two‐tailed (unpaired *t‐test*), with a significance set at *p < 0.05*. Here, *p = 0.0001, p = 0.0004 and p = 0.0071* with ***p < 0.001, **p < 0.001 and *p<0.05 versus control G37R.

### 2.11 Borsantrazole (BSZ), with Longitudinal Monitoring, Improves Several Motor Clinical Phenotypes in the SOD1‐G37R Mouse Model of ALS

Presymptomatic treatment with BSZ, starting at the age of 90 days until the age of 210 days with daily monitoring for four months for several humane endpoint indicators, ameliorated various clinical motor decline phenotypes in ALS mice, compared to vehicle‐treated mice. BSZ improved several observed markers for motor neurodegeneration during disease progression in SOD1‐G37R ALS mice in terms of hindlimb clasping behavior, abnormal hindlimb flexion, and abnormal splay of the toe fingers Figure 14.

**FIGURE 14:**
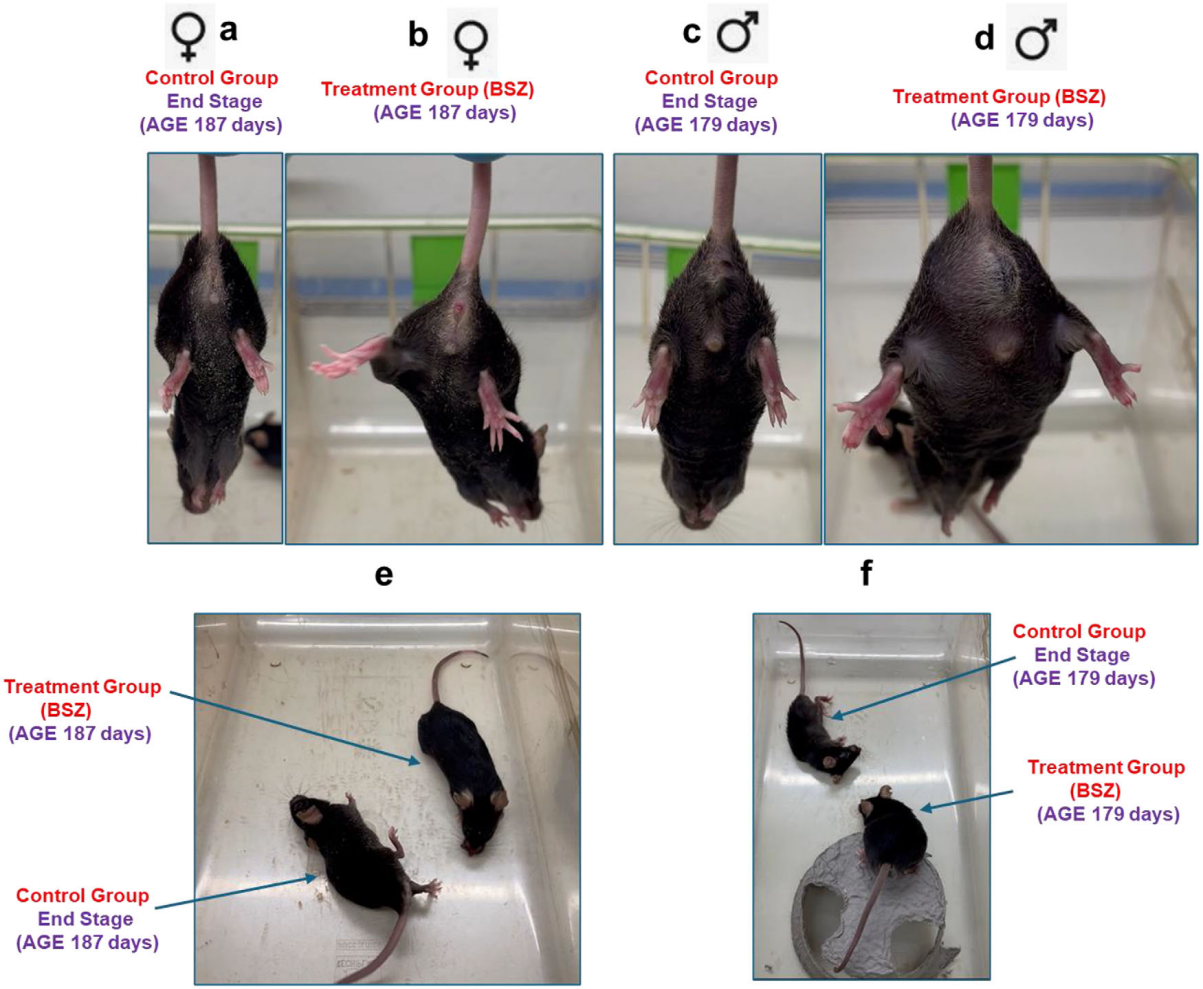
**Representative pictures showing the longitudinal assessment of various gross motor clinical phenotypes and the effects of treatment BSZ in modifying the motor disease clinical phenotypes in G37R ALS mice**. Age‐matched littermates (control vs treatment (BSZ)) showing various ALS clinical phenotypes/ALS Motor functions phenotypes/disease progression. When the female and male mice were suspended by their tail at the age of (187 and 179 days), respectively, the vehicle‐treated control groups (**a)**, and (**c)**, showed immobile hindlimbs (no limb flexion) and hindlimbs falling toward midline of the abdomen (abnormal splay), whereas BSZ treatment groups (**b)** and (**d)** showed mobile hindlimbs (limbs flexion), with hindlimbs splayed outwards away from the abdomen. Both the male and female vehicle‐treated/control groups were unable to right themselves in 15 s compared to the BSZ treated group, at the age of 187 days (**e**), and at the age of 179 days (**f**).

### 2.12 Proteomic and Phosphoproteomic Analysis Reveals Differentially Regulated Proteins in the Lumbar Spinal Cord Samples from the SOD1‐G37R Mouse Model of ALS Correlating with the Efficacy of BSZ

In an attempt to explore and rationalize the therapeutic efficacy of the novel boron‐based pyrazole BSZ, and to further move forward in our drug development process, we utilized a discovery‐based LC‐MS/MS detection technique to profile the global changes in the proteome and phosphoproteome of mutant human SOD1‐G37R mice spinal cord samples (*n = 4*) from both control and treated groups. Considering the significant efficacy results in the male cohort, we assessed the proteomic signatures of late‐stage ALS male mice only, which is one of the limitations of our study (male sex specificity). A total of 6270 proteins were quantified in all replicates of either sham (vehicle‐treated) and BSZ‐treated samples. Differential analysis was performed using a moderated *t‐test* (LIMMA) [88]. Intensities were log2‐transformed, followed by K‐nearest neighbor (KNN) imputation for Missing at Random (MAR) values. A total of 51 proteins (with adjusted *p*‐values <0.05) were found to be differentially expressed to a statistically significant degree between treated and sham groups. (see Supplementary Data for full proteomics data).

Three known ALS‐relevant proteins **Cplx2, Lrp4, and Sqstm1/p62** were significantly increased in the BSZ‐treated group, while **Ca3** showed a significant decrease (see Table 2). These proteins are found to have a pathological link with the progression of ALS based on existing literature, and these differential expressions of proteins represent a positive correlation with the efficacy of BSZ in terms of extension of survival, delay in onset, delay in symptom onset, and rescuing weight loss. It is of interest to note that increased Ca3 has been recently found to be a biomarker of ALS pathology, vide infra. We further discovered increased expressions of two proteins **(Gan/KLHL16, Snx13)** in the treatment (BSZ) group compared to the control group. These two proteins are not specific to ALS; however, their increased expression is implicated in the pathophysiology of neurodegenerative disease and in maintaining neuronal health Table 2. The differential regulation of these significant proteins is highlighted in the volcano plot (Figure 15b). Additionally, the heatmap of identified differentially expressed proteins (DEP) displayed consistent patterns of upregulated or downregulated proteins among the significantly regulated proteins for BSZ and the sham (control) group (Figure 15c).

**TABLE 2:** Selected known and novel (ALS‐associated) proteins with significantly differential abundance (adj *p < 0.05*; *n = 4*) in the lumbar spinal cord of human mutantSOD1‐G37R male mice treated with BSZ and sham (vehicle) based on quantitative proteomics.

UniProt accession number (Protein ID)	Abbreviated (Gene Symbol)	Protein description	adj. p‐value	ratio (BSZ/Sham)	Pathological Association to ALS Known/Novel
P16015	***Ca3***	Carbonic anhydrase 3	**0.0279**	**0.3**	**Known**
Q8CA72	***Gan/KLHL16***	Gigaxonin	**0.0219**	**2.7**	**Novel**
P84086	***Cplx2***	Complexin‐2	**0.00565**	**1.9**	**Known**
Q6PHS6	***Snx13***	Sorting nexin‐13	**0.00144**	**2.5**	**Novel**
Q8VI56	***Lrp4***	Low‐density lipoprotein receptor‐related protein 4	**0.01020**	**3.6**	**Known**
Q64337	***Sqstm1/p62***	Sequestosome‐1	**0.01140**	**3.0**	**Known**

**FIGURE 15:**
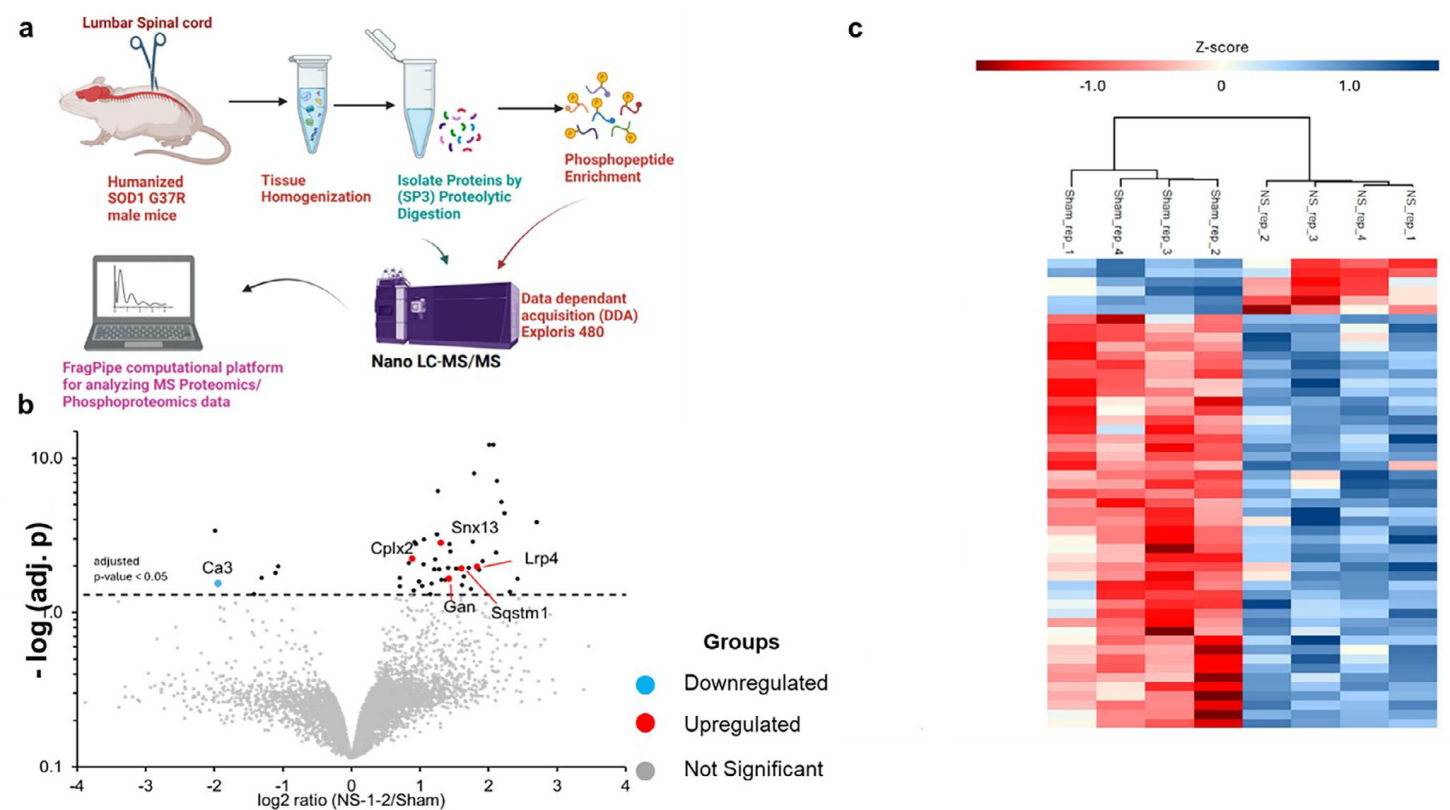
**(a,b,c). Differential analysis global proteome changes in SOD1‐G37R ALS mice. (a)** Overview of the sample processing workflow for LC/MS‐based global proteomics and phosphoproteomics. **(b)** Volcano plots showing the differential regulation of proteins expressed in the lumbar spinal cord of SOD1‐G37R male mice. Based on log2‐transformed intensities, 51 out of 6270 proteins were significantly differential (adj. *p < 0.05*; *n = 4*). Selected proteins that are either up (red) or down (blue) regulated compared to controls are highlighted. (**c)** Heat map showing unsupervised hierarchical clustering of z‐scored log2 intensities of the 51 significantly regulated proteins for BSZ and sham (control) group. Heatmap generated with Perseus 2.0.1. *Note*: NS‐1‐2 refers to BSZ in Figure 14 (a, b, c). NS‐1‐2 was initially developed in the lab and later trademarked as Borsantrazole (BSZ).

A total of 9541 phosphorylation sites were quantified and further filtered to retain high‐quality data. Only phosphopeptides that were quantified in all 4 replicates of either treated or sham were kept, resulting in a total of 3945 high‐quality phosphopeptides. Differential analysis was performed using a moderated *t‐test* (LIMMA) [88]. Intensities were log2‐transformed, followed by K‐nearest neighbor (KNN) imputation for Missing at Random (MAR) values. A total of 29 phosphorylation sites with adjusted *p*‐values <0.05 were considered statistically significantly differential between treated and sham groups (see Supporting Information for full list).

Our phosphoproteomics data discovered new phosphorylation sites for known protein biomarkers of ALS progression, neurofilament light and heavy chain (Nefl and Nefh). We found that 58 and 8 phosphorylated serine/threonine (S/T) sites are downregulated for Nefh and Nefl, respectively, following treatment with BSZ. These results are reinforced when reflecting on the general slight reduction in protein expression also detected in our proteomics data (Nefh and Nefl), which is mirrored by the decreases in phosphorylation. Overall, this observable trend in downregulation of neurofilament light and heavy chains correlates with the efficacy observed in our SOD1‐G37R mouse model, among treated animals, and is representative of the benefits of BSZ treatment using a key marker of ALS disease progression (Nefh and Nefl). Importantly, phosphorylation of **T72** in Nefh and **T317** in Nefl was significantly reduced in the BSZ‐treated group. Phosphoglycerate kinase 1 (S203) was also found to be downregulated in the treatment group, with Pgk1 being a key enzyme in glycolysis. Phosphorylation of S203 can be induced by EGFR activation or hypoxia and is a biomarker of greater dysregulation of cell metabolism in the sham group. Further details on proteomic and phosphoproteomic results will be published in an alternate manuscript in the near future. (Table 3; see Supporting Information for full phosphoproteomics data).

**TABLE 3:** Summary of known and novel proteins with significantly differential phosphorylation (adj. *p < 0.05*; *n = 4*) in the lumbar spinal cord of mutant SOD1 G37R male mice treated with BSZ and sham (vehicle), based on quantitative phosphoproteomics. For a full summary of the phosphoproteomics data for BSZ and Sham (vehicle) samples, see Supporting Information.

UniProt accession number (Protein ID)	Abbreviated (Gene Symbol)	Protein description	Phosphorylation Site	adj. p‐value	ratio (BSZ/Sham)
P19246	***Nefh***	Neurofilament heavy polypeptide	**T72**	7.73E‐03	<0.1
P09411	***Pgk1***	Phosphoglycerate kinase 1	**S203**	2.23E‐03	0.4
P08551	***Nefl***	Neurofilament light polypeptide	**T317**	4.72E‐02	0.3

## 3 Discussion

A large number of investigations, both in SOD1 ALS mouse models and in sALS and fALS ALS patients, have elucidated the role oxidative stress plays in the pathophysiology of ALS. Therefore, alleviating or attenuating oxidative stress is an attractive therapeutic target and represents both a viable strategy for slowing down the death of motor neurons during ALS progression and as a means to discover novel ALS therapeutics [89]. To date, several antioxidant compounds have been tested in human ALS clinical trials, yet none of the compounds have succeeded in meeting a primary endpoint of extending survival [90]. Based on results from a pivotal phase 3 clinical trial in Japan, EDR is the only small molecule antioxidant that demonstrated improvement in the physical functional decline of ALS patients, as measured by the ALSFRS‐R score and improved quality of life as measured by the ALS 40‐item assessment questionnaire [91]. However, EDR has failed to show a significant extension in the life span of ALS mice [18] and ALS patients [92], although EDR was successful in translating slower disease progression from SOD1 ALS mice models to ALS patients.

In our laboratory, for the first time, we have developed an alternate greener route for the synthesis of EDR. Until now, all reported procedures for the synthesis of EDR utilize a Knorr pyrazole synthesis, using phenyl hydrazine, a hazardous chemical, as a starting material. Our current synthetic procedure has many advantages over the conventional route. First, the use of greener reagents that are biocompatible, such as boronic acid (isopropoxyboronic acid pinacol ester), lactic acid, and H_2_O_2_. The second is energy use minimization, as the reaction was carried for 10 min at 50°C in a microwave synthesizer, in comparison to the prolonged heating at reflux, found in the classical process for 2–4 h, which causes degradation of EDR. Overall, using the above procedure, the typical yield for the synthesis of EDR, following recrystallization, is 85+%.

Overall, our current novel approach and strategy allow BSZ to exert a triple mode of therapeutic action with the added advantage of targeted drug delivery, Figure 16. First, BSZ is chemoselectively oxidized by high or pathological concentrations of H_2_O_2_, and proportionately reducing H_2_O_2_‐mediated neurotoxicity, and associated neurodegeneration. Second, H_2_O_2_‐mediated boronate oxidation results in the generation of potent FDA‐approved antioxidant EDR, which confers its antioxidant activities and therapeutic nature to the neurons undergoing oxidative stress. Third, the by‐product of this biotransformation is boric acid, which, according to emerging reports, acts as an antioxidant to reduce cellular oxidative damage [93]. Recent reports have shown that boric acid is known to activate Nrf2, an antioxidant response element [94]. Further, boric acid reduces oxidative stress by increasing the levels of thiol‐containing antioxidant glutathione [95]. In addition to the triple‐role antioxidant effects, BSZ will also act as a targeted therapy for quenching excessive H_2_O_2_, as its biotransformation would preferentially take place within regions of high oxidative stress, a known cause of neurodegeneration. This gives the additional advantage of targeting regions of both upper and lower motor neurodegeneration where oxidative stress is implicated as a primary culprit. Additionally, other free radicals are implicated in the pathology of ALS, and we propose that these free radicals (such as hydroxyl radicals (HO•), lipid peroxides, or superoxide) also produce EDR through a redox reaction between the reactive oxygen species (ROS) and boron. Appropriately, we propose that H_2_O_2_ should serve as the main in vivo ROS that transforms BSZ into EDR (prodrug action) as H_2_O_2_ has a long half‐life and is more stable than other ROS oxidants, whilst also being the product of superoxide dismutase (SOD1) and mutant SOD1, whose toxic gain of function is a predominant cause of fALS. Additionally, we would like to emphasize that BSZ should be especially effective at targeting regions of high H_2_O_2_ production and/or high SOD1 activity, such as in patients with mutant SOD1 fALS/sALS. The targeted nature of BSZ provides some notable advantages over EDR, such as tissue specificity (brain/spinal cord, where H_2_O_2_ is known to be highly active) and stimulus sensitivity (target regions of high oxidative stress), which should enhance the efficacy of the drug, with a decrease in side effects. H_2_O_2_ tends to accumulate in cells in high concentrations that are experiencing oxidative stress, resulting in a redox imbalance and hence, causing impaired redox signaling cascades vital for maintaining healthy cells, and often resulting in neurodegeneration. For these reasons, the pathological concentration of H_2_O_2_ is a valid and attractive target for neurodegenerative diseases like ALS (and other redox‐sensitive diseases). BSZ, being an H_2_O_2_ reactive prodrug, will be activated specifically in regions undergoing overproduction of cellular H_2_O_2_, as demonstrated during the pathophysiology of ALS [58, 96], and should provide added therapeutic benefits, compared to non‐targeting EDR.

**FIGURE 16:**
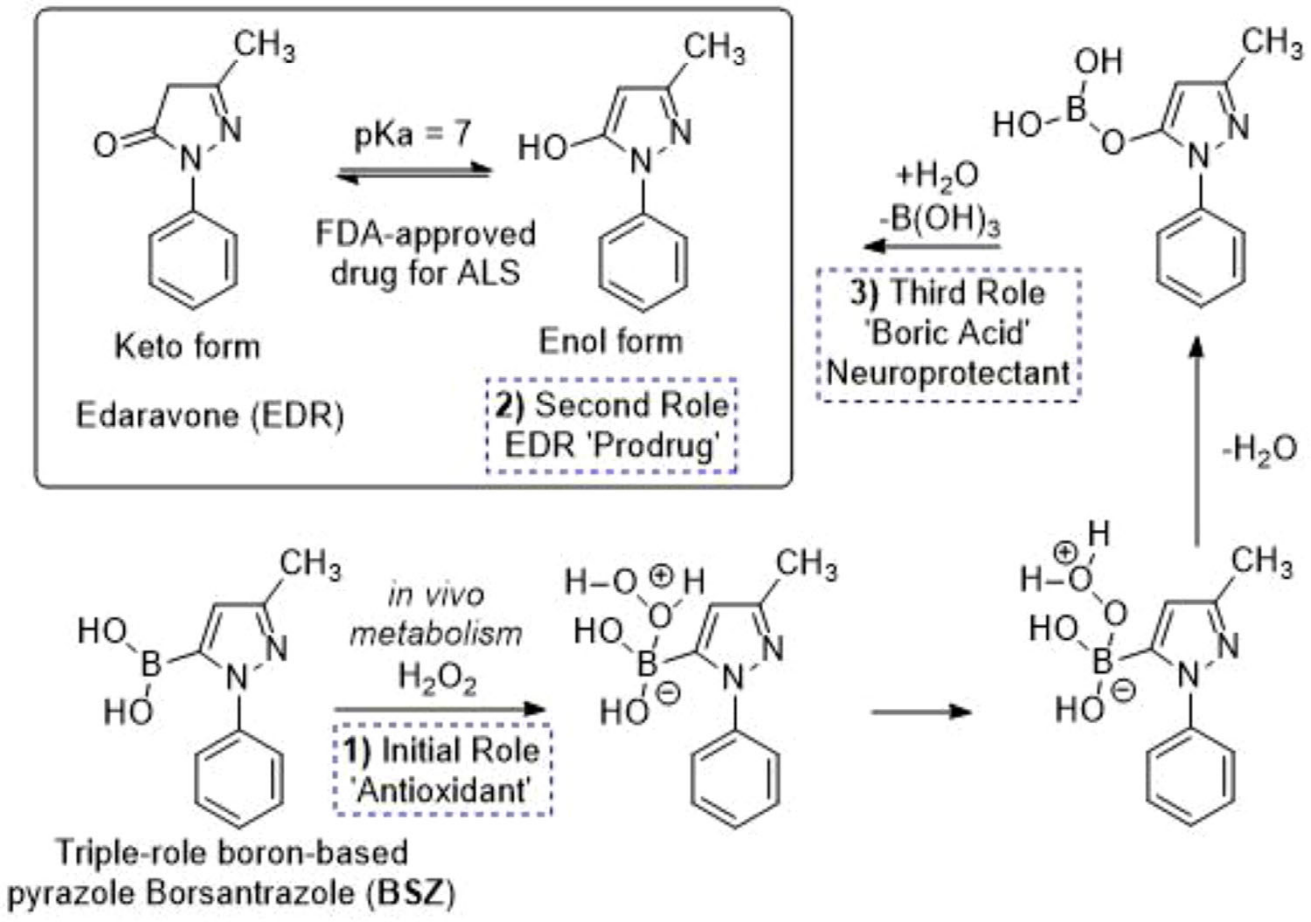
**Representative schematic illustration of the targeted delivery of a triple‐role boron‐based pyrazole BSZ (boronic acid form shown) as an ALS therapeutic**. BSZ, (a boronic acid pinacol ester and boron‐based prodrug of EDR) initially serves as an antioxidant by chemoselective in vivo reaction with reactive oxygen species, such as H_2_O_2_, which results in the generation of EDR (secondary role). Advantageously, the oxidative reaction also results in the production of boric acid, a known neuroprotectant, as tertiary role.

In the current study, we have synthesized a ROS‐responsive prodrug [97] of EDR by taking advantage of boron as the key mediator for targeting neurodegeneration. Our initial conjecture was that BSZ would chemoselectively target the pathological concentration of neurotoxic free radical H_2_O_2_ [98] known to cause oxidative stress and misfolding of SOD1 during the progression of ALS [52, 63, 64]. We therefore investigated the ability of EDR prodrugs to scavenge H_2_O_2_ using Amplex Red assay, Figure 3. The addition of EDR and BSZ at the concentration of (500–120 µm) resulted in a significant reduction in the concentration of neurotoxic H_2_O_2_ supporting our belief that BSZ readily reacts following biorthogonal chemistry with H_2_O_2_ to render efficient removal of H_2_O_2_ in the biochemical assays. Thus, this initial evidence of the efficient H_2_O_2_ scavenging ability of BSZ gave us encouragement to move toward in vivo experiments in ALS animal models.

Furthermore, we evaluated the safety profile of EDR and prodrug of EDR, BSZ in NSC‐34 cells and PCNC cells, Figure 4a,b. In the present study, we first asked the question of whether BSZ and EDR are safe in motor neuron‐like systems in vitro and induce no neurotoxicity. To test the effects on cell viability of BSZ and EDR, using the WST‐8 metabolism endpoint, we used the neuroblastoma spinal cord hybrid cell line (NSC‐34), which is a model for spinal cord motor neuron studies and is accepted for studying cellular‐level ALS pathology [99]. We found that both EDR and BSZ demonstrated excellent cell viability at around 90%–100% in the NSC‐34 cells with no neurotoxicity. Furthermore, to make this analysis more reliable and to support the observed safety profile of BSZ in NSC‐34 cells, we used primary cortical neurons (PCNC) prepared from embryonic day 17‐18 (E17‐E18) mouse brain; equivalent to the third trimester of a human pregnancy and exhibited various unique features that cannot be adequately mimicked by cell lines. We found that lower concentrations (10 and 1 µm) of BSZ showed good cell viability of around (90%–100%) on PCNC and is similar to that of EDR, however, higher concentrations of 25 µM of EDR and BSZ showed around (70% ± 10%) cell viability on PCNC.

In addition, we also analyzed the neuroprotective role of EDR and BSZ in NSC‐34 cells against neurotoxic H_2_O_2_ challenge, Figure 4c. The major ROS involved in the pathophysiology of ALS is H_2_O_2_ [64]. The pathological concentration of H_2_O_2_ is known to cause damage to neurons and further creates an oxidizing cellular environment by initiating Fenton's reaction and Heber‐Weiss reaction, leading to the generation of the highly reactive oxidant, hydroxyl radical (HO•), regarded as one of the most reactive free radical, subsequently causing neuronal damage and death [57]. In the present study, we also used H_2_O_2_ neurotoxin to generate an in vitro model of oxidative stress. The prophylactic treatment of both EDR, and its prodrug BSZ, significantly attenuated H_2_O_2_‐induced neurotoxic insults, maintaining neuronal viability. The prodrug BSZ demonstrated higher neuronal viability at lower doses of (1 and 25 µm), showing that BSZ is more potent than EDR, on the other hand, BSZ provided equal neuroprotection at higher concentrations (50 and 100 µm). Overall, we discovered that the EDR prodrug, BSZ, has a safe profile in NSC‐34 and PCNC cells and exerted a neuroprotective effect against H_2_O_2_‐induced neurotoxicity in NSC‐34 cells.

Subsequently, we evaluated the safety profile of the prodrug of EDR, BSZ, in wild‐type animals. We determined the safety of BSZ with a single dose of 10 mg/kg bodyweight (acute toxicity, Figure 7a,b) and 120 daily doses (chronic toxicity, 10 mg/kg bodyweight/day, Figure 7c,d) with longitudinal monitoring. According to regulatory authorities, a decrease in body weight acts as a reliable, sensitive, and powerful indicator for determining chemical toxicity, therefore, we analyzed the treatment‐associated change in any body weight after treatment for 14 days in the case of acute toxicity following a single dose and for 120 daily doses in the case of chronic toxicity. Further, we also monitored any symptoms associated with the treatment. Additionally, we considered to deduce the sex‐specific differences in toxicity in both male and female mice. Both the acute and chronic safety evaluation with BSZ demonstrated no symptoms of acute treatment‐associated toxicity in both sexes. Second, no treatment‐associated decrease in body weight changes in the whole set of animals, and lastly, no signs of treatment‐associated hematological toxicity (Figures 7 and 8) among the sexes were observed. It is noteworthy to further highlight that the results obtained from both the acute and chronic toxicity studies demonstrate that the average percentage weight change from baseline indicates that the BSZ group, in both acute and chronic toxicity evaluations, did not demonstrate a reduction in body weight compared to the control group. This observation suggests that there are no indications of toxicity associated with BSZ at the end of the study in either group (Figures 7b,d). We also propose that during the metabolic biotransformation of BSZ to EDR in vivo, the major by‐product of the biotransformation is boric acid and pinacol. The LD_50_ of pinacol in mice is 3,380 mg/kg oral dose [100], and the LD_50_ of boric acid in rats is 2660 mg/kg [100]. In addition, the daily acceptable intake of boron recommended by the WHO for adults is 1–13 mg/d. This clinical data suggests that side effects due to the boronate scaffold in BSZ should be minimal or non‐existent [101]. The excellent preliminary safety profile of BSZ gives us confidence to move further ahead with our efficacy study in ALS mice models.

Moreover, in the present preclinical study, we evaluated the disease‐modifying effects of an EDR prodrug BSZ in the humanized SOD1‐G37R mice model of ALS. Considering the neuroprotective role of BSZ in in vitro assays and its satisfactory safety profile in both in vitro and in vivo models, gave us an impetus to test the efficacy of BSZ in the SOD1‐G37R ALS mice model. We analyzed the disease‐modifying effects of a newly synthesized prodrug of EDR called BSZ in high copy number, early onset, and fast progressor hmutantSOD1‐G37R familial mice model of ALS. Long‐term treatment of BSZ significantly delays the disease onset and symptom onset in both sexes (male/female) of ALS mice. However, the magnitude of delay in disease onset and symptom onset is diminished in males compared to females, although with greater statistical significance in males. Further, long‐term treatment of a novel molecule (BSZ) significantly rescued ALS‐induced weight loss (cachexia) in both sexes (male/female), Figure 13. However, the observed weight loss (male, *p = 0.0004* vs female, *p = 0.0071*) demonstrates a larger clinical effect size in females compared to males. In addition, long‐term treatment of BSZ demonstrated an extension in survival in both sexes. BSZ treatment significantly prolongs the life span of male ALS mice (16.5 days), and the whole set of animals overall (15.1 days), Figure 12. Although the increase in survival in females (13.7 days) was not found to have *p* < 0.05, the lack of statistical significance was largely due to the variance in the life span of female controls, ranging from 139 to 201 days (62 day span), whereas the range in ages for the female treated group was only 37 days. Overall, it appears that female SOD1‐G37R mice showed greater variability than male mice, particularly in the female control group, which resulted in the reduced statistical significance found in the female cohort. In summary, the efficacy observed in terms of disease onset, symptom onset, lifespan, and weight loss demonstrates the sex‐specific differences of a newly synthesized prodrug of EDR, BSZ, with males benefiting to a greater degree in terms of survival, while females had greater magnitudes of disease/symptom onset delay.

We have also recognized limitations in earlier preclinical studies of EDR. Ito and his group investigated the efficacy of EDR and started treatment after symptom onset in a female SOD1‐G93A ALS mouse model. The G93A mice model of ALS is an early onset and fast progressing model, and considering the early onset nature of the disease in these genetic (fALS) animal models, initiating treatment at symptom onset may potentially constrain the window for adequately evaluating the drug's effectiveness in modifying the underlying disease pathology. Second, the investigators used only female mice and did not account for the effect of EDR in demonstrating sex‐specific differences. Mounting evidence from previously published studies shows sex differences in response to drug treatment; therefore, it is important to include sex‐specific analysis in preclinical trials as a means to better translate animal models to human clinical trials [102]. In the present study, we have overcome these limitations to improve the preclinical translational capability of EDR by choosing presymptomatic treatment and including both sexes (male and female) in our treatment and control groups. Moreover, in previous studies, EDR did not demonstrate statistically significant differences in mean extension of survival in female mice compared to the control female mice. The increase was only 1.5 days and 2.2 days in 5 and 15 mg/kg EDR groups, respectively. Compared to these unsatisfactory survival results, BSZ, in the present efficacy study, has demonstrated an increase in survival in both males (16.5 days) and females (13.7 days), with a significant extension of survival in males, and a significant extension in survival when analysing the whole set of animals (i.e., males and females). It is noteworthy to mention that we used a 10 mg/kgbodyweight dose, which was selected based on established PK benchmarks for EDR and the low‐dose neuroprotective in vitro profile of BSZ in NSC‐34 cells. Prior studies by Ito et al. demonstrated dose‐dependent systemic exposure to EDR, with 10 mg/kg body weight achieving a robust C_max_ following I.P. administration. Using this as a reference, we adopted an equivalent dose for BSZ to align with a validated exposure range. Notably, the strong neuroprotective activity of BSZ at lower concentrations suggests the potential to achieve therapeutic efficacy at reduced systemic exposure. One possible reason for increasing the lifespan of both sexes in our study is the reduction of weight loss reported during the progression of ALS. Ito and group did not account for the effect of EDR treatment on weight loss due to ALS progression. Potentially, the effect of EDR treatment on weight improvement was found to be negligible, which is well evident with the negligible effect on the survival of female mice. However, we have recognized weight loss as a clinical prognostic marker for ALS progression [87] and included it in our analysis to see the effect of BSZ in ameliorating disease phenotype by preventing weight loss (ALS‐induced cachexia). The prolonged lifespan observed in both sexes following BSZ treatment may be associated with preservation of body weight in these familial ALS models. This observation could be consistent with a broader attenuation of disease progression and could contribute to improved survival; however, further mechanistic studies are required to clarify this relationship. Further, BSZ has shown a profound effect in ameliorating the disease phenotypes in terms of delaying disease onset and symptom onset while accounting for the sex‐specific differences.

Additionally, the only FDA‐ and EMA‐approved ALS drug known to prolong survival in ALS patients is Riluzole, which extends survival by only 2–3 months. However, in spite of the modest positive clinical trial results in ALS patients, RLZ has never been shown to extend the lifespan to a statistically significant degree in familial mice models of ALS [9]. Riluzole only marginally improves median survival by 3.3% (*p = 0.0525*), when used in the SOD1‐G37R mice model, however, the major limitations of the study were first, the study did not mention the line of G37R mice they used. Second, the study did not account for the treatment response with sex differences, although, they did mention distribution and randomization of the groups with equal sexes [15]. In comparison to the efficacy of RLZ, the newly synthesized BSZ demonstrated significant disease‐modifying effects in the highly aggressive line‐42 SOD1‐G37R mice model while also elucidating sex differences in response to treatment. BSZ significantly prolonged the probability of survival in the whole set of animals with *(p = 0.0174)*. Further, treatment using BSZ increased probability of survival in both males and females, although, only significantly improved the probability of survival in males (*p = 0.0232)* compared to females *(p = 0.2322)*. Using clinically relevant and comprehensive treatment paradigms in the preclinical model, the prodrug of EDR, BSZ, was found to be effective in modifying the disease phenotypes in the line 42 SOD1‐G37R mice model.

Furthermore, to correlate the efficacy of BSZ in the SOD1‐G37R mice model of ALS, we performed untargeted, unbiased discovery‐based global proteomics and phosphoproteomics of lumbar spinal cord samples from end‐stage male mice. One of the limitations of this analysis was the use of lumbar spinal cord samples from the male mice that have reached a humane endpoint. According to our animal protocol approval, we have collected samples of the lumbar spinal cord from both the male and female cohorts who have reached a humane endpoint. However, based on the statistically significant extension of survival (and reduced variability) from the male cohort compared to the female cohort, we initially thought of investigating the global changes of proteome and phosphoproteome in the male cohort. Global proteomics and phosphoproteomics analysis have identified several known and unknown pathways relevant to ALS. The differential expression levels of the proteins and phosphoproteins detected from the end‐stage mice demonstrated different pathological links with neurodegeneration and ALS, as detailed in Tables 2 and 3, respectively. The increased expression (three‐fold upregulation) of Sqstm1/p62 in the treatment group correlates with observed efficacy as Sqstm1/p62 is known to bind with the activator of autophagy marker protein LC3 [103] (essential to autophagosome formation) to facilitate the degradation and removal of ubiquitinated toxic misfolded SOD1 aggregates by autophagy [104, 105]. The decreased expression of Sqstm1/p62 reduces mitophagy in ALS mice [106] leading to a disease phenotype. Furthermore, decreased Sqstm1/p62 expression leads to the accumulation of various neuropathological proteins via negative regulation of autophagy implicated in the huntingtin aggregates [107], Alzheimer's disease [108, 109], Parkinson disease [110], spinal and bulbar muscular atrophy [111], and aging [112]. In addition, Sqstm1/p62 controls various cellular activities, and the decreased expression of Sqstm1/p62 protein leads to mitochondrial dysfunction [113], cellular oxidative stress [114, 115], and defective protein homeostasis [116]. Thus, the Sqstm1/p62 protein acts as a signaling protein for regulating multiple cellular pathways and is a vital protein for removing unwanted toxic proteins in ALS and other neurodegenerative diseases. Reassuringly, the decrease in expression of Sqstm1/p62 observed in the untreated control samples can provide a biochemical rationale as to the decrease in survival and advancement of an ALS disease phenotype observed in the control animals. Another protein, Lrp4, was found to be differentially upregulated in the BSZ‐treated groups. Expression of Lrp4 is associated with the formation of neuromuscular junction [117], modulation of neuronal excitability [118], and astrocytic amyloid beta clearance [119]. Recent studies have detected Lrp4 antibodies in the serum and CSF of ALS patients. However, the link of Lrp4 to ALS pathology is not fully understood [120]. Furthermore, we also detected the increased expression of Cplx2, which is important for neurotransmitter release, in the BSZ‐treated group. Decreased expression of Cplx2 protein is known to be associated with neurological deficits in various neurological disorders, including Alzheimer's disease [121], Parkinson's disease [122], Schizophrenia [123, 124], and Huntington's disease [125]. In addition, recent transcriptome sequencing revealed the downregulation of Cplx2 in ALS patients and mouse models [126, 127]. We also discovered the downregulation of Ca3 in the BSZ treated group, which is a sensitive marker of muscle damage in neurological disorders [128] and aging [129]. Recently, the Ca3 protein was found to be significantly upregulated in the CSF of ALS patients compared to controls in a large discovery cohort, identifying Ca3 as a novel candidate biomarker [130], where increased *Ca3* is associated with ALS. As a result, a decrease in Ca3 found in the treated group can infer decreased neuromuscular degeneration, in comparison to control animal samples. In addition to the known (ALS‐associated) proteomics signatures described above, we unraveled novel increased expression of Gan/KLHL16*, and* Snx13 in the BSZ‐treated group, which are known to be related to neurodegeneration, but unreported in ALS models (to the best of our knowledge). Subtle evidence, from various emerging reports, has shown that the decreased expression of Gan, a cytoskeletal protein that causes impaired autophagy by decreasing ubiquitination and hence the formation of ATG16L1 aggregates [131, 132], is related to, or causes, neurodegeneration. This dysregulation of ATG16L1 causes the deactivation of LC3‐II lipidation, an autophagic protein biomarker, and dysregulation of P62, the main autophagic receptor [132]. Therefore, Gan is recently considered a novel regulator and thus governs the autophagic machinery. In addition, the downregulation of Gan activates the disorganization of intermediate filament (IFs) proteins instigating the accumulation and aggregation of neurofilament (NFs) in the axons of neurons, thus causing axonopathy [133, 134, 135, 136]. Elevation of neurofilaments is the most investigated, and promising, biomarker that can be correlated with the progression of neurodegenerative diseases, including presymptomatic and phenoconversion of ALS. This mounting evidence suggests that Gan is a novel and unique protein regulating the key pathways known in the progression of ALS, thus representing a novel pharmacodynamic target for slowing the progression of ALS [137, 138]. Moreover, following proteomic profiling, we unveiled the novel increased expression of the Snx13 protein, which maintains cellular cholesterol homeostasis by an endolysosomal cholesterol export mechanism [139]. Other reports have shown the pathological link between lipid dysregulation and various neurodegenerative diseases [140], thus upregulation of Snx13 protein could help alleviate the pathology of neurodegeneration.

In our discovery‐based untargeted phosphoproteome analysis of spinal cord samples, Table 3, we interestingly found that recently reported Pgk1 serine 203 (S203) phosphorylation levels were downregulated in the BSZ‐treated group compared to the vehicle‐treated group. The latest studies from Xu and his colleagues delineated the role of S203 phosphorylation in mitigating oxidative stress in spinal cord neurons caused by spinal cord ischemia‐reperfusion (IR) injury (SCIRI). Biochemically, they demonstrated that G‐protein‐coupled receptor (GPCR) kinase 2‐interacting protein‐1 (Git1) is a GTPase‐activating protein that interacts with Pgk1 protein, thereby reducing the S203 site phosphorylation level, hence leading to accumulation of metabolites generated during glycolysis in neurons. Further, these metabolites result in the dimerization of Keap1, thereby, resulting in reducing the degradation of an antioxidant response protein called Nrf2 [141]. The increased expression of Nrf2 maintains the redox homeostasis of the neurons by conferring antioxidant defense to the cells [142]. Thus, mechanistically, decreased expression of S203 Pgk1 phosphorylation is associated with neuroprotective mechanisms via decreased S203 phosphorylation. A large body of evidence has shown the potential role of the antioxidant KEAP1‐NRF2 system as a therapeutic avenue to slow down the progression of ALS via combating oxidative stress [143], however, none of the reports have reported the activation of the KEAP1‐NRF2 system via downregulating S203 phosphorylation of Pgk1. Therefore, BSZ could be a compelling therapeutic agent for slowing down the progression of ALS by modulating the antioxidant KEAP1‐NRF2 pathways.

We also identified novel and known phosphosites of both neurofilament light chain (Nefl) and neurofilament heavy chain (Nefh) proteins in the spinal cord of hSOD1 mice. Neurofilaments are cytoskeleton proteins of long myelinated motor neuron axons. Increased levels of CSF Nefl and CSF phosphorylated (pNefh) [144] and serum NFL levels have been reported to cause abnormal accumulation of both Nefl and Nefh, thus causing slower axonal transport, leading to axonopathy and neuromuscular decline in both SOD1 ALS patients and hSOD1 mouse models [145]. These findings suggest the role of abnormal hyperphosphorylation in SOD1 ALS pathogenesis [145]. In mature myelinated axons, the major sites of phosphorylation in Nefh are Lys‐Ser‐Pro (KSP) repeats in the carboxy‐terminal tail domain, which are essential for maintaining axon caliber, stability, growth, protecting Nefh from proteolysis, and calcium buffering. Additionally, threonine phosphosites in both the mammal and human samples of neurofilaments are reported [146, 147, 148]. Recently, neurofilaments (NFs) have been regarded as the most reliable biomarkers dictating ALS stages, acting both as a valuable tool for prognosis and diagnosis in longitudinal studies during the pathogenesis of ALS [149, 150]. Due to their reliability and translational value, NFs are used as a biomarker for the development of ALS therapeutics, with one such example being the discovery and development of tofersen [151, 152]. With the evolution of NFs as a benchmark universal biomarker in several neurological diseases, it is important to account for emerging techniques to determine multiple sites of phosphorylation of NFs. This will advance our knowledge without bias to see the changes in the global phosphosites in relation to disease progression in the case of ALS. Several reports have investigated the increase in the Nefh and Nefl serine phosphosites in ALS, but threonine phosphosites have never been investigated in Nefh and Nefl. Herein, utilizing an untargeted, unbiased MS discovery‐based phosphoproteomics approach, we have studied and uncovered both serine and threonine phosphosites in the spinal cord of SOD1‐G37R mice. We found that 58 serine/threonine (S/T) sites are downregulated for Nefh and 8 S/T sites are downregulated for Nefl, following treatment with BSZ. Overall, quantification of the S/T phosphorylation sites provides for a general trend of reduction in phosphorylation of Nefh and Nefl, in the BSZ treated group, which also correlates to the general trend of decreased protein expression also detected in our proteomics data for Nefh and Nefl. Overall, this observable trend in downregulation of neurofilaments in BSZ‐treated mice correlates with increased survival, as in general, increases in Nefh/Nefl are seen as being indicative of elevated neurodegeneration. Importantly, phosphorylation of **T72** in Nefh and **T317** in Nefl was significantly reduced in the BSZ‐treated group. To the best of our knowledge, both the T72 and T317 phosphosites have not been reported in the literature. Although our discovery‐based, global untargeted proteomic and phosphoproteomic analyses identified both established and potentially novel pathological pathways, this study was conducted exclusively using end‐stage (humane end point) lumbar spinal cord tissue from male mice. This represents an important limitation, as sex‐dependent differences are recognized in ALS pathophysiology and disease progression. Accordingly, these findings should be interpreted as exploratory and restricted to a male biological context, rather than as definitive evidence of BSZ therapeutic efficacy. Further studies incorporating both male and female cohorts, ideally in a longitudinal framework utilizing tissue from each stage of clinical progression, will be necessary to assess the reproducibility and generalizability of these candidate biomarkers and to determine whether the BSZ‐responsive pathways identified are conserved across sexes or reflect sex‐specific mechanisms.

Further, in our recent efforts to determine the CNS drug profile of BSZ, we conducted in vitro ADME profiling of BSZ, assessing efflux ratio (ER) and LogD at pH 7.4 (Table 4). LogD7.4, the partition coefficient between n‐octanol and buffer at physiological pH, indicates lipophilicity, vital for drug discovery. Additionally, we measured ER through a Caco‐2 permeability assay. The ER compares drug concentration in the brain to that in plasma, reflecting the level of active efflux by Pgp protein across the BBB. First, we determined the bidirectional apparent permeability (Papp) of compounds through Caco‐ 2 monolayers in both the apical‐to‐basolateral (A‐to‐B) and basolateral‐to‐apical (B‐to‐A) directions, and efflux ratio for 1 and 10 µm concentration of BSZ. The efflux ratios (BA/AB) were 0.9 and 1.0, respectively. Studies have shown a strong correlation between low ER and successful brain penetration. A low ER (< 2.5) is a strong positive indicator of CNS penetration and is used in the development of CNS drugs [153]. Further, we evaluated LogD, pH 7.4. A widely accepted parameter for predicting CNS activity is LogD. The widely accepted LogD at pH 7.4 for CNS‐active drugs falls between the preferred lower limit (PL) of 1.2 and the preferred upper limit (PU) of 3.1. The LogD, pH 7.4 of BSZ was found to be 1.56, which is between PL and PU, suitable for crossing the BBB [154].

**TABLE 4:** In Vitro Pharmacokinetic (ADME) Profiling of Borsantrazole (BSZ).

In Vitro ADME Profiling of Borsantrazole (BSZ)	
ADME Properties	BSZ
Caco‐2 Permeability Papp (x10^−6^ cm/sec) for BSZ (1 µM)	**A to B (14.5)** **B to A (12.1)**
Caco‐2 Permeability Assay (Efflux Ratio) for BSZ (1 µM)	**BA/AB (0.9)**
Caco‐2 Permeability Papp (x10^−6^ cm/sec) for BSZ (10 µM)	**A to B (17.3)** **B to A (16.7)**
Caco‐2 Permeability Assay (Efflux Ratio) for BSZ (10 µM)	**BA/AB (1)**
LogD, pH 7.4	**1.56**

BSZ showed favorable CNS drug‐like properties, as indicated by two key pharmacokinetic parameters: ER and LogD at pH 7.4. It exhibited low ER values of 0.9 and 1.0 at 1 and 10 µm, respectively. Suggesting BSZ has lower Pgp efflux liability. Additionally, the LogD at pH 7.4 ranged from 1.2 to 3.1, suggesting BSZ is lipophilic at pH 7.4. These two PK descriptors align well with the typical range for CNS‐active drugs, suggesting that BSZ has CNS drug‐like properties and can cross the BBB to protect motor neurons from oxidative stress, a pathology implicated in the pathophysiology of ALS. We are currently in the process of conducting additional PK ADME property assessments, both in vivo, in mice and rats. The comprehensive PK analysis of BSZ will be available soon to further validate BSZ as a CNS drug‐like molecule.

At this time, we thought it prudent to elaborate upon a key claim of our manuscript and provide preliminary data that demonstrates a) the in vivo conversion of BSZ into EDR and b) the superior drug properties and CNS exposure of BSZ, in direct comparison to EDR. A more detailed experimental description will be available soon, however, we believe it is important to provide evidence of the prodrug ability of BSZ, and its in vivo conversion to EDR, as well as demonstrate the superior CNS drug properties of BSZ in vivo, as inferred by our ADME profiling within our current report. Recently, we were able to obtain some in vivo pharmacokinetics that suggest excellent drug‐like properties for oral delivery, and oral CNS penetration for BSZ. Highlighted in Table 5 are the partition coefficient (Kp) ratios for BSZ and EDR at three time points, 1 h, 4 and 24 h following oral administration of both drugs at 10 mg/kg, the therapeutic dose of BSZ from our ALS mouse model. Kp is the tissue‐to‐plasma partition coefficient for [brain]/ [plasma]. Following oral dosing of EDR, the mean plasma concentrations were 624 ng/mL at 1 h; 47 ng/mL at 4 h and 4.7 ng/mL at 24 h. The mean brain concentrations for EDR were only quantifiable at 4.9 ng/mL at 1 h, giving a Kp ratio of 0.00792 at 1 h, and Not Quantifiable (NQ) for 4 and 24 h. Following oral dosing of BSZ, the mean plasma concentrations were 4020 ng/mL at 1 h; 1266 ng/mL at 4 h and 44 ng/mL at 24 h. The mean brain concentrations for BSZ were found to be 2324 ng/mL at 1 h; 617 ng/mL at 4 h and 13 ng/mL at 24 h, giving a Kp ratio of 0.574 at 1 h, and 0.487 for 4 h and 0.286 24 h. The significant differences in the oral drug properties of EDR and BSZ are magnified when considering that the mean plasma concentration of EDR at 4 h was 47 ng/mL, while the mean plasma concentration of BSZ at 24 h was 44 ng/mL. Although different molecular weights, the molar ratio of the mean plasma [BSZ] at 24 h (1.55x10^−10 ^mol/mL) compared to [EDR] at 4 h (2.70 × 10^−10 ^mol/mL) is 1.55/2.70 = 0.57, meaning we have more than half as much BSZ in plasma at 24 h, compared to EDR at 4 h. This difference is further amplified when considering the molar dose difference between BSZ and EDR, as due to molar weight differences, BSZ is only dosed orally at ∼60% of the molar dose of EDR (10 mg/kg / 174.2 g/mol = 5.74 × 10^−5^ mol/kg EDR versus 10 mg/kg / 284.16 g/mol = 3.52 × 10^−5^ mol/kg BSZ). The mean residence time (MRT), which is the average time one molecule spends in the body and is a more suitable parameter than half‐life to compare compounds, was determined for BSZ and EDR. The MRT for oral dosing of BSZ was 4.49 h with oral bioavailability (%F) of 86%, while MRT of IV dosing of BSZ was 2.89 h. The MRT for oral dosing of EDR was 1.69 h with oral bioavailability (%F) of 76%, while MRT of IV dosing of EDR was 1.40 h. Overall, these results highlight the significant improvement observed in the CNS drug properties of BSZ, compared to EDR, and suggest that the statistically significant efficacy observed in mouse models observed by BSZ could be attributed to improved CNS penetration. Importantly, brain and plasma concentrations were found to range from 7‐13 ng/mL for EDR, following oral BSZ administration, validating the prodrug ability of BSZ, and its in vivo conversion to EDR. Although these values are not very high, the brain concentration of EDR following oral BSZ administration is higher than the brain concentration of EDR following oral EDR administration, 12.6 ng/mL vs 4.94 ng/mL at 1 h, and 7.14 ng/mL vs NQ at 4 h, respectively. These results indicate that the concentrations of EDR found within the brain of our PK mice (CD1 mice), are actually higher when BSZ is administered orally, as compared to when EDR is administered orally at 1 h & 4 h. Additionally, the PK data suggest that the conversion of BSZ to EDR occurs directly in the brain (where mitochondrial activity is high and resulting production of H_2_O_2_ is also high) due to a Kp for EDR that is much higher when BSZ is administered orally, as compared to when EDR is administered orally. Collectively, these results make a strong case that BSZ should progress directly to IND‐enabling studies, due to significant efficacy results, and CNS drug‐like properties that make BSZ a superior orally bioavailable CNS penetrating drug candidate compared to FDA and Health Canada approved EDR.

**TABLE 5:** In Vivo Pharmacokinetics Profiling of BSZ in Comparison to EDR and Prodrug Conversion.

In Vivo Pharmacokinetics and [Brain]/ [Plasma] Partition Coefficient (Kp)			
Time	Kp (PO EDR)	Kp (PO BSZ)	[EDR]_brain_ following PO BSZ admin.
1h	0.00792	0.574	[EDR]_brain_ = 12.6 ng/mL (Kp=1.08)
4h	NQ	0.487	[EDR]_brain_ = 7.14 ng/mL (Kp=0.75)
24h	NQ	0.286	NQ
MRT (PO)	1.69h	4.49h	C_max_ (ng/mL) = 28.7 [EDR]_POBSZ_
MRT (IV)	1.40h	2.89h	t_max_ (h) = 0.194 [EDR]_POBSZ_
%F (PO)	76%	86%	AUC_last_ (ng/mL*h)=43.8 [EDR]_POBSZ_

## 4 Conclusions

Only two drugs have been approved by the FDA for the treatment of ALS that are widely used by patients. More recently, tofersen and ASO got the orphan drug status by the FDA. Riluzole modestly extends survival in ALS patients by only 2–3 months but failed to demonstrate survival efficacy in the familial mice model(s) of ALS. In addition, tofersen faces limitations in patient compliance due to the intrathecal delivery required to bypass the BBB, alongside safety concerns observed in clinical trials. Importantly, its clinical efficacy remains unproven in major trials. Further, EDR efficacy is based on improvement in physical functional outcomes with no survival benefits observed in ALS patients and was repurposed for ALS following development as a free radical scavenger and originally developed to treat acute stroke in Japan. However, EDR was approved based on its efficacy in improving physical functional decline as measured by the ALSFRS‐R score and improved quality of life as measured by the ALS 40‐item assessment questionnaire. Regardless of its ineffectiveness in extending survival in both preclinical mouse models and ALS patients, EDR offers hope to slow down the progression of ALS. Moreover, recent clinical pipeline results are unfortunate and disappointing, as many ALS drug candidates have failed to meet their primary and secondary endpoints. As a result, there is a large unmet need to find new treatment options for ALS. As part of our current early‐phase drug discovery project, we tried to explore and extend the potential of EDR by adding the advantage of a boron scaffold, and developing a triple‐role antioxidant molecule that can combat the pathological free radical H_2_O_2_, an agent that is implicated in the pathophysiology of ALS in both familial SOD1 ALS mice and sporadic ALS patients.

In our progress to find new avenues to modify the severe disease phenotypes in a familial SOD1 mice model of ALS, we have developed a ROS‐responsive and H_2_O_2_‐activatable, boron prodrug of EDR called Borsantrazole (BSZ) that has shown its therapeutic potential in the SOD1‐G37R (line 42) ALS mice model via a proposed triple‐role therapeutic effect. Prodrug BSZ demonstrated an excellent safety profile both in vitro and in vivo. Furthermore, the findings from our early phase drug discovery efforts in a validated familial SOD1‐G37R mice model, indicate that the prodrug BSZ, was able to significantly modify disease phenotypes in terms of delaying the onset of disease, delaying the symptom onset of disease, and extending the lifespan of SOD1‐G37R mice, while also reducing ALS‐induced weight loss/cachexia. Considering that sex is an independent risk factor in the progression of ALS, we have accounted for the treatment response of BSZ in both sexes and found that BSZ has profound effects on modifying disease phenotypes in the SOD1‐G37R mice model. Our approach exploring the discovery‐based differential protein expressions in the lumbar spinal cord of G37R ALS mice unveiled both known and unknown proteins and phosphoproteins as they relate to the pathophysiology of ALS. However, we accept that a limitation of our study was to only analyzed samples from male mice (treated vs untreated) who have reached the humane endpoint in our efficacy study. Intriguingly, the unbiased results of differential protein and phosphoprotein regulation correlated with the efficacy of BSZ and indicated that known ALS biomarkers for proteins (**Cplx2, Lrp4, Sqstm1/p62, and Ca3**) and phosphoproteins (**Nefl and Nefh)** are differentially regulated in the treatment group compared to the control group. Furthermore, these pilot results will motivate us to investigate additional cohorts where we can do longitudinal analysis of brain and spinal cord samples of treated and untreated ALS mice, leading to potential further validation of our current results. The newly discovered unknown (ALS‐associated) proteins **(Gan/KLHL16, Snx13)** and phosphoproteome **(Pgk1)** biomarkers in the ALS mice models will help us understand the overlapping pathophysiological mechanisms across various neurodegenerative diseases. These observations, for the first time in ALS models, have opened the door to further research into the role of BSZ in treating ALS and linking these biomarkers to the common pathogenic mechanism of other neurodegenerative diseases. Furthermore, in vitro ADME–PK characterization reveals a compelling CNS drug‑like profile for BSZ, positioning this boron‑based small molecule as a strong candidate for translational advancement in ALS therapeutics.

These exciting findings, we believe, will provide a foundation for developing a small molecule ALS therapeutic that incorporates boron, through investigating the efficacy of BSZ in slowing down the progression of ALS. Further, this novel prodrug strategy should minimize side effects, while selectively targeting the CNS that is vulnerable to pathological H_2_O_2_ and other free radicals. We anticipate that BSZ could be a novel and effective therapeutic option that targets the oxidative stress‐associated pathology of neurodegeneration and will foster further research into the therapeutic potential of boron in neurodegeneration, such as ALS. The efficacy results of BSZ support our belief that a boron‐based prodrug of EDR could fill the gap of ineffective translation of positive preclinical animal model results to human clinical studies for FDA ALS‐approved drugs. In our current report, we have detailed some limitations of our study, including the need for future pharmacokinetic studies, as well as, to define better cohorts and endpoints to investigate the longitudinal changes in proteomics and phosphoproteomics expression in response to BSZ treatment. In addition, we understand the need to compare the efficacy of the EDR prodrug (BSZ) to EDR in the G37R line 42 model. Moreover, a limitation of this study is the use of a single preclinical model, the SOD1 G37R line 42 humanized ALS model, which is widely employed to investigate familial ALS. Although this model captures key aspects of SOD1‐linked pathology, it may not fully reflect the broader biological heterogeneity of ALS, particularly in sporadic cases. Evaluation of BSZ in additional preclinical models, encompassing both familial and sporadic disease contexts, will be important to further assess the generalizability of these findings. We acknowledged these limitations in the present study, and would like to highlight that we have started moving forward in these areas to address some of these limitations in the future. However, we believe that these initial/pilot drug discovery innovations reported herein, through the development of EDR prodrugs that incorporate boron, and possess an excellent safety profile, significant efficacy, and differential profiling of end‐stage global proteomic and phosphoproteomic expression, will open new avenues for further clinical development, allowing us to contemplate the role of boron in neurodegeneration therapeutics.

## 5 Materials and Methods

**General**: ^1^H and ^13^C nuclear magnetic resonance (NMR) spectra were recorded on a Bruker 400 and 300 MHz spectrometer, Billerica, MA, USA, using DMSO‐d_6_ **(CAS‐2206‐27‐1)**, Acetone‐d_6_ **(CAS‐666‐52‐4)**, Chloroform‐d_3_ **(CAS‐865‐49‐6)**, Methanol‐d_4_ **(CAS‐811‐98‐3)**, Acros organics, Switzerland as solvent with tetramethylsilane (TMS) as an internal standard. Microwave reactions carried out in the CEM explorer hybrid‐12 microwave reactor. n‐Butyllithium, 2.5 m solution in hexanes, Acros organics **(CAS‐109‐72‐8)** product of Germany; 2‐Isopropoxy‐4,4,5,5‐tetramethyl‐1,3,2‐dioxaborolane **(CAS‐61676‐62‐8)** purchased from Sigma‐Aldrich, St. Louis, MO, USA; 3‐Methyl‐1‐phenyl‐1H‐pyrazole (Number: AK139802) was purchased from Ark Pharm, Inc., Arlington Heights, IL, USA. Hydrogen peroxide (H_2_O_2_) **(CAS‐7722‐84‐1)** was purchased from Sigma‐Aldrich, St. Louis, MO, USA. Lactic acid (CAS‐7732‐18‐5) was purchased from Acros Organics. The reactions were monitored by TLC (Sigma, Silica gel 60 F_254_). The crude reaction mixture was purified with silica gel column chromatography on a CombiFlash Rf 200 purification system, Teledyne Isco, USA. Organic solvents were ordered from BDH, VWR Analytical unless specified otherwise. All chemicals were used without further purification unless stated otherwise.

### 5.1 Scheme I for the Synthesis of Edaravone Prodrug BSZ (*N*‐arylated Pyrazole Boronic Acid Pinacol Ester) via a Two‐Step Synthetic Procedure

The synthetic route for the synthesis of BSZ involves the synthesis of *N*‐arylated pyrazole boronic acid pinacol ester from the commercially available *N*‐arylated substituted pyrazole starting material via a two‐step synthetic procedure. The first step (**A)** involves lithiation at the C‐5 position of *N*‐arylated substituted pyrazole with N‐butyl lithium (n‐BuLi), by a directed Ortho metalation (DOM) mechanism. The second step (**B)** involves the electrophilic substitution of Lithium at C‐5 position with isopropoxy 4,4,5,5‐tetramethyl‐1,3,2‐dioxaborolane (PINBOP). This is followed by an acidic workup which yielded *N*‐arylated substituted pyrazole boronic acid pinacol ester as our first proposed boron‐based EDR prodrug (BSZ, B5‐EDR) Figure 17.

**FIGURE 17:**
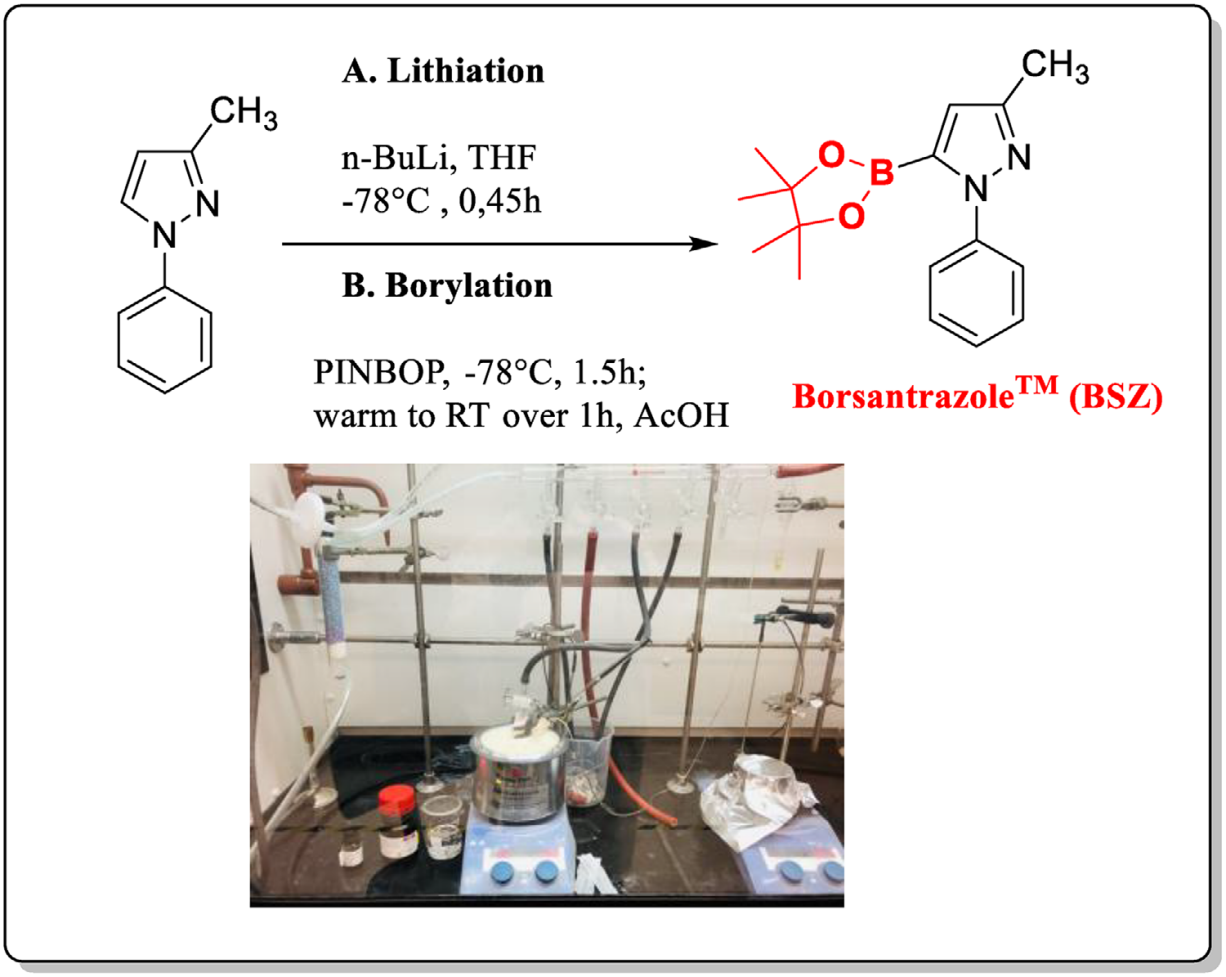
Scheme I for synthesizing N‐arylated pyrazole boronic acid pinacol ester Borsantrazole (BSZ) from the commercially available N‐arylated substituted pyrazole.

### 5.2 Methodology for Synthesis of Edaravone Prodrug (BSZ) 3‐methyl‐5‐(4,4,5,5‐tetramethyl‐1,3,2‐dioxaborolan‐2‐yl)‐1‐phenyl1H‐pyrazole

Synthesis of Borsantrazole (BSZ) was carried out according to the reported procedure [155] with slight modifications, **scheme I,** Figure 17. n‐Butyllithium (2.5 m in hexane, 1.5 cm^3^, 3.793 mmol) was added dropwise to a solution of *N*‐arylated substituted pyrazole (500 mg, 0.471cm^3^, 3.161 mmol) in anhydrous THF (22 cm^3^) at −78°C under argon. The reaction mixture was stirred for 45 min at −78°C. 2‐Isopropoxy‐4,4,5,5‐tetramethyl1,3,2‐dioxaborolane (646.93 mg, 709.5 µL, 3.477 mmol) was added dropwise to the reaction mixture at −78°C and the mixture was stirred for 1.5 h. The mixture was warmed to room temperature over 1 h, and glacial acetic acid (208.79 mg, 199 µL, 3.477 mmol) was added. The mixture was filtered through a Celite pad, which was washed with EtOAc (100 cm^3^). The organic solvent was removed in vacuo to afford a crude product. The expected product confirmation was done by TLC (20% EtOAc/Hex). The crude product was then purified with silica gel column chromatography on a CombiFlash Rf 200 purification system, Teledyne Isco, USA, with ethyl acetate and hexane from 0% to 10%. Residual solvent was evaporated under vacuum to a final light brown crystalline solid product (815 mg, 90%).

### 5.3 Microwave Synthesis of Edaravone from its Prodrug (BSZ) 3‐methyl‐5‐(4,4,5,5‐tetramethyl‐1,3,2‐dioxaborolan‐2‐yl)‐1‐phenyl1H‐pyrazole

The synthesis of Edaravone from its prodrug BSZ is influenced by the bio‐inspired catalyst‐free reported procedure [156]. Reactions carried out in microwave irradiations **(300Psi, premixed 15 sec, 200watt)** were performed on a **0.5** mmol scale. N‐phenyl‐3‐methylpyrazole boronic ester **(1.0 equiv.)** and diethyl ether **(3.5 mL in excess)** were added to a 10 mL microwave vial equipped with a stir bar, followed by the addition of lactic acid **(10 equiv.)** and hydrogen peroxide **(30%w/w, 1.1 equiv.)**. The mixture was then capped and placed in the microwave reactor and heated to **50°C** for **10 **min. After completion of the reaction, the reaction was allowed to cool at RT. The vial was removed from the microwave and the small aliquot was taken for TLC analysis. A single new spot corresponding to Edaravone was observed under U.V. in TLC. The reaction mixture was diluted with water (5 mL) and extracted with **(10 mL ethyl acetate and 2 mL hexane)**. The organic extract was dried in vacuo and subjected to flash chromatography (EtOAc/hexanes) to afford the desired product. The purified solution obtained after flash chromatography was dried in vacuo to obtain edaravone in the form of clear oil.

**Recrystallization**: The clear semi‐viscous oil was cooled in an ice bath, and a small amount of diethyl ether dropwise was added (0.5–1 mL). The resulting oily solution was stirred vigorously in a water bath sonicator for 5 min. The white crystalline solid reappeared during sonication. The resulting white crystalline solid was dried completely under vacuum to obtain pure Edaravone. The purified product was sealed with paraffin in the glass vial and stored under 4°C. Compound NS‐1‐10 (120 mg, 84.50%, see product chromatogram NS‐1‐10 purified in supplemental for reference) was prepared by this method and imparted 1D‐proton NMR data similar to those reported in the literature for Edaravone.

**TLC Observations**: TLC observation of Crude reaction mixture. The TLC was carried out in the solvent system of 50% ethyl acetate and hexane. Rf observed for Edaravone, crude reaction mixture, and the isolated product was found to be equal i.e. Rf = 0.55.

### 5.4 Methodology for Amplex Red Assay to Determine the Hydrogen Peroxide (H_2_O_2_)‐Scavenging Ability of EDR and BSZ

The assay was performed using Amplex Red Hydrogen Peroxide/Peroxidase Assay Kit Catalog number: A22188 Invitrogen. H_2_O_2_ solution (88 mm) was mixed with different working solutions of Edaravone or BSZ and incubated at RT for 30 min. After 30 min, the reaction was started by adding Amplex Red (100 µm) in a final 100 µL of reaction volume and incubated at RT for 30 mins. The final concentration of Amplex Red reagent was (50 µm) in the HRP working solutions. Note, that the reaction was carried out in the absence of light. The H_2_O_2_ scavenging ability of the compounds was measured by fluorometric assay at (em/ex = 590 nm/530 nm). In the presence of Horseradish peroxidase (HRP) an Iron (Fe)‐metalloenzyme, the Amplex Red reagent reacts with H_2_O_2_ in a 1:1 stochiometric ratio to produce the red fluorescent oxidation product, resorufin.

### 5.5 Methodology for Neurotoxicity/Cell Viability Analysis of EDR Prodrug BSZ in Primary Cortical Neuronal Cells (PCNC)

Primary neurons exhibit various unique features that cannot be adequately mimicked by cell lines. Primary cortical neurons were collected from the fetus of CD1 mice (RRID:IMSR_CRL:022) at gestational days 17–18. The most important feature of primary embryonic rodent cultures is that the culture is generally prepared from embryonic day 17–18 (E17‐E18) brain; equivalent to the third trimester of human pregnancy. Therefore, these rodent brains contain many more post‐mitotic neurons than the human fetal neuron cultures with several advantages. Briefly, the cerebral cortex was isolated from the fetus of the mouse at about 17–18 days gestation. After meninges and blood vessels were removed, the tissue was digested with trypsin for about 15 min at about 37°C, then incubated with DNase for about another 15 min at about 37°C and terminated with DMEM plus about 10% fetal bovine serum. After the supernatant was removed, the tissue was added with about 5 mL neurobasal medium (Life Technologies Inc) and triturated by gently pipetting. Neuronal cells contained in the supernatant were transferred to poly‐D‐lysine‐coated plastic culture plates, adjusted to approximately 106 cells/mL, and cultured in neurobasal medium with GS21 supplements (Sigma–Aldrich Canada), 1X GlutaMax (Life Technologies Inc,) and about 1% penicillin–streptomycin at about 37°C under about 5% CO2. About 100k cells/100 µL/well were seeded in 96 well plates for 2Div. At 2Div, the neurons were treated with different concentrations of EDR (1, 10, and 25 µm) and EDR analogues BSZ, (1, 10, and 25 µm) and incubated until 8Div. On the eighth Div of neurons, about 10 µL of WST reagent was added to each well and incubated for about 2 h. After about 2 h of incubation, the absorbance of soluble colored formazan dye was measured calorimetrically at about 450 nm using a microplate reader (Biotek instruments) with the background control as a blank. The cell viability was determined by comparing the absorbance of compound‐treated cells to that of control cells. Data are representative of two independent experiments, with each measurement or dose tested six times.

### 5.6 Methodology for Neurotoxicity/Cell Viability Analysis of EDR Prodrug BSZ in Neuroblastoma‐Spinal Cord Hybrid (NSC‐34) Cells

The NSC‐34 cell line (RRID:CVCL_D356). Vendor (Cedarlane: CLU140)) also called the neuroblastoma‐spinal cord (NSC) hybrids cell line was first developed by Cashman et al. by fusing the aminopterin‐sensitive neuroblastoma N18TG2 with motor neuron‐enriched embryonic day 12–14 spinal cord cells. These mouse‐mouse neural hybrid cell line hybrids have characteristics of adherent multipolar cells with long neurites that resemble motor neurons and display additional key features of motor neurons like generation of action potentials, expression of neurofilament triplet proteins, and acetylcholine synthesis, storage, and release. Several studies used NSC34 mouse motor neuron cells extensively in drug screening studies for ALS and in the study of biochemical pathways during neurodegeneration. Therefore, these NSC34 mouse motor neuron cells act as a suitable model to study motor neuronal pathophysiology in neurodegenerative diseases like ALS [99]

Briefly, NSC‐34 cells with about 20k cells per well in 96 well plates were cultured in a complete medium consisting of high glucose Dulbecco's modified eagle medium (DMEM) (Thermo Fisher Scientific) supplemented with about 10% US‐origin fetal bovine serum (Thermo Fisher Scientific), GlutaMAX‐1, about 200 mm (100X) (Thermo Fisher Scientific), about 1% 100 mm Sodium pyruvate and about 1% 10 000 U/mL penicillin‐streptomycin solution (Thermo Fisher Scientific) for about 20 h to reach confluency of about 70%–80%. All the cells were cultured in an incubator with about 5% of CO_2_ at about 37°C. About 20 k cells/100 µL/well were seeded in 96 well plates for about 20 h in triplicates. Cells were treated with different concentrations of BSZ (and incubated for about 20 h. About 10 µL of WST reagent was added to each well and incubated for about 2.5 h. After about 2.5 h of incubation, the absorbance of soluble colored formazan dye was measured calorimetrically at about 450 nm using a microplate reader (Biotek instruments) with the background control as a blank. The cell viability was determined by comparing the absorbance of compounds‐treated cells to that of control cells. All the experiments were repeated at least three times and measurements were run in triplicates.

### 5.7 Methodology for Neuroprotective Effect/Cell Viability Analysis of EDR Prodrug BSZ in Neuroblastoma‐Spinal Cord Hybrid (NSC‐34) Cells

Briefly, NSC‐34 cells with about 20 k cells per well in 96 well plates were cultured in a complete medium consisting of high glucose Dulbecco's modified eagle medium (DMEM) (Thermo Fisher Scientific) supplemented with about 10% US‐origin fetal bovine serum (Thermo Fisher Scientific), GlutaMAX‐1, about 200 mm (100X) (Thermo Fisher Scientific), about 1% 100 mm Sodium pyruvate and about 1% 10 000 U/mL penicillin‐streptomycin solution (Thermo Fisher Scientific) for about 20 h to reach confluency of about 70%—about 75%. All the cells were cultured in an incubator with about 5% of CO_2_ at about 37°C. About 20 k cells/100 µL/well were seeded in 96‐well plates for about 20 h in triplicates. After about 20 h, the media were changed, and the cells were pre‐treated or prophylactically treated with different doses of EDR analogue (BSZ) and EDR for about 1 h. After about 1 h, the cells were treated with 250 µm of H_2_O_2_ for about 2 h. After a total period of about 3 h, about 10 µL of WST‐8 reagent was added in each well, and the absorbance readings were recorded after about 2.5 h at about 450 nm. All the experiments were repeated at least three times, and measurements were run in triplicates.

#### 5.7.1 Animal Models

To study the safety profile of small organic molecule BSZ, both in terms of evaluation of acute toxicity (single dose) and chronic toxicity (120 doses), we used naïve wild‐type SOD1 mice (non‐transgenics) according to the principles of preclinical toxicity determination of molecule by most of the studies. We used the commonly used C57BL/6J strain of mice (RRID: IMSR_JAX:000664) to study the toxicity effects of BSZ both acutely and chronically. Many SOD1 mouse models have been created, out of which the most studied and commonly used SOD1 mouse models to study ALS are SOD1‐G93A and SOD1‐G37R. These humanized SOD1 mice develop the clinical genotype, phenotype, and pathology of ALS as observed in humans [157].

We have used human (h) SOD1‐G37R line 42 mice (RRID:IMSR_JAX:008342) to evaluate the efficacy of EDR prodrug BSZ. The hSOD1‐G37R line 42 transgenic strain was designed with a mutant human SOD1 gene (harboring a single amino acid substitution of glycine to arginine at codon 37) driven by its endogenous human promoter. Wong et al. assessed the survival of the line 42 strain on mixed genetic background and noted that death occurred around 3.5‐4 months of age. However, the original allele has been backcrossed to C57BL/6J and made fully congenic, with increased survival of line‐42 strain to (6–7 months or 180‐210 days). Line 42 expresses 14.5‐fold elevated levels of total SOD1 activity relative to control in the spinal cord. This SOD1‐G37R line 42 is most similar to the G93A‐high copy (HC) line with respect to disease onset age (∼3.5–4 months or 105–120 days) and survival (6–7 months). With G93A‐HC, the disease onset age (4 months) and survival age (∼6 months). The other G37R mouse line 29 has an increased survival age (∼17 months) with prolonged disease onset age (∼10 months) [158]. The clinical phenotypes that resemble the clinical phenotypes of humans include fine axial tremors, asymmetric weakness of limbs, poor grooming, rough coat, muscle wasting around the flanks, kyphosis, hind limb paralysis, and abnormal splay of toes [51]. The SOD1‐G37R line 42 used in this study is highly aggressive and demonstrates fast disease progression with an early disease onset and symptoms with muscle weakness according to the original investigator and most of the studies [51].

#### 5.7.2 Mice and Tissue Preparation

Transgenic mice carrying human G37R mutant SOD1 [B6.Cg Tg(SOD1*G37R)42Dpr/J] were obtained from the Jackson Laboratory (Bar Harbor, ME, USA, RRID:IMSR_JAX:008342). These mice were crossed with female mice with a C57BL/6 background for at least four generations. Colonies are maintained in the Central Animal Care Services (CACS), University of Manitoba, in an environment free of pathogens. The mice were used in accordance with the Guide of Care and Use of Experimental Animals of the Canadian Council on Animal Care. Transgenic offspring were genotyped by PCR of DNA obtained from ear biopsies, see below, using a protocol provided by the Jackson Laboratory. All animal experiments for this study followed protocol 21‐014(AC11693), approved by CACS & Animal Care Committee at the University of Manitoba.

#### 5.7.3 DNA Isolation and Genotyping

A total volume of 300 𝜇L of TNES buffer (1 m Tris, pH 8.5, 0.5 m EDTA, about 10% SDS, 5 m NaCl, and distilled water) supplemented with 20 mg/mL of Proteinase K (25530049, Fisher), was used to lyse ear samples overnight at 55°C. After complete lysis, the mixture was combined with an equal volume of phenol and chloroform (1:1) and mixed well. After centrifugation at 14 500 rpm for 15 min, the samples were cleared of debris, and the DNA‐rich supernatant was collected. The DNA was then precipitated by adding 200 µL of supernatant with 200 µL of 95% ethanol (stored at −20°C). The mixture was kept at 4°C for 30 min. The DNA was then pelleted by centrifuging at 14 500 rpm at 4°C for 10 min. The pellet was then rinsed with 70% ethanol and centrifuged at 14 500 rpm for 10 min, 4°C to remove residual solvent. Any remaining ethanol was evaporated in the ventilated hood over an hour. After the tubes were dried completely, 30 µL of nuclease‐free water, not DEPC‐Treated (Fisher, AM9939) was added to the DNA, and the mixture was heated for 55°C for 10 min. The genotype determination was applied via the Polymerase Chain Reaction (PCR) with a PCR reaction mixture for genotyping (23 µL/ well) was prepared over ice by mixing Phusion High‐Fidelity PCR kit (M0530S, New England Biolabs Inc.). The PCR reaction mixture was made by adding 14.75 µL of nuclease‐free water, 5 µL 5x Phusion HF reaction buffer from the PCR kit, about 0.5 µL of about 10 mM dNTPs (New England Biolabs, N0447S), 1.25 µL of about 10 µm forward primer (5’‐CATCAGCCCTAATCCATCTGA‐3’) (Fisher, 10336022), about 1.25 µL of about 10 µM reverse primer (5’‐CGCGACTAACAATCAAAGTGA‐3’) (Fisher, 10336022), and finally about 0.25 µL of Phusion HF DNA polymerase (added near the freezer) kept at −20°C from the PCR kit, lastly, 2 µL of DNA extracted sample was added into each well. PCR program for the above PCR reaction includes initial denaturation at 98°C for 30 s, followed by 30 cycles of 10 s at 98°C, then 30 s at 54°C, and 30 s at 72°C. A final step at 72°C for 7 min terminated the amplification. The resulting PCR products were stained with GelRed Prestatin (Cedarlane, 41011, BT) and separated on a 1% agarose gel. The gel was visualized using GeneSys imager software on a G: BOX imager (Syngene, UK).

### 5.8 Group Assignment, Drug Formulation, Storage, and Administration of Compound Group Assignment for Determination of Acute Toxicity in Wild‐Type SOD1 Mice

The Wild‐type SOD1 mice were randomly assigned to 2 groups: For the acute toxicity experiments, a total of 6 animals per group (3 male and 3 female) were used in the study. **Group 1**: Sham treatment 1 (1:20 DMSO/PBS) and **Group 2**: BSZ (10 mg/kg body weight). According to an acute toxicity study requirement, 6–10 animals should be used to evaluate the effect of a substance. However, as the new analogues are structurally similar to Edaravone, having a single‐dose safety profile of 450 mg/kg, the mortality of the Edaravone analogues at a dose of 10 mg/kg body weight/day was not expected to cause any toxicity. Considering the above literature evidence, for the present acute study, the minimum number of animals was 6 per group. Morphological alterations, histological changes, and mean body weight assessments for the whole set of animals, for both the Wild‐type SOD1 control groups (N = 6; 1:20, DMSO: PBS) and Wild‐type SOD1 treated groups (N = 6; BSZ; 10 mg/kg body weight) were assessed during the longitudinal 14‐day acute toxicity assessment, by giving a single IP injection to the mouse starting at 2 months of age (see further detail in (1.8) below). Thus, a total of 12 Wild‐type SOD1 mice were used.

### 5.9 Group assignment, Drug Formulation, Storage, and Administration of Compound for Determination of Chronic Toxicity in Wild‐Type SOD1 Mice

The Wild‐type SOD1 mice were randomly assigned to two groups. For the chronic toxicity experiments, a total of 6 animals per group (3 male and 3 female) were used in the study. **Group 1**: Sham treatment 1 (1:20 DMSO/PBS) and **Group 2**: BSZ (10 mg/kg body weight). Morphological alterations, histological changes, and mean body weight assessments for the whole set of animals, for both the Wild‐type SOD1 mice control groups (N = 5; 1:20, DMSO: PBS) and Wild‐type SOD1 mice treated groups (N = 6; BSZ; 10 mg/kg body weight) were assessed during the 120‐day chronic toxicity assessment, by giving daily of 120 IP injection to the mouse starting at 3 months of age. Thus, a total of 12 Wild‐type SOD1 mice were used. Only one male mouse from group 1 (control) was terminated unexpectedly, which was likely due to an accidental error in injection (discussed with Central Animal Care Services, veterinarians, and staff), allowing 11 mice to finish the study. IP injections may be difficult because of the aggressiveness of mice (particularly the male mice, during physical restraint). In total, 1320 (11 X 120) daily IP injections were completed for the full chronic toxicity assessment (see further detail in (1.8) below).

### 5.10 Drug Formulation, Storage, and Route of Administration Determination for the Acute and Chronic Toxicity Evaluation in the Wild‐Type SOD1 Mice

For intraperitoneal administration, about 1:20 of DMSO/PBS, at a pH of about 7.4 was used for the suspension of BSZ test substance. A volume of about 400 µL from the final reconstituted solution was aliquoted and stored at about −80°C, until further use. For acute toxicity experiments, a single dose of about 10 mg/kg body weight of BSZ was intraperitoneally injected into the group 2, wild‐type mice model at the age of 2 months. The wild‐type mice in group 1 received an injection of an equal volume of (about 1:20; DMSO: PBS) instead. For chronic toxicity experiments, 120 daily doses of about 10 mg/kg bodyweight of BSZ were intraperitoneally injected into the group 2, WG37R mice model at the age of 3 months for 120 days, until the age of 7 months. The wild‐type mice in group 2 received an injection of an equal volume of (about 1:20; DMSO: PBS) instead.

#### 5.10.1 Hematoxylin and Eosin (H&E) Staining and Microscopy

Wild‐type (WT) SOD1 mice received a single dose (about 0.2 mL) IP injection of Sham suspended in (about 1:20; DMSO: PBS) and a single dose of about 10 mg/kg body weight (about 0.2 mL) IP injection of BSZ suspended in about 1:20; DMSO: PBS at the age of 2 months. The mice were observed longitudinally for 2 weeks. At the experimental endpoint, the mice were first deeply anesthetized with a mixture of about 20% v/v isoflurane/propylene glycol (about 1 mL of the mixture per about 500 mL of bell jar space, University of Manitoba animal care SOP A003), and than exsanguination was done by cutting the animal's right atrium followed by an intracardiac perfusion with about 0.9% NaCl. During perfusion, a syringe barrel nosecone was used for prolonged anesthesia. Cardiac perfusion was followed by perfusion with about 4% paraformaldehyde for histology (H&E staining) analysis. Mouse tissues (brain, heart, spinal cord, kidney, liver, muscle, lung, and spleen) were fixed for about 48 h in about 4% buffered formalin at about 4°C. Only the spinal cord was processed after about 24 h to remove the spine and again fixed for about another 24 h. Samples were processed and embedded into paraffin blocks at the Histomorphology and Ultrastructural Imaging platform, Dept. of Human Anatomy and Cell Science, University of Manitoba. Briefly, the embedded tissues were cut into about 5 µm thickness, mounted on super frost plus slides, and dried overnight at about 37°C. Slides were deparaffinized in about 2 changes of xylene and rehydrated in descending alcohols (about 2 changes of about 100% Ethanol and about 2 changes of about 95% Ethanol) and tap water. Slides were stained with Harris Haematoxylin followed by differentiation with acid alcohol. After rinsing in tap water, saturated lithium carbonate was used for Blueing the nucleus. After that, the slides were rinsed in tap water and counter‐stained with eosin. Following eosin staining, the slides were dehydrated using ascending alcohols, cleared with xylenes, and mounted coverslips on sections with paramount. The mounted slides were then visualized, and the images were taken using a Zeiss Imager M2 microscope using ZEN 3 Pro software with camera AxioCam HRc—color. Similar to the acute toxicity experiments, hematoxylin and eosin (H&E) staining and microscopy experiments were done for the animals assigned to the chronic toxicity group.

Group assignment for evaluation of therapeutic efficacy in terms of delaying the disease onset, symptom onset, extending survival, and decreasing weight loss (cachexia) of EDR prodrug BSZ, in direct comparison to control/sham group in the human SOD1‐G37R mouse model of ALS.

For these efficacy experiments, a set of experiments was carried out having two groups of mice: **Group 1**: Sham treatment (1:20 DMSO/PBS); and **Group 2**: BSZ treatment (1:20 DMSO/PBS) (10 mg/kg body weight/day). For these experiments, age‐matched Het human SOD1‐G37R (Line 42) mice were administered vehicle (1:20, DMSO: PBS) or treatment (BSZ; 10 mg/kg body weight), daily, starting pre‐symptomatically at the age of 90 days until the age of 210 days, and monitored longitudinally for several significant humane point indicators/end stage including, (a) Mouse unable to right itself in 15 sec (b) 25% loss of weight on the highest recorded weight (c) Full paralysis of one or more hind limbs (d) Loss of bladder functions (e) Crusty eyes/loss of vision and/or (f) Penile prolapse. Weight loss or ALS‐induced cachexia due to disease progression is an independent predictor of survival and disease progression. We have evaluated the weight loss based on the highest recorded weight minus weight at the humane endpoint/end stage [87].

#### 5.10.2 Definitions and Criteria for Determining Disease Onset and Symptom Onset

The disease onset and symptom onset in SOD1‐G37R ALS mice were evaluated in the current study based on the following criteria, which are being used by numerous studies conducted in ALS mice models. The definitions of disease course and progression analysis are illustrated in Figure 18.

**FIGURE 18:**
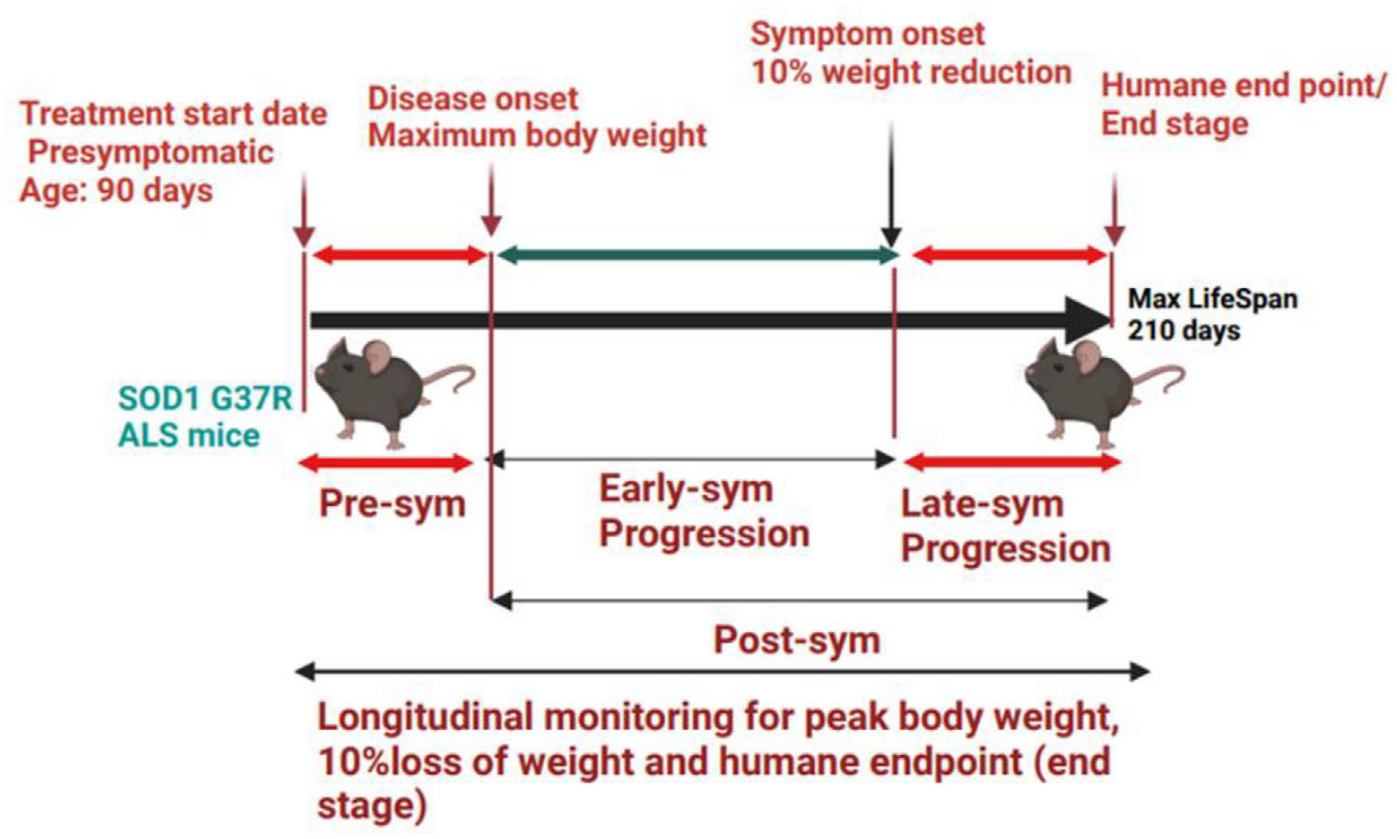
Diagram outlining the definition of pathological stages of the SOD1‐G37R ALS mice mouse model of ALS.

**Criteria for Disease Onset**: Disease onset is retrospectively defined as the age at which the mouse reaches peak weight, well before denervation‐induced muscle atrophy and weight loss [159].

**Criteria for Symptom Onset**: Symptom onset or early symptomatic progression stage is defined as the age to reach 10% body weight loss based on the highest or peak recorded weight with muscle weakness. The loss of 10% of body weight is often accompanied by the appearance of symptoms of muscle weakness [160]. Symptom onset is the early symptomatic disease progression stage called early symptomatic stage (Early‐sym).

#### 5.10.3 Injection Sites and Determination of Sample Size for Efficacy Studies of BSZ

Injection sites were alternated between the right and left sides of the mice to reduce pain/inflammation. In this efficacy study of EDR prodrug BSZ, a total of 12 animals per group (6 male and 6 female) have been used. Thus, a total of 24 SOD1‐G37R mice were used.

#### 5.10.4 The Rationale for Presymptomatic Treatment Start Date

The treatment of EDR prodrug BSZ in the efficacy studies was initiated well before the disease onset i.e., started pre‐symptomatically. According to the original investigator Wong et al., highly expressed SOD1‐G37R line 42 mice, a familial model of ALS, demonstrate the onset of motor neurodegeneration at the age of (3.5–4 months or 105–120 days). At the earliest age of (15 weeks or 105 days), progressive neuropathological abnormalities associated with loss of motor neurons in the spinal cord and brain stem were observed, which are associated with ventral root exit swelling and vacuolization. According to several studies, it is well known that both forms of ALS i.e., sALS and fALS are clinically and pathologically indistinguishable, further studies have shown the shared genetics risk in both forms of ALS. Further, evidence from studies in ALS mice models and human forms of ALS demonstrates that the neurodegenerative process in ALS starts before the manifestations of ALS clinical symptoms or disease onset, culminating in a complex process and further leading to an aggressive and fatal form of disease progression. Therefore, better outcomes of therapeutic intervention in all forms of ALS could be achieved if we initiate a treatment presymptomatically to slow the fundamental root cause of neurodegeneration in the disease course. In addition, presymptomatic treatment gives an opportunity to delay the phenoconversion in ALS (i.e., the transition between pre‐symptomatic and clinical symptomatic phases of the disease). There are several advantages of using early intervention to slow down the progression of highly complex ALS. First, highly aggressive forms of neurodegeneration later, after the onset of the disease could mitigate the therapeutic potential of the drug therefore, initiating the intervention presymptomatically in early‐phase drug discovery could be advantageous, second, rescuing the postmitotic neurons is more pragmatic than the regeneration of dead or diseased neurons, third, it is preferred to start early intervention to slow the disease progression, when the disability is minimal and lastly, high levels of phosphorylated neurofilaments (pNefl) levels in the serum of presymptomatic ALS patients and ALS mice models demonstrates that the neurodegenerative process is active at the clinically presymptomatic stage [138]. This cumulating evidence endorses and supports the initiation of the BSZ presymptomatically in the current SOD1‐G37R mice model to successfully translate the results from preclinical to human studies in the future.

Overall, a total of 48 mice participated in this early drug discovery phase, based on approved #protocol 21‐014(AC11693) CACS, University of Manitoba. To evaluate the safety of BSZ (in terms of both acute and chronic) and efficacy of BSZ (in terms of delaying disease onset, extending life span, and reducing weight loss). A total of (12 wildtype SOD1 mice) were a part of acute toxicity studies, a total of (12 wild‐type SOD1 mice) were a part of chronic toxicity experiments, and a total of (24 human SOD1 G37R mice) were a part of efficacy studies.

### 5.11 Proteomics on Human Mutant G37R Mouse Lumbar Spinal Cord Tissue Samples

#### 5.11.1 Tissue Sample Preparation

For homogenization, a lysis buffer comprised of 4% SDS and 1x Halt Phosphatase Inhibitor Cocktail (Thermo Scientific, cat no. 78426) in 100 mm TRIS (pH 8.5) was added to each sample, and a probe sonicator was used. Protein concentrations of the lysates were determined using the Thermo Scientific PierceTM Detergent Compatible Bradford Assay Kit (product number. 23246) as per the manufacturer protocol. Per sample, 300‐ug aliquots were further processed, and volumes were adjusted to 30 µL using lysis buffer.

#### 5.11.2 Proteolytic Digestion

Lysates were reduced with 10 mm dithiothreitol (DTT) for 30 min at 57°C followed by alkylation using 50 mm iodoacetamide (IAA) for 45 min in the dark at room temperature. Samples were processed using single‐pot solid‐phase‐enhanced sample preparation (SP3) [161]. Thus, two types of carboxylate‐modified SeraMag Speed beads (GE Life Sciences) were combined 1:1 (v/v), rinsed, and reconstituted in water at a concentration of 20 µg/µL. Next, 30 µL of the bead mix was added to each lysate, and samples were adjusted to pH 7.0 using TRIS buffer. To promote protein binding, acetonitrile (ACN) was added to a final concentration of 70% (v/v), and samples were incubated at room temperature on a tube rotator for 18 min. Subsequently, beads were immobilized on a magnetic rack for 1 min. The supernatant was discarded, and the pellet was rinsed twice with 200 µL of 70% ethanol and once with 180 µL of 100% ACM while on the magnetic rack. Rinsed beads were resuspended in digestion buffer, 120 µL of 50 mm TRIS buffer (pH 8.5) supplemented with trypsin (sequencing grade, Promega) at an enzyme‐to‐protein ratio of 1:25 (w/w). Samples were incubated for 16 h at 37°C. Supernatants were collected in clean tubes. To increase recovery, the beads were removed from the rack and sonicated in a bath sonicator with 50 µL of digestion buffer without trypsin. Supernatants were added to their respective tubes from the previous step. Next, 170 µL of 1% trifluoracetic acid (TFA) was added to samples, and the generated peptides were desalted on Waters C18 100 mg Cartridges (Sep‐pac, part no. WAT023590). The desalted samples were dried in a speed vac and resuspended in 0.1% formic acid (FA).

#### 5.11.3 Phosphopeptide Enrichment

Immobilized metal ion affinity chromatography (IMAC)‐based phosphopeptide enrichment was performed with aliquots of 145 and 105 µg of peptide for brain and spinal cord samples, respectively, using the High Select Phosphopeptide Enrichment Kit (Thermo Scientific, cat no. A32992) kit as per manufacturer protocols. Phosphopeptide eluates were dried in a speedvac and resuspended in 15 µL of 0.2% FA.

#### 5.11.4 LC‐MS/MS Analysis Spinal Cord Proteome

Per sample, an equivalent of 1.25 µg of peptide was analyzed by nanoflow LC‐MS/MS using an Easy‐nLC 1200 system coupled to an Orbitrap Exploris 480 with FAIMS (both Thermo Fisher Scientific). Samples were separated on a self‐packed C18 analytical column (Luna C18(2), 3 µm particle, 100 µm ID × 30 cm, Phenomenex, Torrance, CA) using water with 0.1% FA (mobile phase A) and 80% ACN with 0.1% FA (mobile phase B) using a binary gradient starting with 5% B for 3 min, 5%–7% B over 2 min, 7%–25% B over 87 min, 25%–60% B over 15 min, 60%–95% B over 1 min, with final elution of 95% B for 15 min at a flow rate of 300 nL/min. A standard nitrogen flow of 4.1 L/min is used for all experiments, with user gas kept at 0 L/min. MS acquisition was conducted in data‐dependent acquisition mode (DDA) with a cycle time of 1.5 s for both CV −50 and −70. Full MS scans were acquired at a resolution of 1 20 000 from 380 to 1500 m/z (AGC 300%, 50 ms max injection time). Precursor ions with charge states between +2 and +5 were isolated with a m/z 1.6 window and fragmented with a normalized collision energy of 30%. MS/MS spectra were acquired at a resolution of 15 000 (AGC 100%, automated max injection time). Dynamic exclusion was set to 25 s.

#### 5.11.5 LC‐MS/MS Analysis Spinal Cord Phosphoproteome

Per sample, 50% of the enriched phosphopeptides were analyzed by nanoflow LC‐MS/MS using an Easy‐nLC 1200 system coupled to an Orbitrap Exploris 480 (both Thermo Fisher Scientific). Samples were separated on a C18 analytical column (Luna C18(2), 3 µm particle, 100 µm ID x 30 cm, Phenomenex, Torrance, CA) using water with 0.1% FA (mobile phase A) and 80% ACN with 0.1% FA (mobile phase B) using a binary gradient ranging from 2%–6% B in 5 min, 6%–30% B in 62 min, 30%–45% B in 7 min, 45%–90% B in 1 min, and with final elution of 90% B for 15 min at a flow rate of 300 nL/min. MS acquisition was conducted in data‐dependent acquisition mode (DDA) with a cycle time of 1.0 s. Full MS scans were acquired at a resolution of 60 000 from 380 to 1500 m/z (AGC 300%, 50 ms max injection time). Precursor ions with charge states between +2 and +6 were isolated with a m/z 1.6 window and fragmented with a normalized collision energy of 30%. MS/MS spectra were acquired at a resolution of 30 000 (AGC 100%, automated max injection time). Dynamic exclusion was set to 15 s.

#### 5.11.6 Data Analysis of Spinal Cord Proteome

Raw data were analyzed using FragPipe 21.1 with MSFragger [162] 4.0, IonQuant 1.10.12, and Philosopher 5.1.0. Data were searched against a mouse SwissProt database (downloaded from uniprot.org on October 23, 2022, with 17,197 target proteins) complemented with decoys and common contaminants. Trypsin was selected as an enzyme with a maximum of 1 missed cleavage, and mass tolerances for recalibration were set to 10 ppm for both MS and MS/MS spectra. Peptide length was restricted to 7‐35 amino acids, carbamidomethylation of Cys (+57.02146 Da) was selected as fixed, and oxidation of Met (+15.9949 Da), pyro‐Glu formation from N‐terminal Gln (‐17.0265 Da) and N‐terminal Glu (‐18.0106 Da) were selected as variable modifications, with a maximum of 3 allowed variable modifications per peptide. MS Booster, Percolator, and Philosopher were used for validation. Label‐free quantitation (LFQ) was performed using IonQuant, enabling matching between runs with an ion false discovery rate of 1% and only using unique peptides for quantitation. The minimum number of scans was set to 5, all other parameters were kept to default settings. Data were filtered to a 1% false discovery rate on peptide and protein levels. A total of 8832 proteins were quantified and further filtered to retain high‐quality data. Thus, all proteins quantified with less than 2 peptides and in less than 3 of 4 replicates of either treated or sham were removed, resulting in a total of 6761 proteins. Differential analysis was performed using moderated t‐test (LIMMA) [88] and considering only 6270 proteins that were quantified in all replicates of either sham or treated samples. Intensities were log2‐transformed, followed by K‐nearest neighbor (KNN) imputation for Missing at Random (MAR) values. A total of 51 proteins with adjusted p‐values <0.05 were considered statistically significantly differential between treated and sham. In addition, 23 proteins that were quantified in 3 replicates of one condition but in no replicate of the other condition were labeled as potentially differential, as well as 2 proteins quantified in 3 replicates of the treated replicates but in only 1 one control replicate with >4 and >10 times lower intensity.

#### 5.11.7 Data Analysis of Spinal Cord Phosphoproteome

Raw data were analyzed using FragPipe 21.1 with MSFragger 4.0, IonQuant 1.10.12, and Philosopher 5.1.0. Data were searched against a mouse swissprot database (downloaded from uniprot.org on January 25, 2024, with 17,197 target proteins) complemented with decoys and common contaminants. Trypsin was selected as an enzyme with a maximum of 2 missed cleavages, and mass tolerances for recalibration were set to 20 ppm for both MS and MS/MS spectra. Peptide length was restricted to 7‐35 amino acids, carbamidomethylation of Cys (+57.02146 Da) was selected as fixed, and oxidation of Met (+15.9949 Da), phosphorylation of Ser/Thr/Tyr (+79.96633 Da), and deamidation of Asn, Gln (+0.984016 Da) were selected as variable modifications, with a maximum of 4 allowed variable modifications per peptide. MS Booster, Percolator, and Philosopher were used for validation, and PTM Prophet was used to determine site localization probabilities of all variable post‐translational modifications. Label‐free quantitation (LFQ) was performed using IonQuant, enabling matching between runs with an ion false discovery rate of 1% and only using unique peptides for quantitation. The minimum number of scans was set to 5, all other parameters were kept to default settings, with a minimum site localization probability of 75%. Data were filtered to a 1% false discovery rate on peptide and protein levels. A total of 9541 phosphorylation sites were quantified and further filtered to retain high‐quality data. Only phosphopeptides that were quantified in all 4 replicates of either treated or sham were kept, resulting in a total of 3945 high‐quality phosphopeptides. Differential analysis was performed using a moderated *t‐test* (LIMMA) [88]. Intensities were log2‐transformed, followed by K‐nearest neighbor (KNN) imputation for Missing at Random (MAR) values. A total of 29 phosphorylation sites with adjusted *p*‐values <0.05 were considered statistically significantly differential between treated and sham.

#### 5.11.8 In Vitro Pharmacokinetic (ADME) Profiling of Borsantrazole (BSZ)

**Bidirectional Caco‐2 permeability**: Caco‐2 cells were seeded onto permeable polycarbonate supports in 12‐well Nest Transwell plates and allowed to grow and differentiate for 21–25 days. On the day of the assay, culture medium (DMEM supplemented with 10% FBS, 1% non‐essential amino acids, and penicillin/streptomycin) was removed from both sides of the transwell inserts, and cells were rinsed with warm HBSS. After the rinse step, the chambers were filled with the transport buffers (For assay reference controls apical: HBSS containing 25 mm MES, 0.25% BSA, pH 6.0; Basolateral: HBSS containing 25 mM HEPES, 0.25% BSA, pH 7.4, and for test compounds apical‐ and basolateral buffer with HBSS containing 25 mm HEPES, 0.25% BSA, pH 7.4) and the plates were incubated at 37°C for 30 min prior to TEER (Trans Epithelial Electric Resistance) measurements. The buffer in the donor chamber (apical side for A‐to‐B assay, basolateral side for B‐to‐A assay) was removed and replaced with the working solution (1 & 10 µm test article in transport buffer). The plates were then placed at 37°C under light agitation. At designated time points (30, 60, and 90 min), an aliquot of transport buffer from the receiver chamber was removed and replenished with fresh transport buffer. Samples were quenched with ice‐cold ACN containing an internal standard and then centrifuged to pellet protein. Resulting supernatants were further diluted with 50/50 acetonitrile/water (except for Atenolol, which was diluted with water). Plates were then submitted for LC‐MS/MS analysis. Reported apparent permeability (Papp) represents the average of 2 determinations. Atenolol and propranolol were tested as low‐ and moderate‐permeability references. Bidirectional transport of digoxin was assessed to demonstrate Pgp activity/expression.

**LogD Assessment**: Stock solutions of the test compounds (10 mM in DMSO) were added to a 50:50 mixture of octanol and 100 mM potassium phosphate buffer (pH 7.4). The mixtures were agitated on an Eppendorf thermomixer at 1000 rpm for 1 h at room temperature. After agitation, the octanol and buffer phases were collected separately and diluted with a 1:1 ethanol–water mixture before LC‐MS/MS analysis. For each experiment, warfarin served as a reference compound for low LogD, while glyburide and albendazole were used as references for medium LogD values. All samples were analyzed in duplicate by LC‐MS/MS using electrospray ionization, with standards prepared in the same matrix.

### 5.12 Statistical Analysis

All statistical analyses were performed using GraphPad Prism software version 9 (GraphPad Software Inc). Data represent the mean and standard error of the mean (SEM). Unpaired two‐tailed Student's *t‐test* was used for the comparison of two means. One‐way AVONA followed by Dunnett's multiple comparisons test was used for the multiple‐group analysis. Kaplan‐Meier method with Log‐rank test was used for disease onset and survival analysis. Multiple corrections tests were used to account for multiple comparisons in statistical hypothesis testing. The significance level for the two‐sided analyses was set at *p < 0.05*. Note: while the Log‐rank (Mantel‐Cox) test was used as the primary measure of probability of onset, symptom onset, and survival significance, an unpaired two‐tailed *t*‐test was also performed on the total days survived to provide a descriptive comparison of mean survival duration.

## Author Contributions

All authors read and approved the final version of the manuscript. **Nitesh Sanghai** synthesized and characterized Borsantrazole (BSZ) and conducted biochemical and cell‐based experiments. He developed the animal protocol together with GKT, designed and performed in vivo animal experiments, conceptualized the study, and wrote the majority of the manuscript. Additionally, he analyzed all data from biochemical assays and in vivo experiments, managed the references, and created all the figures for the manuscript. **Rhonda Kelley** provided support and assistance for animal research studies. **Ying Luo, Immanuel Madlangsakay, Prasanta Paul, and René P. Zahedi** were involved in the formal analysis, investigation, visualization, conceptualization, and writing of the methodology for the proteomics and phosphoproteomics data and sections. **Alejandra Llanes‐Cuesta and Jun‐Feng Wang** supported the experiments to evaluate BSZ's safety profile in PCNC cells, using the infrastructure provided for these experiments. **Jiming Kong** reviewed and edited the manuscript. JK also provided the infrastructure for genotyping and animal surgery and assistance with the animal model, as well as mentoring for the project. **Geoffrey K. Tranmer** conceptualized this project and manuscript, acquired funding, defined the methodology, oversaw the project, and made major contributions to its writing, review and editing.

## Funding

This research was supported by the Research Manitoba 2017 Health Research New Investigator Operating Grant, the Natural Sciences and Engineering Research Council of Canada (NSERC) Discovery Grant, and the Canadian Institutes of Health Research (CIHR) grant to G.K.T.

## Ethics Statement

Animal ethics and all protocols were approved and monitored by the University of Manitoba Central Animal Care Services and Animal Care Committee under protocol 21‐014(AC11693).

## Conflicts of Interest

The authors declare no conflicts of interest.

## Supporting information




**Supporting File**: advs76432‐sup‐0001‐SuppMat.pdf.

## Data Availability

All data generated or analyzed during this project was published as part of this manuscript or supplementary information files, otherwise, all data is available for review on request, including raw proteomic and phosphoproteomic data.
