# Supplementary material for: Introducing Borsantrazole: A Trifunctional Boron‐Based Pyrazole That Extends the Lifespan of Amyotrophic Lateral Sclerosis Mice
Source: Adv Sci (Weinh). 2026 Jul 26;13(54):e76432. doi: 10.1002/advs.76432 (PMC13402437; doi:10.1002/advs.76432)
Supplement: Supplementary file 1 [file ADVS-13-e76432-s001.pdf]

Introducing Borsantrazole: A trifunctional boron-based pyrazole that extends lifespan in ALS mice

Nitesh Sanghai[1], Rhonda Kelley[2], Ying Lao[3], M Immanuel Reyes Madlangsakay[3], Prasanta Paul[3,4], M. Alejandra Llanes-Cuesta[5], Jun-Feng Wang[5], René P. Zahedi[3,4,6], Jiming Kong[7], Geoffrey K. Tranmer[1*]

[1]College of Pharmacy, University of Manitoba, Winnipeg, MB R3E 0T5, Canada. [2]Central Animal Care, University of Manitoba, Winnipeg, MB, R3E 0J8, Canada. [3]Manitoba Centre for Proteomics and Systems Biology, University of Manitoba, Winnipeg, MB, R3E 3P4, Canada. [4]Department of Internal Medicine, University of Manitoba, Winnipeg, MB, R3A 1R9, Canada. [5]Department of Pharmacology and Therapeutics, University of Manitoba, Winnipeg, MB, R3E 0T6, Canada. [6]Department of Biochemistry and Medical Genetics, University of Manitoba, Winnipeg, MB, R3E 0J9, Canada. [7]Department of Human Anatomy and Cell Science, University of Manitoba, Winnipeg, MB, R3E 0J9, Canada.

***Correspondence to:**

Geoffrey K. Tranmer
College of Pharmacy, Rady Faculty of Health Science, University of Manitoba, Winnipeg, MB R3E 0T5, Canada.
Email: geoffrey.tranmer@umanitoba.ca
Phone: +12044748358

Supplementary Files –Synthesis Experimental Methods

Experimental

General considerations: ^{1}H and ^{13}C nuclear magnetic resonance (NMR) spectra were recorded on a Bruker 500 MHz spectrometer, Billerica, MA, USA, using DMSO-d_6 **(CAS-2206-27-1)**, as a solvent with tetramethylsilane (TMS) as an internal standard. *J* values are given in Hz. Mass spectra were recorded Bruker Daltonik GmbH, LCMS–100-600 [esi.m], Orbitrap Exploris 480, 60K Resolution, ESI Thermofisher [for HRMS] mass spectrometers. Microwave reactions carried out in the CEM explorer hybrid-12 microwave reactor. n-Butyllithium,2.5M solution in hexanes, Acros organics **(CAS-109-72-8)** product of Germany; 2-Isopropoxy-4,4,5,5-tetramethyl-1,3,2-dioxaborolane **(CAS-61676-62-8)** purchased from Sigma-Aldrich, St. Louis, MO, USA; 3-Methyl-1-phenyl-1H-pyrazole (**Number: AK139802**) was purchased from Ark Pharm, Inc., Arlington Heights, IL, USA. Hydrogen peroxide (H_2O_2) **(CAS-7722-84-1)** purchased from Sigma-Aldrich, St. Louis, MO, USA. Lactic acid (CAS-7732-18-5) was purchased from Acros Organics. The reactions were monitored by TLC (Sigma, Silica gel 60 F_{254}). The crude reaction mixture was purified with silica gel column chromatography on a CombiFlash® Rf 200 purification system, Teledyne Isco, USA. Organic solvents were ordered from BDH, VWR Analytical, unless specified otherwise. All chemicals were used without further purification unless stated otherwise.

Synthesis of 3-methyl-5-(4,4,5,5-tetramethyl-1,3,2-dioxaborolan-2-yl)-1-phenyl1H-pyrazole (Borsantrazole (BSZ), Fig. 15, Main Article)

Representative experimental procedure for the synthesis of 3-methyl-5-(4,4,5,5-tetramethyl-1,3,2-dioxaborolan-2-yl)-1-phenyl1H-pyrazole (NS-1-2).

Synthesis of Edaravone prodrug was carried out according to the reported procedure [170] with slight modifications, scheme I, **Figure 15**. n-Butyllithium (2.5 M in hexane, 1.5 cm3, 3.793 mmol) was added dropwise to a solution of N-arylated substituted pyrazole (500mg, 0.471cm3, 3.161mmol) in anhydrous THF (22 cm3) at -78 °C under argon. The reaction mixture was stirred for 45 minutes at -78°C. 2-Isopropoxy-4,4,5,5-tetramethyl1,3,2-dioxaborolane (646.93mg, 709.5μl, 3.477 mmol) was added dropwise to the reaction mixture at -78°C, and the mixture was stirred for 1.5 h. The mixture warmed to room temperature over 1h and glacial acetic acid (208.79mg, 199 μl, 3.477 mmol) was added. The mixture was filtered through

a celite pad, which was washed with EtOAc (100 cm3). The organic solvent was removed in vacuo to afford a crude product. TLC confirmed the expected product (20% EtOAc/Hex). The crude product was then purified with silica gel column chromatography on a CombiFlash® Rf 200 purification system, Teledyne Isco, USA, with ethyl acetate and hexane from 0% to 10%. Residual solvent was evaporated under vacuum to a final light brown crystalline solid product (815mg, 90%).

Microwave synthesis of 3-methyl-1-phenyl-2-pyrazolin-5-one (Edaravone) from its prodrug 3-methyl-5-(4,4,5,5-tetramethyl-1,3,2-dioxaborolan-2-yl)-1-phenyl1H-pyrazole (BSZ).

The synthesis of Edaravone from its prodrug NS-1-2 is influenced by the bio-inspired catalyst-free reported procedure[171]. Reactions carried out in microwave irradiations (300Psi, premixed 15sec, 200watt) were performed on a 0.5mmol scale. N-phenyl-3-methylpyrazole boronic ester (1.0 equiv.) and diethyl ether (3.5mL in excess) were added to a 10mL microwave vial equipped with a stir bar, followed by the addition of lactic acid (10 equiv.) and hydrogen peroxide (30%w/w,1.1 equiv.). The mixture was then capped and placed in the microwave reactor and heated to 50 °C for 10 min. After completion of the reaction, the reaction was allowed to cool at RT. The vial was removed from the microwave, and the small aliquot was taken for TLC analysis. A single new spot corresponding to Edaravone was observed under U.V. in TLC. The reaction mixture was diluted with water (5 mL) and extracted with (10 mL ethyl acetate and 2 mL hexane). The organic extract was dried in vacuo and subjected to flash chromatography (EtOAc/hexanes) to afford the desired product. The purified solution obtained after flash chromatography was dried in vacuo to obtain edaravone in the form of clear oil.

Recrystallization: The clear oily oil was cooled in the ice bath, and a small amount of diethyl ether dropwise was added (0.5-1mL). The resulting oily solution was stirred vigorously in a water bath sonicator for 5 minutes. The white crystalline solid reappeared during sonication. The resulting white crystalline solid was dried completely under vacuum to obtain pure Edaravone. The purified product was sealed with paraffin in the glass vial and stored under 40°C. Compound NS-1-10 (120mg, 84.50%, product chromatogram NS-1-10 purified) was prepared by this method and imparted 1-d-proton NMR data similar to those reported in the literature for Edaravone.

TLC Observations:

TLC observation of Crude reaction mixture. The TLC was carried out in the solvent system of 50% ethyl acetate and hexane. Rf was observed for Edaravone, the crude reaction mixture, and the isolated product, which was found to be equal, i.e., 0.55. Following this reaction procedure, the edaravone prodrug BSZ and Edaravone were synthesized. Their structures were characterized by 1H and 13C NMR and Mass confirmed by HRMS (ESI).

3-methyl-5-(4,4,5,5-tetramethyl-1,3,2-dioxaborolan-2-yl)-1-phenyl1H-pyrazole (Borsantrazole): Prodrug of Edaravone. Crystalline solid (815mg, 90%); 1H NMR (500 MHz, DMSO-d6). δH(500 MHz; DMSO-d6; Me4Si) 3.63 (6 H, s), δ 7.50-7.48 (2H, m, *J* = 10Hz), δ 7.47-7.44 (2H, m, *J* = 15 Hz),7.39-7.36 (1H, m, *J* = 15Hz), δ 6.64(1H, S), δ 2.32(3H, S) δ 1.25(12H, S); 13C NMR (500MHz, DMSO-d6) δ 149.2, 141.2, 128.9, 127.5, 117.8, 84.5, 82.7, 24.7, 13.2 (C ipso to B not observed). 11B NMR (500MHz, DMSO-d6) δ 27.8. 1.8. HRMS (ESI) Calcd for C16H21B1N2O2 [M + H]$^+$ 285.17686., found *m/z* 285.176.

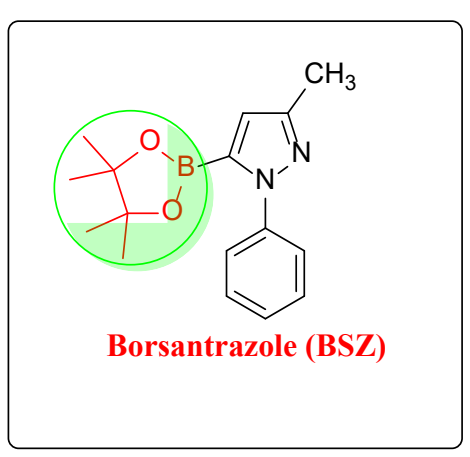

3-methyl-1-phenyl-2-pyrazolin-5-one (NS-1-10): Edaravone.

Crystalline solid (120mg, 84.50%); 1H NMR (500 MHz, DMSO-d6). δH(500 MHz; DMSO-d6; Me4Si) 11.42 (OH, s), δ 7.70-7.69 (2H, d, J = 5Hz), δ 7.42-7.39 (2H, t, J = 15 Hz),7.21-7.18 (1H, t, J = 15Hz), δ 5.37(1H, S), δ 2.11-2.10(3H, d); 13C NMR (500MHz, DMSO-d6) δ 171.7, 158.9, 153.5, 139.4, 138.6, 129.2, 125.4, 124.8, 121.0, 118.4, 88.0, 43.4, 17.1, 14.7.HRMS (ESI) Calcd for C10H10N2O [M + H]+ 175.087753., found *m/z* 175.086.

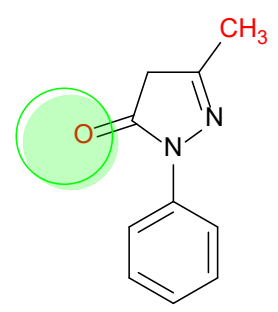

Edaravone (EDR)

Sample: NS-1-2 (BSZ) **Rf+** **Friday 30 May 2025 04:43PM**

RediSep Column: Silica 12g
SN: E04150D568749C Lot: 301621207X
Flow Rate: 30 ml/min
Equilibration Volume: 100.8 ml
Initial Waste: 0.0 ml
Air Purge: 0.5 min
Solvent A: Hexane
Solvent B: Ethyl Acetate

Peak Tube Volume: Max.
Non-Peak Tube Volume: Max.
Loading Type: Solid (Pause)
Wavelength 1 (red): 254nm
Peak Width: 1 min
Threshold: 0.20 AU
Wavelength 2 (purple): 280nm

Run Notes:

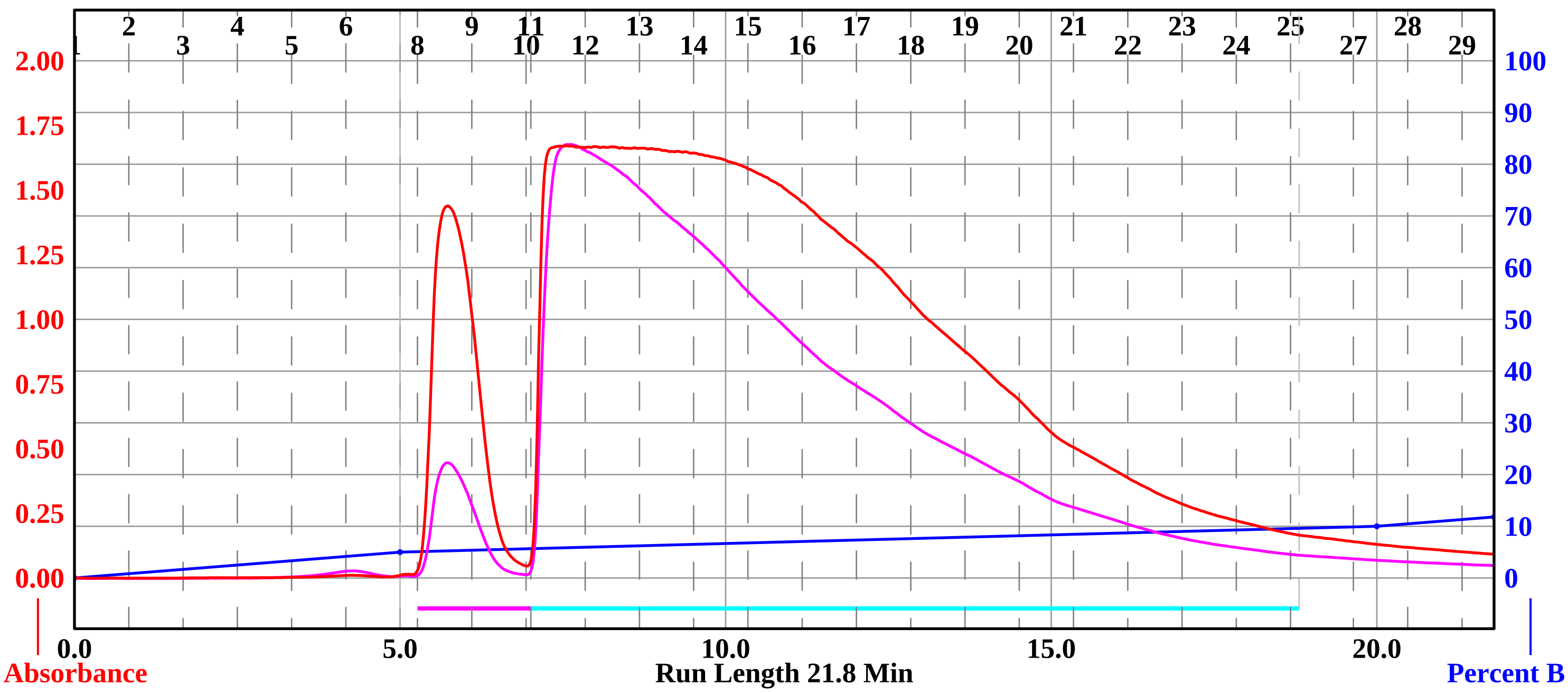

Rack A

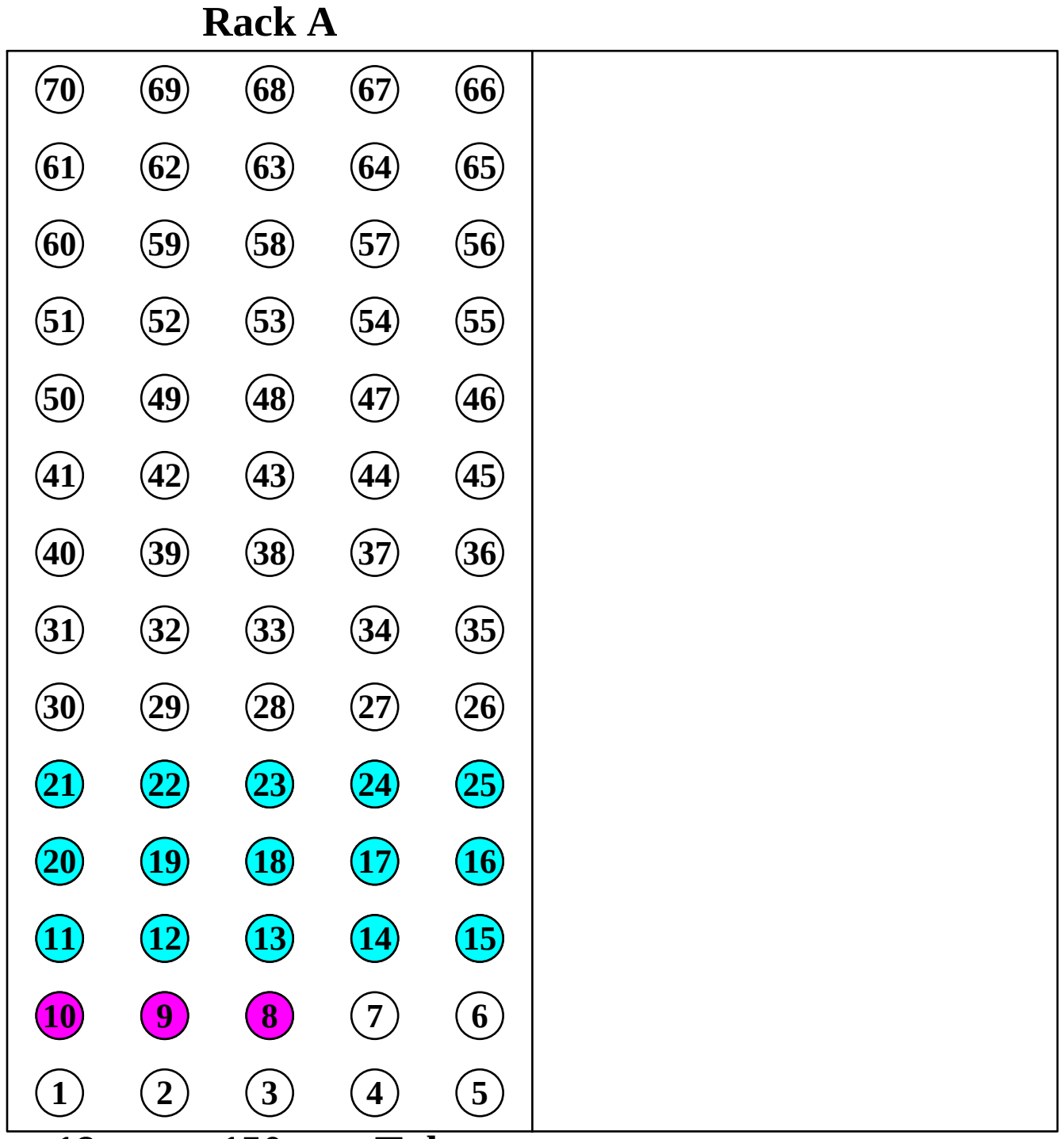

18 mm x 150 mm Tubes

Peak #	Start Tube	End Tube
1	A:8	A:10
2	A:11	A:25

Duration	%B	Solvent A	Solvent B
0.0	0.0	Hexane	Ethyl Acetate
5.0	5.0	Hexane	Ethyl Acetate
15.0	10.0	Hexane	Ethyl Acetate
1.8	11.8	Hexane	Ethyl Acetate

NS-1-2
1H 1D 16 Scans
UofM AVIII 500
27 May 2024

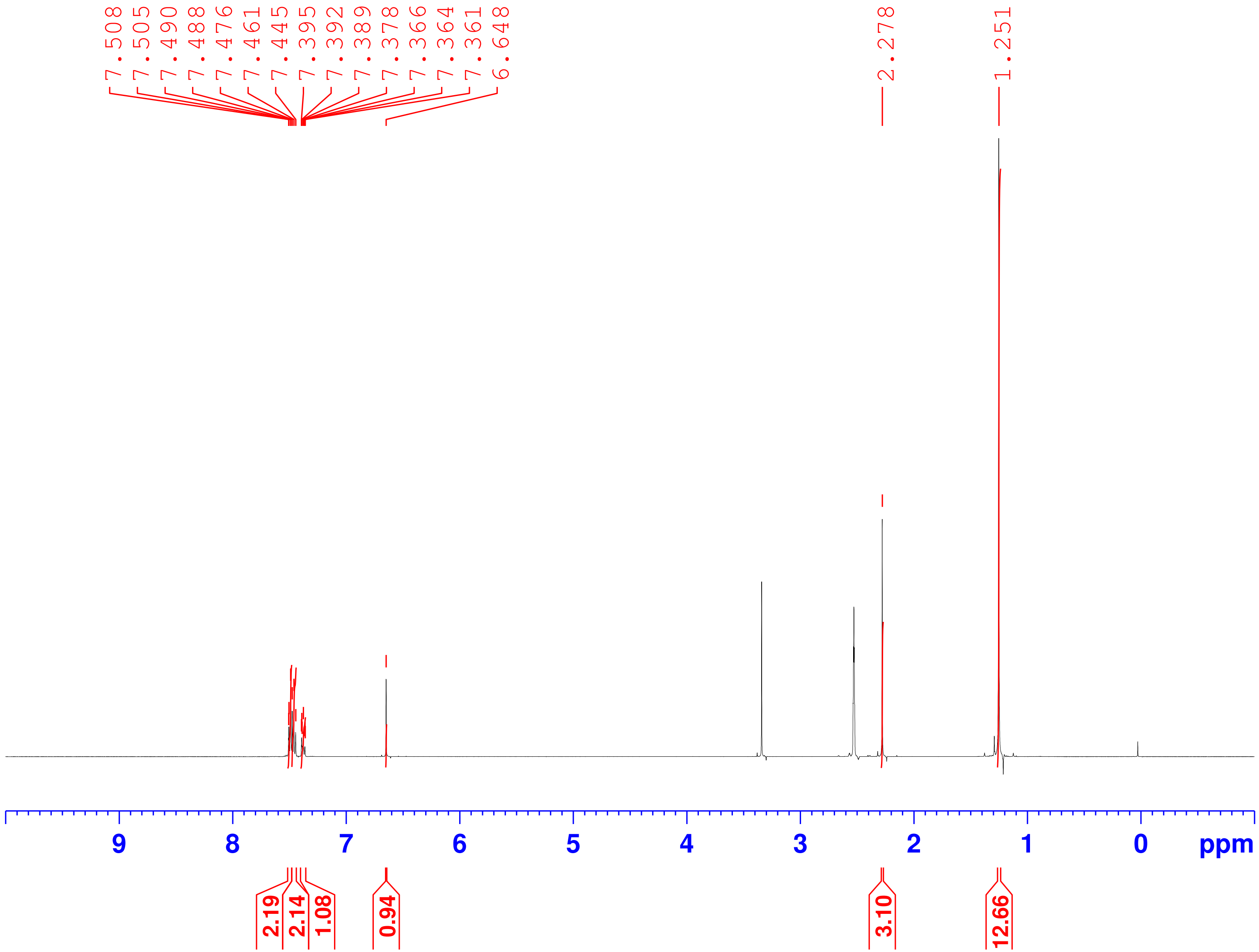

Current Data Parameters

NAME	NS-1-2
EXPNO	1
PROCNO	1

F2 - Acquisition Parameters

Date_	20240527
Time	10.57 h
INSTRUM	spect
PROBHD	Z113652_0056 (
PULPROG	zg30
TD	65536
SOLVENT	DMSO
NS	16
DS	2
SWH	10000.000 Hz
FIDRES	0.305176 Hz
AQ	3.2767999 sec
RG	203
DW	50.000 usec
DE	13.08 usec
TE	298.1 K
D1	1.00000000 sec
TD0	1
SFO1	500.1330885 MHz
NUC1	1H
P1	12.00 usec
PLW1	15.99600029 W

F2 - Processing parameters

SI	65536
SF	500.1299901 MHz
WDW	EM
SSB	0
LB	0.30 Hz
GB	0
PC	1.00

NS-1-2
13C 1D 1024 Scans
UofM AVIII 500
27 May 2024

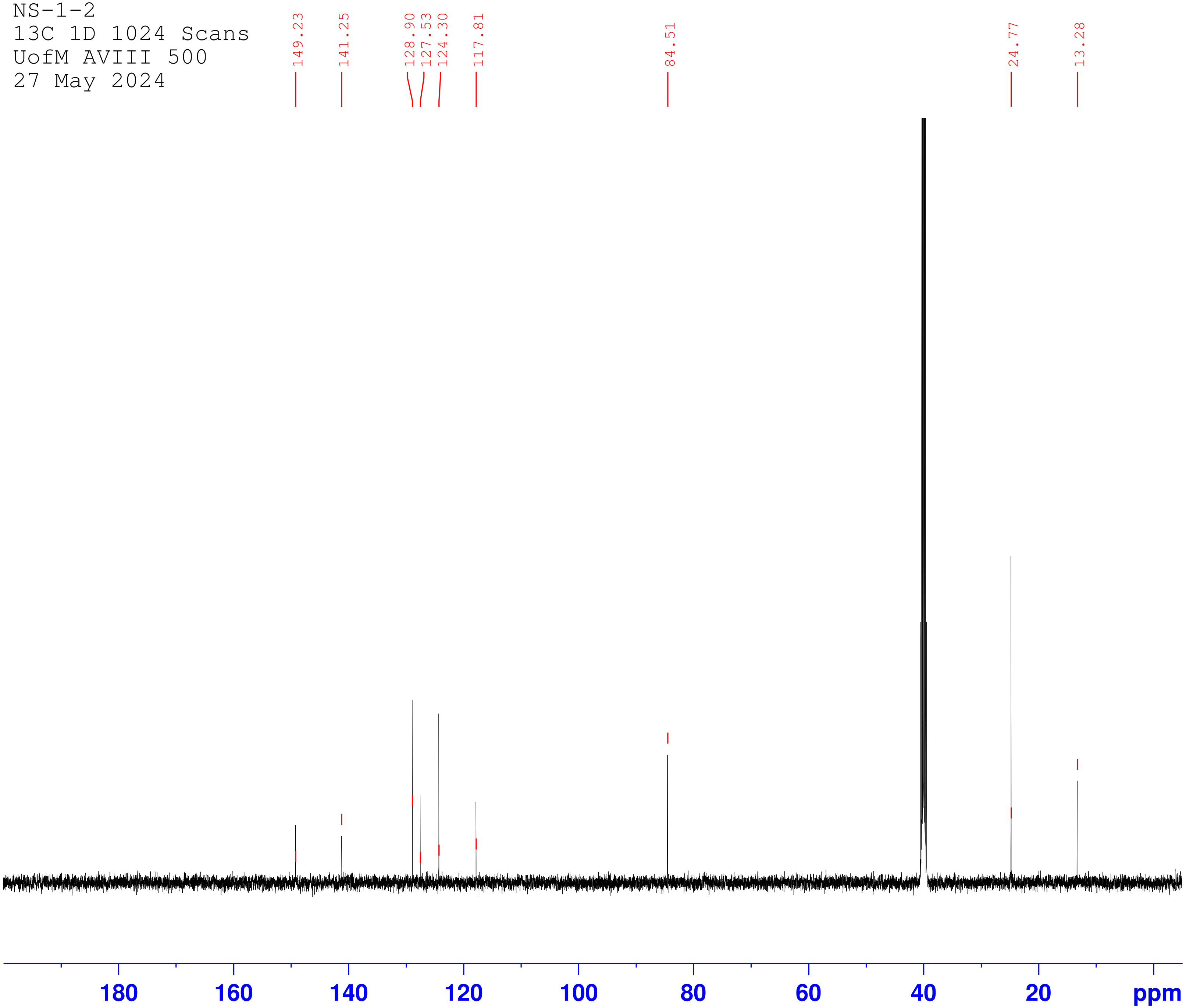

Current Data Parameters
NAME NS-1-2
EXPNO 3
PROCNO 1

F2 - Acquisition Parameters
Date_ 20240527
Time 12.15 h
INSTRUM spect
PROBHD Z113652_0056 (
PULPROG zgpg30
TD 65536
SOLVENT DMSO
NS 1024
DS 4
SWH 29761.904 Hz
FIDRES 0.908261 Hz
AQ 1.1010048 sec
RG 2050
DW 16.800 usec
DE 9.66 usec
TE 298.0 K
D1 2.00000000 sec
D11 0.03000000 sec
TD0 1
SFO1 125.7703637 MHz
NUC1 13C
P1 8.90 usec
PLW1 100.00000000 W
SFO2 500.1320005 MHz
NUC2 1H
CPDPRG[2 waltz16
PCPD2 80.00 usec
PLW2 15.99600029 W
PLW12 0.35991001 W
PLW13 0.18103001 W

F2 - Processing parameters
SI 32768
SF 125.7577936 MHz
WDW EM
SSB 0
LB 1.00 Hz
GB 0
PC 1.40

NS-1-2
11B 1D 128 Scans
UofM AVIII 500
27 May 2024

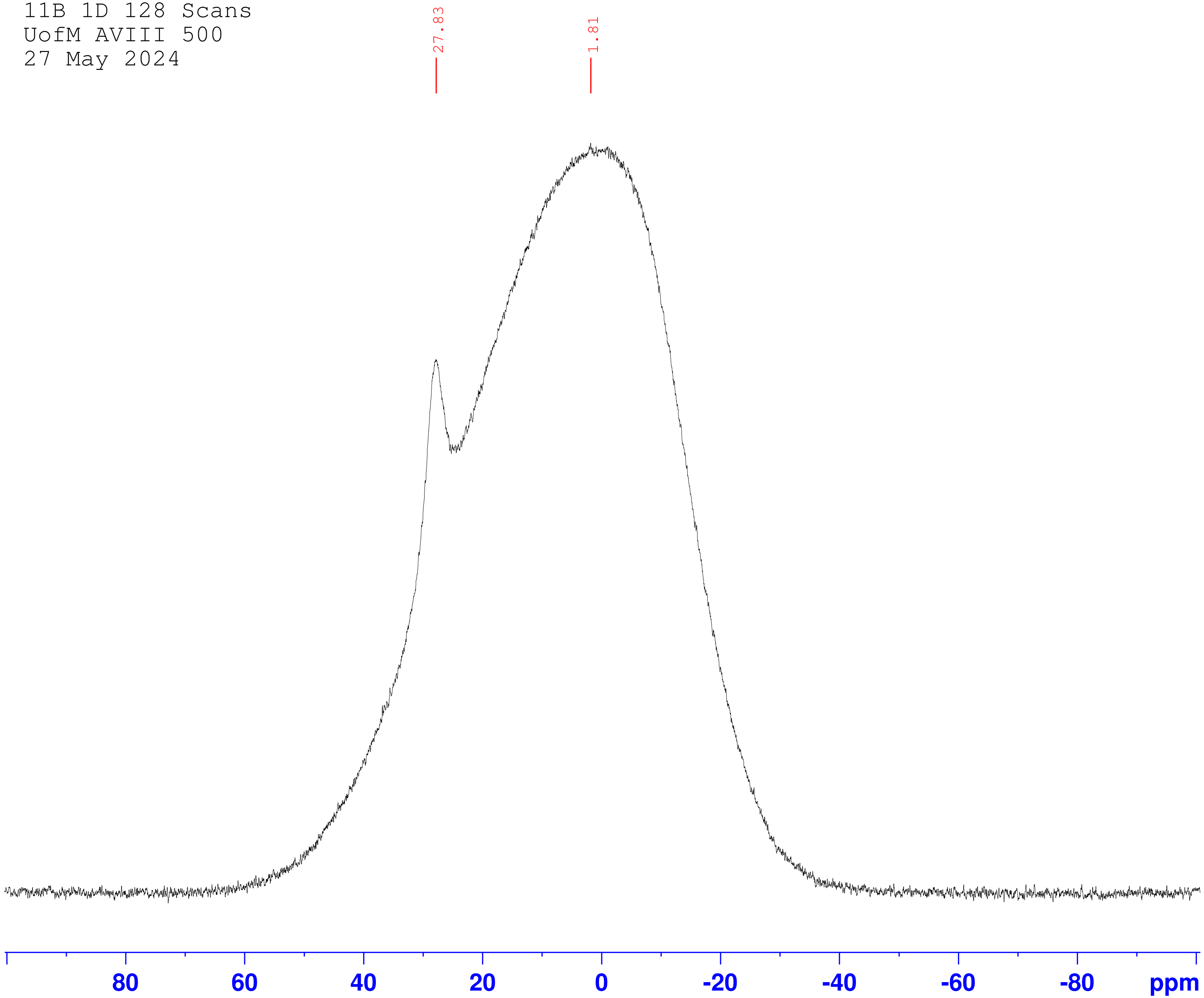

Current Data Parameters

NAME	NS-1-2
EXPNO	4
PROCNO	1

F2 - Acquisition Parameters

Date_	20240527
Time	11.19 h
INSTRUM	spect
PROBHD	Z113652_0056 (
PULPROG	zgpg
TD	65536
SOLVENT	DMSO
NS	128
DS	4
SWH	32258.064 Hz
FIDRES	0.984438 Hz
AQ	1.0158080 sec
RG	1030
DW	15.500 usec
DE	8.32 usec
TE	298.1 K
D1	2.00000000 sec
D11	0.03000000 sec
TD0	1
SFO1	160.4615790 MHz
NUC1	11B
P1	10.00 usec
PLW1	100.00000000 W
SFO2	500.1320005 MHz
NUC2	1H
CPDPRG[2	waltz16
PCPD2	80.00 usec
PLW2	15.99600029 W
PLW12	0.35991001 W
PLW13	0.18103001 W

F2 - Processing parameters

SI	32768
SF	160.4616072 MHz
WDW	EM
SSB	0
LB	10.00 Hz
GB	0
PC	1.40

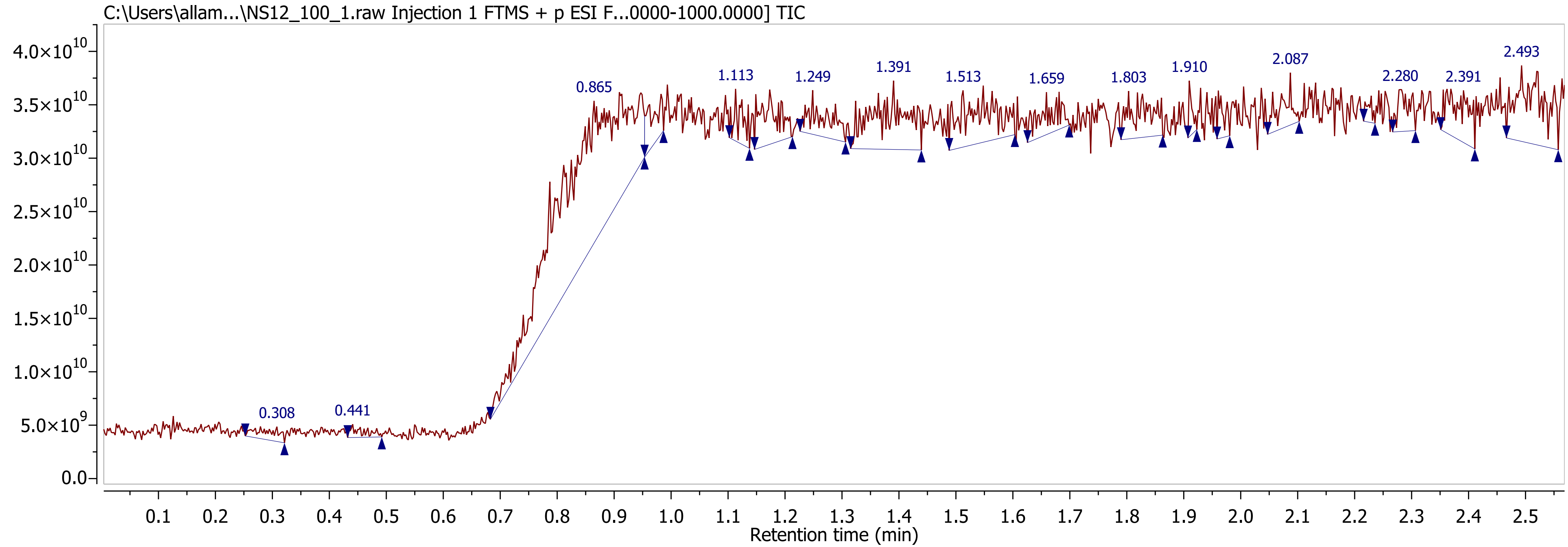
C:\Users\allam...\NS12_100_1.raw Injection 1 FTMS + p ESI F...0000-1000.0000] TIC
0.308
0.441
0.865
1.113
1.249
1.391
1.513
1.659
1.803
1.910
2.087
2.280
2.391
2.493
Retention time (min)

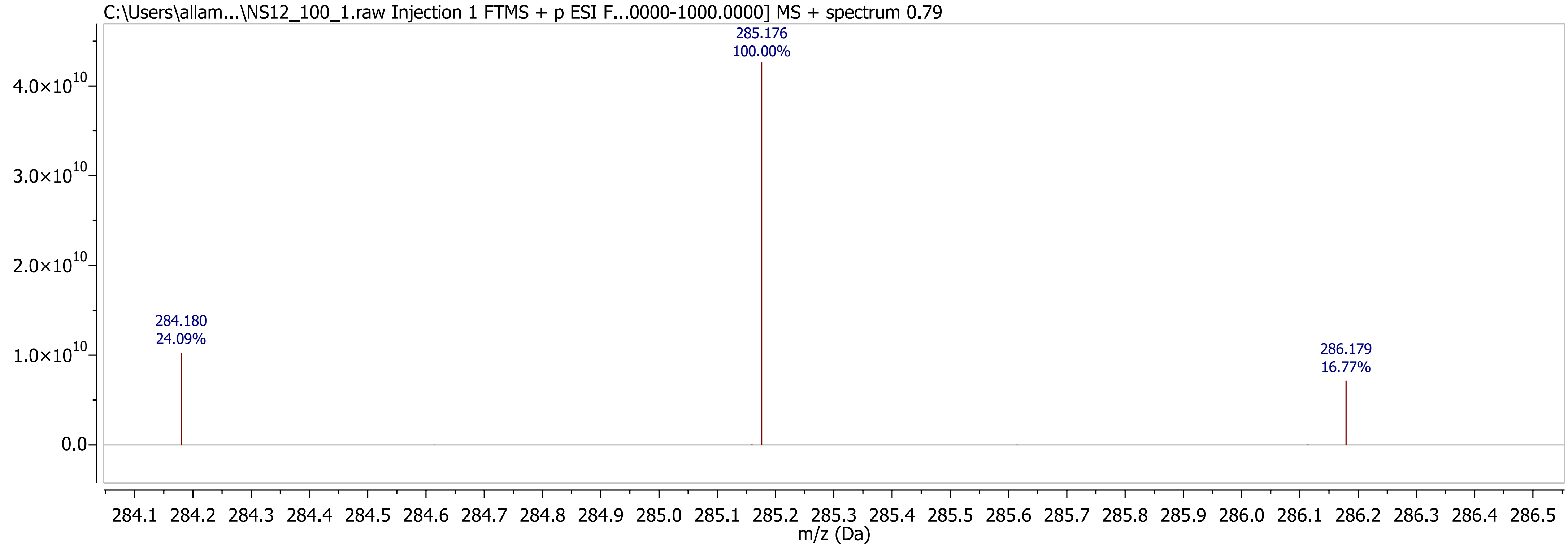
C:\Users\allam...\NS12_100_1.raw Injection 1 FTMS + p ESI F...0000-1000.0000] MS + spectrum 0.79
284.180
24.09%
285.176
100.00%
286.179
16.77%
m/z (Da)

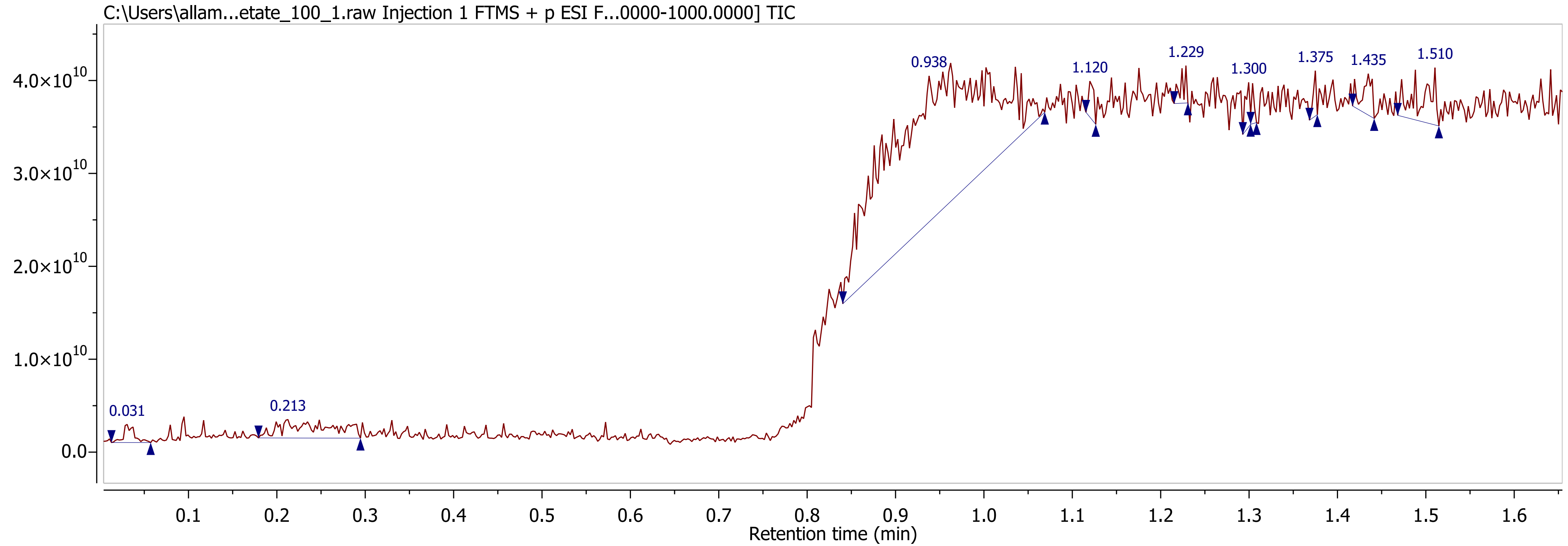
C:\Users\allam...etate_100_1.raw Injection 1 FTMS + p ESI F...0000-1000.0000] TIC
0.031
0.213
0.938
1.120
1.229
1.300
1.375
1.435
1.510
Retention time (min)

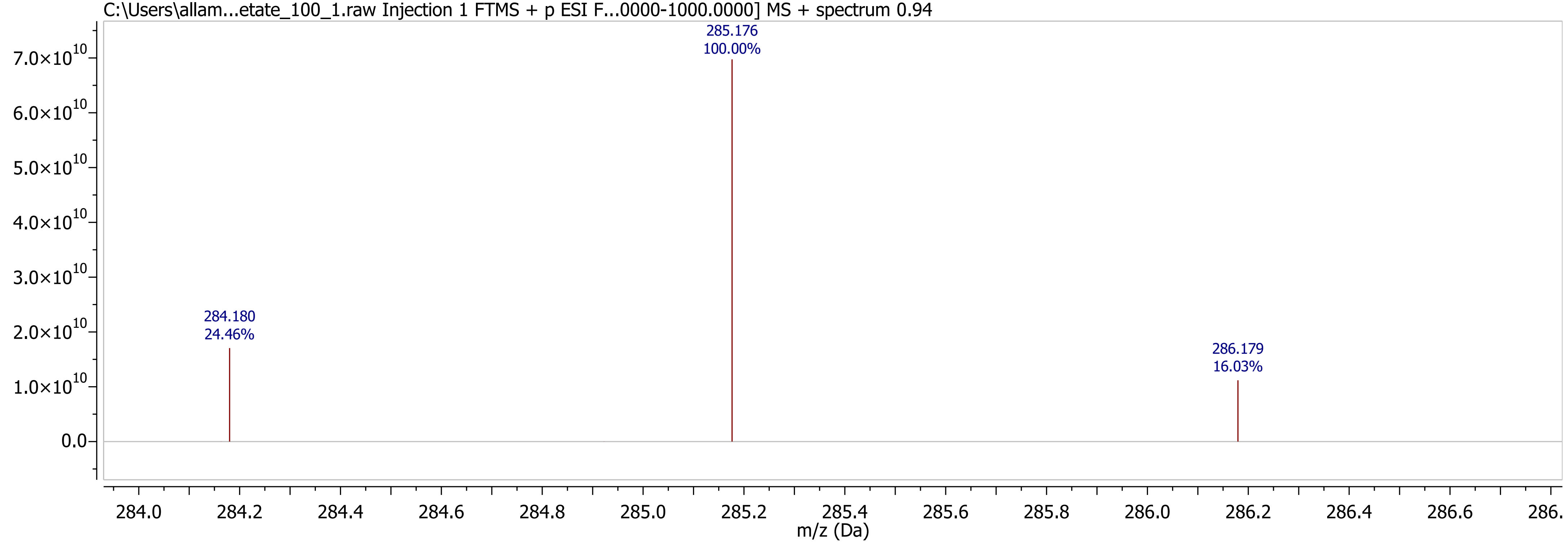
C:\Users\allam...etate_100_1.raw Injection 1 FTMS + p ESI F...0000-1000.0000] MS + spectrum 0.94
285.176
100.00%
284.180
24.46%
286.179
16.03%
m/z (Da)

Sample: EDR **Rf 200** **Friday 05 April 2019 04:00PM**

RediSep Column: Silica 12g
SN: E041503AE4B628 Lot: 2616305030W
Flow Rate: 30 ml/min
Equilibration Volume: 100.8 ml
Initial Waste: 0.0 ml
Air Purge: 0.5 min
Solvent A: hexane
Solvent B: ethyl acetate

Peak Tube Volume: Max.
Non-Peak Tube Volume: Max.
Loading Type: Solid
Wavelength 1 (red): 254nm
Peak Width: 1 min
Threshold: 0.20 AU
Wavelength 2 (purple): 280nm

Run Notes:

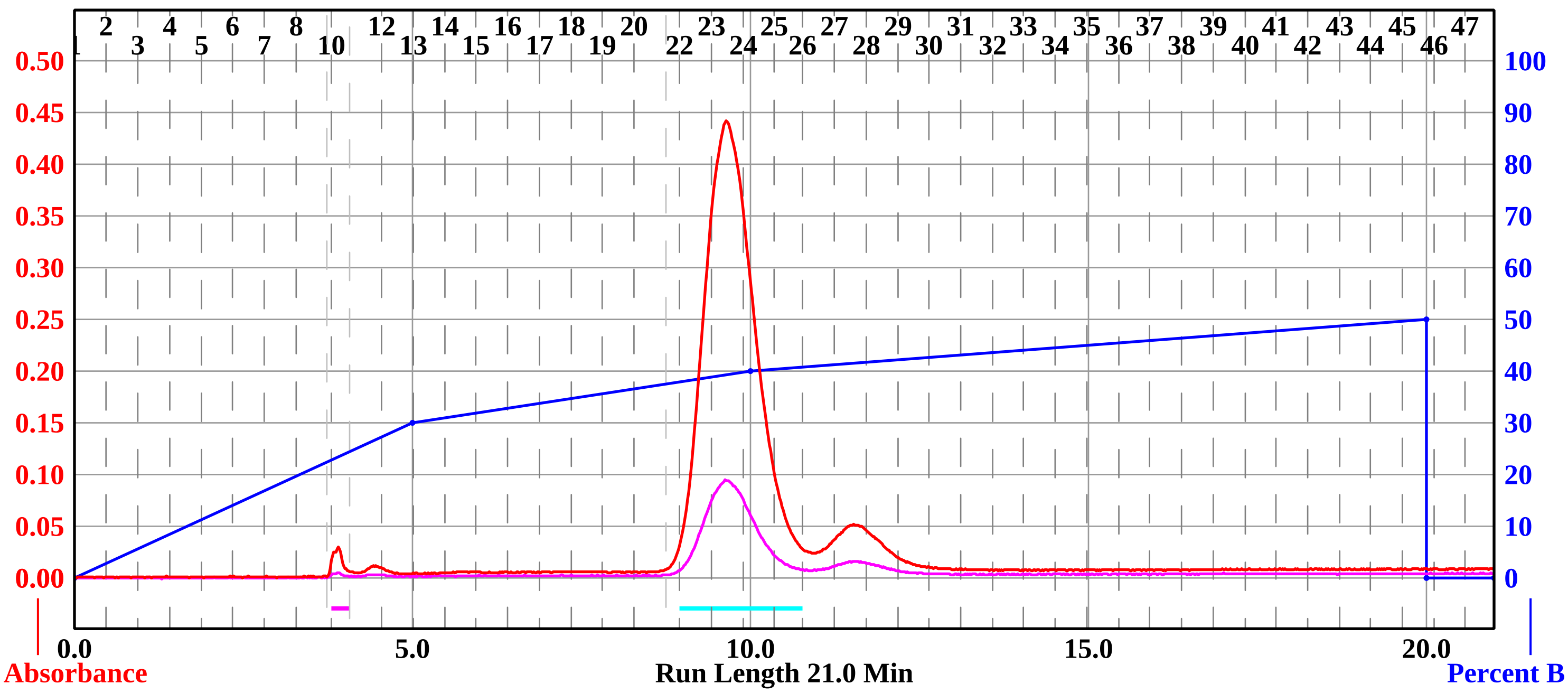

Rack A

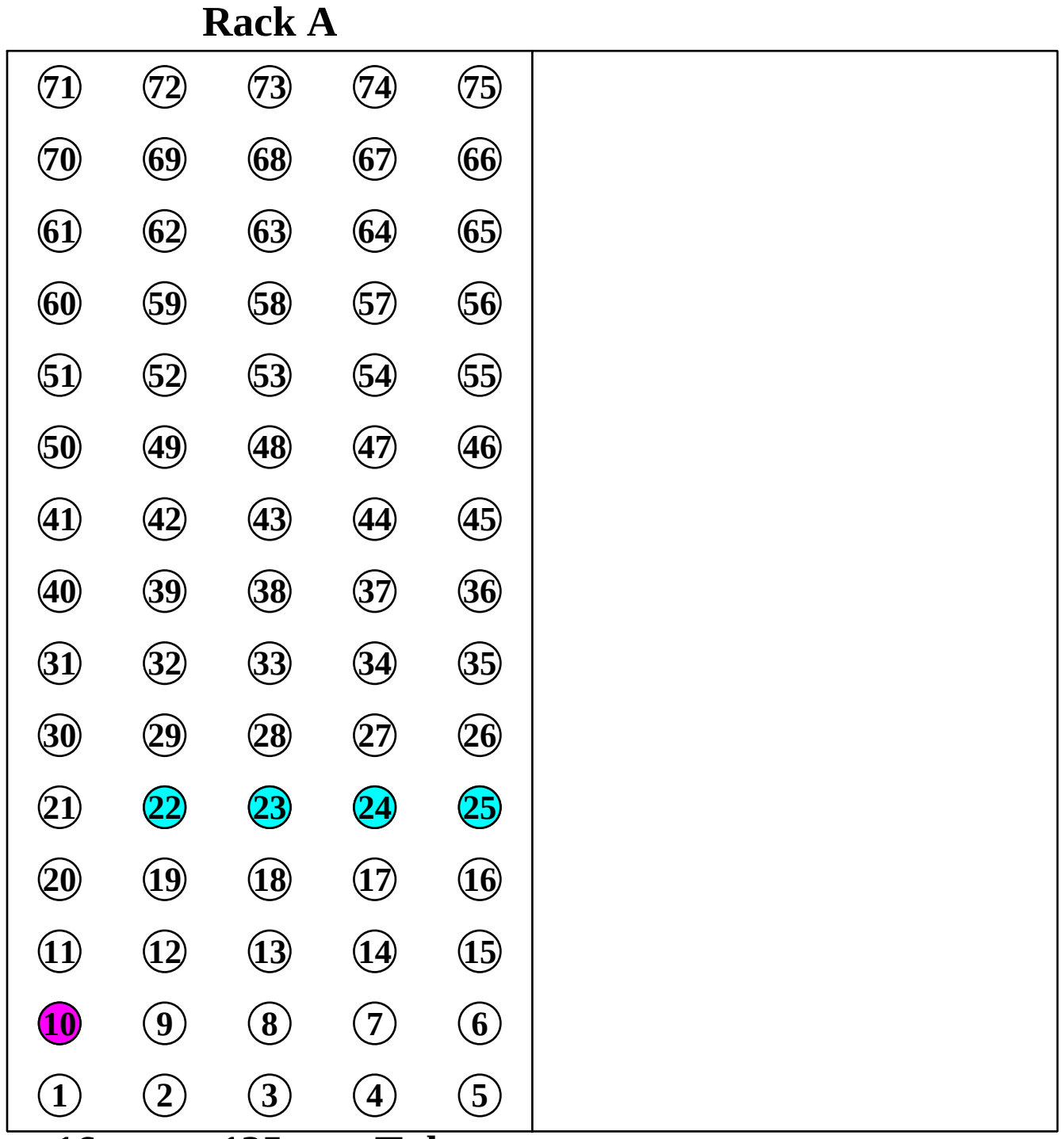

16 mm x 125 mm Tubes

Peak #	Start Tube	End Tube
1	A:10	A:10
2	A:22	A:25

Duration	%B	Solvent A	Solvent B
0.0	0.0	hexane	ethyl acetate
5.0	30.0	hexane	ethyl acetate
5.0	40.0	hexane	ethyl acetate
10.0	50.0	hexane	ethyl acetate
0.0	0.0	hexane	ethyl acetate
1.0	0.0	hexane	ethyl acetate

Nitesh-EDRAVONE
1H 1D 64 Scans
DMSO
UofM AVIII 500
21 October 2024

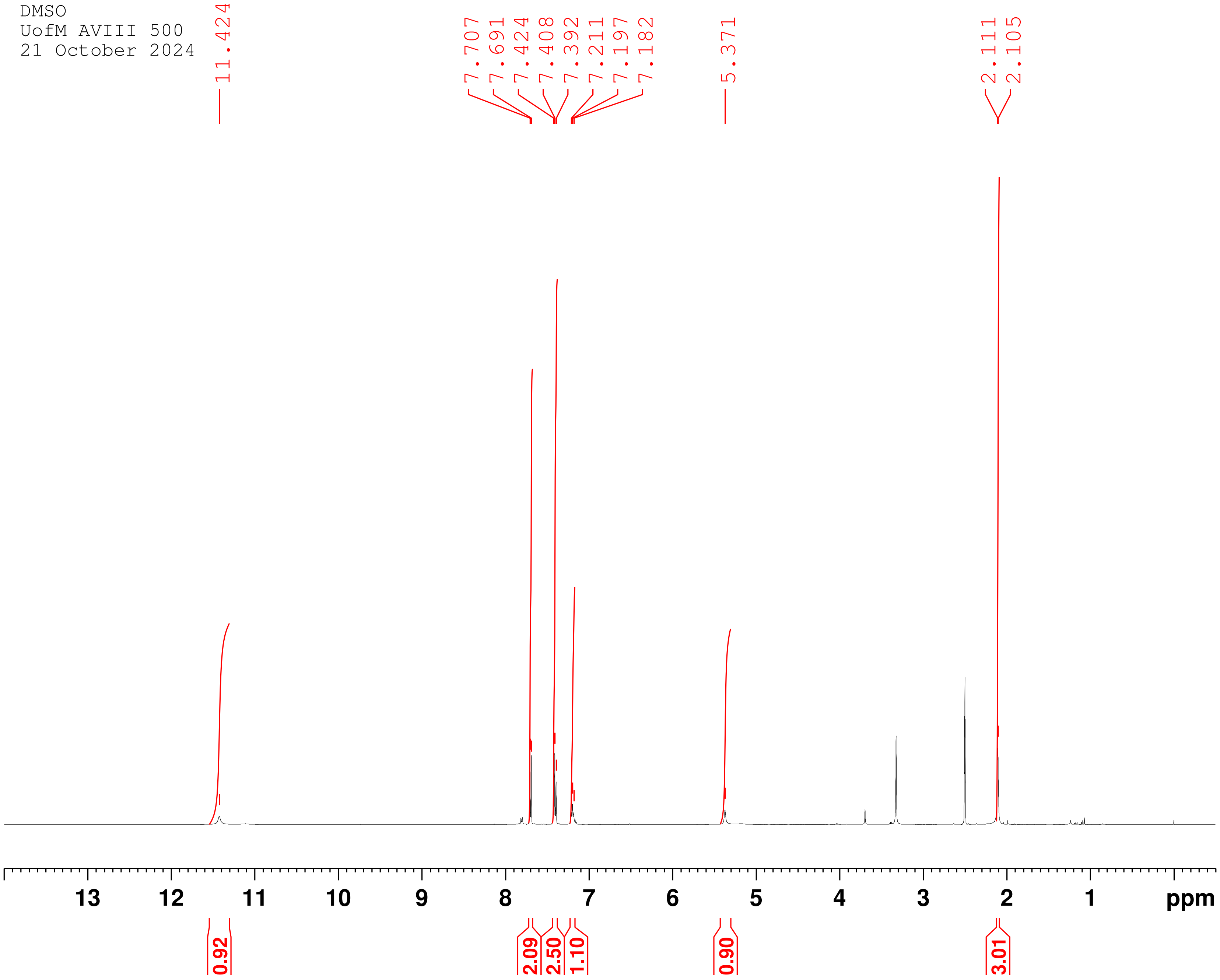

Current Data Parameters

NAME	Nitesh-EDRAVONE
EXPNO	1
PROCNO	1

F2 - Acquisition Parameters

Date_	20241021
Time	11.41 h
INSTRUM	spect
PROBHD	Z113652_0056 (
PULPROG	zg30
TD	65536
SOLVENT	DMSO
NS	64
DS	2
SWH	10000.000 Hz
FIDRES	0.305176 Hz
AQ	3.2767999 sec
RG	203
DW	50.000 usec
DE	13.55 usec
TE	298.0 K
D1	1.00000000 sec
TD0	1
SFO1	500.1330885 MHz
NUC1	1H
P0	4.00 usec
P1	12.00 usec
PLW1	15.99600029 W

F2 - Processing parameters

SI	65536
SF	500.1300041 MHz
WDW	EM
SSB	0
LB	0.30 Hz
GB	0
PC	1.00

EDARAVONE
13C 1D 4096 Scans
23 October 2024

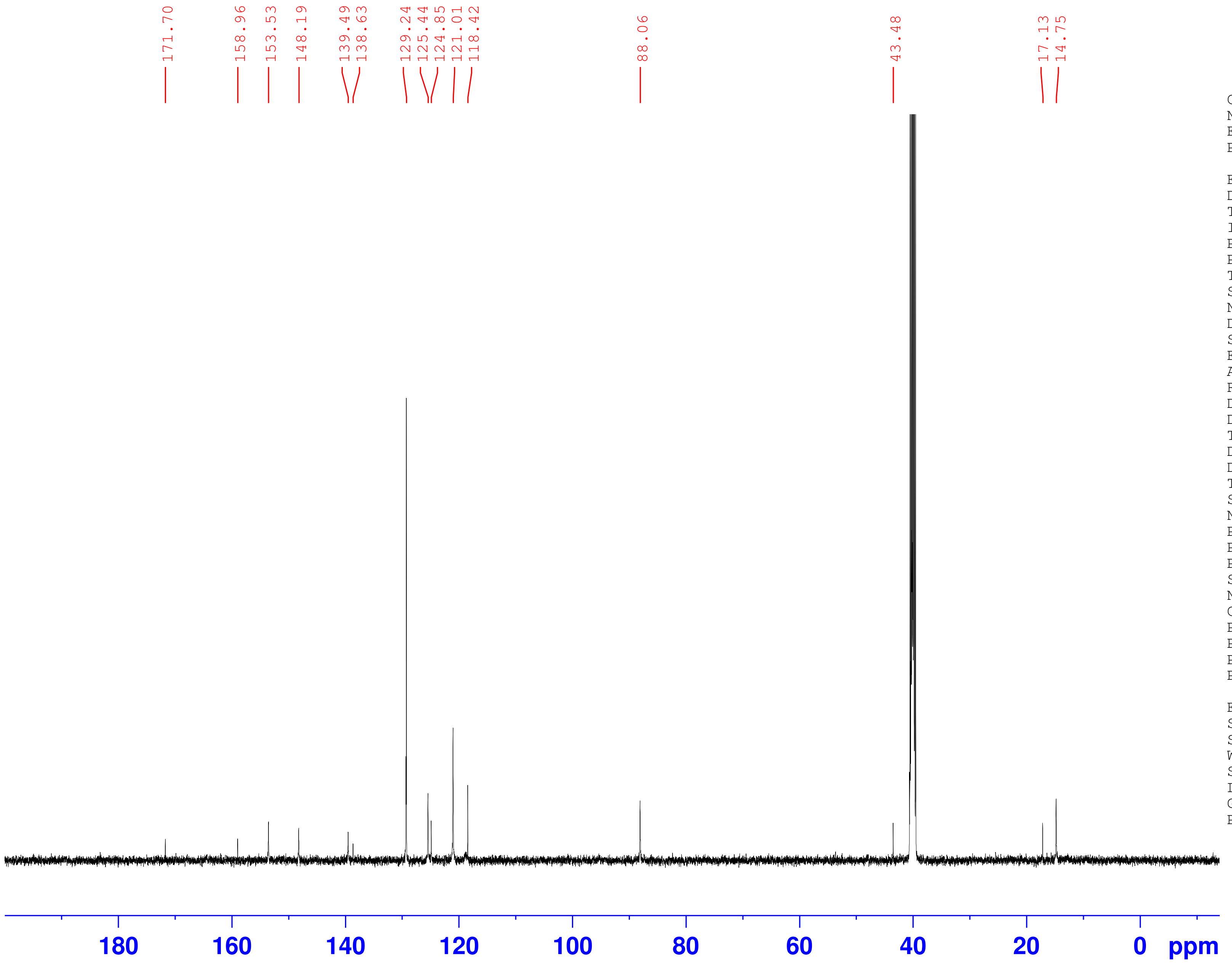

Current Data Parameters
NAME Edaravone C13_New Data
EXPNO 13
PROCNO 1

F2 - Acquisition Parameters
Date_ 20241023
Time 19.10 h
INSTRUM spect
PROBHD Z113652_0056 (
PULPROG zgpg30
TD 65536
SOLVENT DMSO
NS 4096
DS 4
SWH 29761.904 Hz
FIDRES 0.908261 Hz
AQ 1.1010048 sec
RG 2050
DW 16.800 usec
DE 10.01 usec
TE 298.0 K
D1 2.00000000 sec
D11 0.03000000 sec
TD0 1
SFO1 125.7703637 MHz
NUC1 13C
P0 2.97 usec
P1 8.90 usec
PLW1 100.00000000 W
SFO2 500.1320005 MHz
NUC2 1H
CPDPRG[2 waltz16
PCPD2 80.00 usec
PLW2 15.99600029 W
PLW12 0.35991001 W
PLW13 0.18103001 W

F2 - Processing parameters
SI 32768
SF 125.7577890 MHz
WDW EM
SSB 0
LB 1.00 Hz
GB 0
PC 1.40

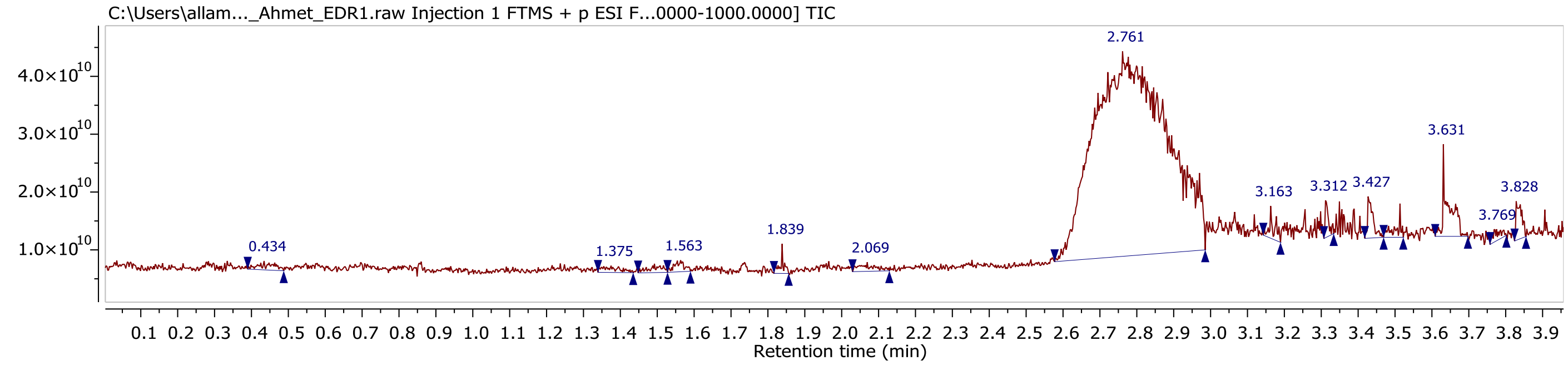
C:\Users\allam..._Ahmet_EDR1.raw Injection 1 FTMS + p ESI F...0000-1000.0000] TIC
2.761
3.631
3.163
3.312
3.427
3.828
3.769
0.434
1.375
1.563
1.839
2.069
Retention time (min)

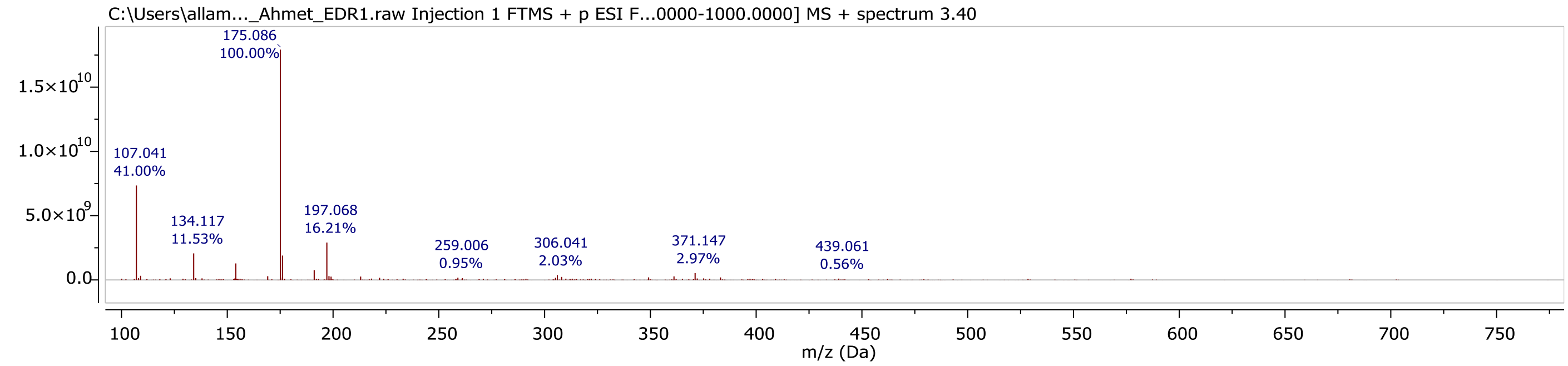
C:\Users\allam..._Ahmet_EDR1.raw Injection 1 FTMS + p ESI F...0000-1000.0000] MS + spectrum 3.40
175.086
100.00%
107.041
41.00%
134.117
11.53%
197.068
16.21%
259.006
0.95%
306.041
2.03%
371.147
2.97%
439.061
0.56%
m/z (Da)

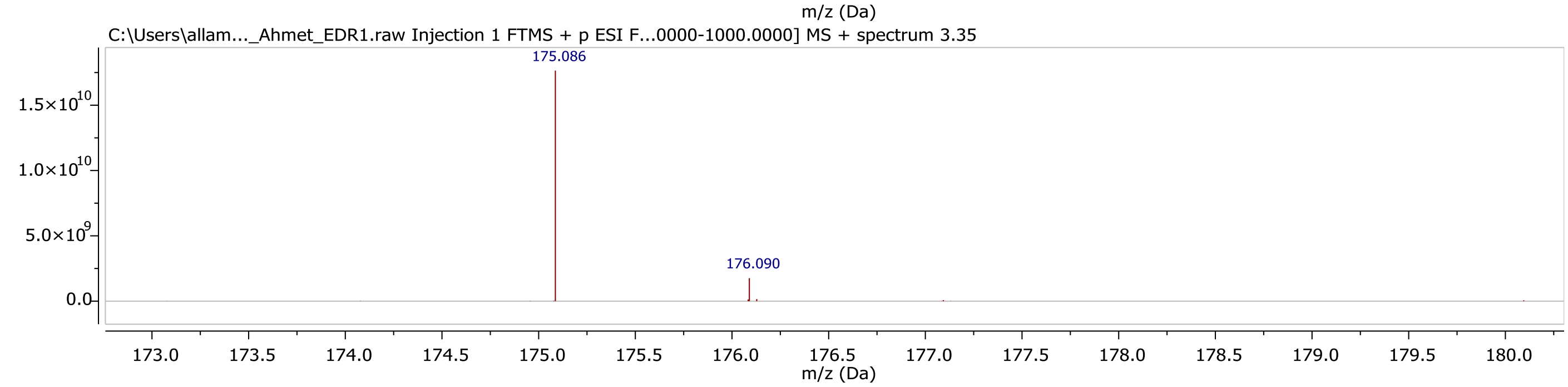
C:\Users\allam..._Ahmet_EDR1.raw Injection 1 FTMS + p ESI F...0000-1000.0000] MS + spectrum 3.35
175.086
176.090
m/z (Da)

NS-1-2 was originally developed in the lab. Later on, trademarked as Borsantrazole (BSZ).

To study the therapeutic effects of Borsantrazole (BSZ) in the SOD1-G37R (42) mouse model of amyotrophic lateral sclerosis Protocol# 21-014, AC11693													
Control Treatment (N=12) including age and sex-matched animals (6 males and 6 females)							NS-1-2 Treatment (N=12) including age and sex-matched animals (6 males and 6 females)						
Animal ID	Sex	Life span/Age of survival (days)	Total number of IP,IJ	Age to reach disease onset (days)	Age to reach symptom onset (days)	Weight Loss (%) at humane end point	Animal ID	Sex	Life span/Age of survival (days)	Total number of IP,IJ	Age to reach disease onset (days)	Age to reach symptom onset (days)	Weight loss (%) at humane end point
R651RR	Male	195	106	131	180	Weight Loss>25%<br>Weight Loss (%): 25.324	R627R	Male	201	112	143	199	Weight Loss<25%<br>Weight Loss (%): 17.056
R644LL	Male	184	95	129	169	Weight Loss<25%<br>Weight Loss (%): 23.52	R711R	Male	201	112	143	171	Weight Loss<25%<br>Weight Loss (%): 18.91
R624R	Male	179	90	140	156	Weight Loss>25%<br>Weight Loss (%): 33.6	R715LR	Male	205	116	156	184	Weight Loss<25%<br>Weight Loss (%): 19.67
R712LL	Male	170	81	131	158	Weight Loss<25%<br>Weight Loss (%): 24.60	R736L	Male	189	100	147	180	Weight Loss<25%<br>Weight Loss (%): 20
R751L	Male	184	95	128	152	Weight Loss>25%<br>Weight Loss (%): 26.17	R739RR	Male	195	106	154	193	Weight Loss<25%<br>Weight Loss (%): 15.68
R738LL	Male	162	73	125	154	Weight Loss>25%<br>Weight Loss (%): 27.85	R635L	Male	182	93	168	182	Weight Loss<25%<br>Weight Loss (%): 18.306
R647LLR	Female	186	97	136	149	Weight Loss>25%<br>Weight Loss (%): 32.107	R638L	Female	198	109	164	195	Weight Loss<25%<br>Weight Loss (%): 18.543
R697LL	Female	187	98	106	163	Weight Loss>25%<br>Weight Loss (%): 26.923	R639R	Female	203	114	163	203	Weight Loss<25%<br>Weight Loss (%): 10.122
R699R	Female	198	109	131	186	Weight Loss<25%<br>Weight Loss (%): 23.58	R605L	Female	197	108	188	196	Weight Loss<25%<br>Weight Loss (%): 13.66
R705LR	Female	150	61	123	142	Weight Loss>25%<br>Weight Loss (%): 26.90	R700LR	Female	204	115	148	195	Weight Loss<25%<br>Weight Loss (%): 18.548
R703LL	Female	201	112	159	189	Weight Loss<25%<br>Weight Loss (%): 24.88	R701L	Female	174	85	144	165	Weight Loss<25%<br>Weight Loss (%): 23.34
R634LL	Female	139	50	117	127	Weight Loss>25%<br>Weight Loss (%): 30.081	R640LL	Female	167	78	144	160	Weight Loss>25%<br>Weight Loss (%): 25.64

Longitudinal Acute Toxicity Monitoring including age and sex-matched animals (6 males and 6 females)

NS-1-2 was originally developed in the lab. Later on, trademarked as Borsantrazole (BSZ).

Time(Days)	Wild type,Male(R608L) Control(Sham, 1:20,DMSO:PBS)	Wild type,Male(R614LL) Control(Sham, 1:20,DMSO:PBS)	Wild type,Male(R663R) Control(Sham, 1:20,DMSO:PBS)	Wild type,Male; Control(Sham, 1:20,DMSO:PBS) Mean Values	Wild type,Male(R609R) Treatment(10mg/kgbodyweight)	Wild type,Male(R612L) Treatment(10mg/kgbodyweight)	Wild type,Male(R662L) Treatment(10mg/kgbodyweight)	Wild type,Male; Treatment(10mg/kgbodyweight) Mean Values
1	23.9	24.8	27.9	25.53333333	24.9	25.2	28.9	26.33333333
2	24.3	25.1	27.3	25.56666667	25.5	25.2	28.2	26.3
3	24.4	24.8	27.5	25.56666667	25.5	25	27.9	26.13333333
4	23.7	24.7	27	25.13333333	25.4	25.3	27.6	26.1
5	23.8	25	27.3	25.36666667	25.6	25.1	28.3	26.33333333
6	23.9	25	26.9	25.26666667	25.4	25.6	27.9	26.3
7	23.9	24.4	26.9	25.06666667	25.6	25.2	28	26.26666667
8	23.3	25	27	25.1	25.3	25.6	28.1	26.33333333
9	23.5	24.6	26.5	24.86666667	25.6	25.2	28.6	26.46666667
10	24.3	25.1	26.1	25.16666667	26	25.1	29.1	26.73333333
11	23.8	24.5	26.5	24.93333333	25.5	25.5	29.5	26.83333333
12	24.3	25	26.6	25.3	26	25.2	29.8	27
13	24.4	24.8	26.6	25.26666667	26.3	25.4	30	27.23333333
14	24.3	24.9	27	25.4	26.7	25.8	30.4	27.63333333
**Percentage body weight change from baseline (initial weight)**	**1.673640167**	**0.403225806**	**-3.225806452**		**7.228915663**	**2.380952381**	**5.190311419**	

	**WG37R,Female; Control(Sham, 1:20,DMSO:PBS)**				**WG37R,Female; Treatment(10mg/kgbodyweight)**			
Time(Days)	Wild type,Female(R622R) Control(Sham, 1:20,DMSO:PBS)	Wild type,Female(R631LL) Control(Sham, 1:20,DMSO:PBS)	Wild type,Female(R661R) Control(Sham, 1:20,DMSO:PBS)	Wild type,Female; Control(Sham, 1:20,DMSO:PBS)Mean Values	Wild type,Female(R621L) Treatment(10mg/kgbodyweight)	Wild type,Female(R632L) Treatment(10mg/kgbodyweight)	Wild type,Female(R652L) Treatment(10mg/kgbodyweight)	Wild type,Female; Treatment(10mg/kgbodyweight) Mean Values
1	24.3	17.7	21.3	21.1	21.6	19.6	21.5	20.9
2	23.7	17.2	21	20.63333333	21.6	19.7	21.1	20.8
3	23.1	17.1	21.3	20.5	21.2	19.6	20.9	20.56666667
4	23.1	17	20.9	20.33333333	21.2	19.5	21.6	20.76666667
5	23.2	17.4	20	20.2	21.1	19.8	21.9	20.93333333
6	23.8	17.1	21.4	20.76666667	21.1	19.4	22	20.83333333
7	23.7	17.2	21.6	20.83333333	21.1	19.2	22.1	20.8
8	23.7	17	21.2	20.63333333	21.7	19	22.6	21.1
9	23	17.3	21	20.43333333	21.1	19.2	23	21.1
10	22.8	17.4	21.4	20.53333333	20.6	19.4	23.2	21.06666667

11	23.2	17.4	21	20.53333333	21.2	19.2	23.4	21.26666667
12	23	17.5	21.1	20.53333333	21.1	19.2	23.1	21.13333333
13	23	17.4	21.2	20.53333333	21.1	19	23.4	21.16666667
14	23.5	17.8	21	20.76666667	21.4	19.4	23.5	21.43333333
**Percentage body weight change from baseline (initial weight)**	**-3.29218107**	**0.564971751**	**-1.408450704**		**-0.925925926**	**-1.020408163**	**9.302325581**	
	**Wild type,Male; Control(Sham, 1:20,DMSO:PBS)**			**Wild type,Female; Control(Sham, 1:20,DMSO:PBS)**			**N=6**	
	**Wild type,Male(R608L) Control(Sham, 1:20,DMSO:PBS)**	**Wild type,Male(R614LL) Control(Sham, 1:20,DMSO:PBS)**	**Wild type,Male(R663R) Control(Sham, 1:20,DMSO:PBS)**	**Wild type,Female(R622R) Control(Sham, 1:20,DMSO:PBS)**	**Wild type,Female(R631LL) Control(Sham, 1:20,DMSO:PBS)**	**Wild type,Female(R661R) Control(Sham, 1:20,DMSO:PBS)**	**Wild type Control(Sham, 1:20,DMSO:PBS) Mean Values**	
	23.9	24.8	27.9	24.3	17.7	21.3	23.31666667	
	24.3	25.1	27.3	23.7	17.2	21	23.1	
	24.4	24.8	27.5	23.1	17.1	21.3	23.03333333	
	23.7	24.7	27	23.1	17	20.9	22.73333333	
	23.8	25	27.3	23.2	17.4	20	22.78333333	
	23.9	25	26.9	23.8	17.1	21.4	23.01666667	
	23.9	24.4	26.9	23.7	17.2	21.6	22.95	
	23.3	25	27	23.7	17	21.2	22.86666667	
	23.5	24.6	26.5	23	17.3	21	22.65	
	24.3	25.1	26.1	22.8	17.4	21.4	22.85	
	23.8	24.5	26.5	23.2	17.4	21	22.73333333	
	24.3	25	26.6	23	17.5	21.1	22.91666667	
	24.4	24.8	26.6	23	17.4	21.2	22.9	
	24.3	24.9	27	23.5	17.8	21	23.08333333	
	**Wild type,Male; Treatment(10mg/kgbodyweight)**			**Wild type,Female; Treatment(10mg/kgbodyweight)**			**N=6**	
	**Wild type,Male(R609R) Treatment(10mg/kgbodyweight)**	**Wild type,Male(R612L) Treatment(10mg/kgbodyweight)**	**Wild type,Male(R662L) Treatment(10mg/kgbodyweight)**	**Wild type,Female(R621L) Treatment(10mg/kgbodyweight)**	**Wild type,Female(R632L) Treatment(10mg/kgbodyweight)**	**Wild type,Female(R652L) Tteatment(10mg/kgbodyweight)**	**Wild type, Treatment(10mg/kgbodyweight) Mean Values**	
	24.9	25.2	28.9	21.6	19.6	21.5	23.61666667	
	25.5	25.2	28.2	21.6	19.7	21.1	23.55	
	25.5	25	27.9	21.2	19.6	20.9	23.35	
	25.4	25.3	27.6	21.2	19.5	21.6	23.43333333	
	25.6	25.1	28.3	21.1	19.8	21.9	23.63333333	
	25.4	25.6	27.9	21.1	19.4	22	23.56666667	
	25.6	25.2	28	21.1	19.2	22.1	23.53333333	
	25.3	25.6	28.1	21.7	19	22.6	23.71666667	
	25.6	25.2	28.6	21.1	19.2	23	23.78333333	

26	25.1	29.1	20.6	19.4	23.2	23.9
25.5	25.5	29.5	21.2	19.2	23.4	24.05
26	25.2	29.8	21.1	19.2	23.1	24.06666667
26.3	25.4	30	21.1	19	23.4	24.2
26.7	25.8	30.4	21.4	19.4	23.5	24.53333333

	**Percentage body weight change from baseline (initial weight) Based on initial(day 1) and end of study(day 120)**	
	**Control Wildtype**	**Treatment Wildtype**
	1.673640167	7.228915663
	0.403225806	2.380952381
	-3.225806452	5.190311419
	-3.29218107	-0.925925926
	0.564971751	-1.020408163
	-1.408450704	9.302325581
**Percentage mean change**	**-0.88076675**	**3.692695159**

Longitudinal Chronic Toxicity Monitoring including age and sex-matched animals (5 males and 6 females) NS-1-2 was originally developed in the lab. Later on, trademarked as Borsantrazole (BSZ).

		Wild type,Male; Control(Sham, 1:20,DMSO:PBS)			Wild type,Male; Treatment(10mg/kgbodyweight)			Wild type,Female; Control(Sham, 1:20,DMSO:PBS)			Wild type,Female; NS-1-2(10mg/kgbodyweight)			N=5	N=6
	DOB	09-12-2022	09-12-2022	26-10-2022	07-08-2022	30-10-2022	09-12-2022	26-10-2022	09-09-2021	30-10-2022	30-10-2022	09-12-2022	26-10-2022		
Age of Mice	Time(Treatment Days)	Wild type,Male(R724L) Control(Sham, 1:20,DMSO:PBS)	Wild type,Male(R722R) Control(Sham, 1:20,DMSO:PBS)	Wild type,Male(R710R) Control(Sham, 1:20,DMSO:PBS)	Wild type,Male(R694R) Treatment(10mg/kgbodyweight)	Wild type,Male(R714R) Treatment(10mg/kgbodyweight)	Wild type,Male(R723LL) Treatment(10mg/kgbodyweight)	Wild type,Female(R707L) Control(Sham, 1:20,DMSO:PBS)	Wild type,Female(R607LL) Control(Sham, 1:20,DMSO:PBS)	Wild type,Female(R704RR) Control(Sham, 1:20,DMSO:PBS)	Wild type,Female(R706LLR) Treatment(10mg/kgbodyweight)	Wild type,Female(R718L) Treatment(10mg/kgbodyweight)	Wild type,Female(R708R) Treatment(10mg/kgbodyweight)	Wild type Control(Sham, 1:20,DMSO:PBS) Mean Values	Wild type, Treatment(10mg/kgbodyweight) Mean Values
90	1	25.8	25.3	ID,710R: The mice were	31.9	23.5	28	24.3	22.8	20.5	21.5	26.1	23.5	23.74	25.75
91	2	25.2	26.2		31.9	23.5	28.1	24.5	22.9	20.5	21.6	25.6	23.6	23.86	25.71666667
92	3	25.1	26.3		31.9	23.6	28.2	24.2	22.2	20.6	21.6	24.8	23.5	23.68	25.6
93	4	25.4	26.4		31.7	23.8	28.4	24.3	22.3	20.7	21.7	25.3	23.6	23.82	25.75
94	5	25.5	26.6		31.7	23.9	29	24.3	22.8	20.8	21.6	25.3	23.7	24	25.86666667
95	6	25.5	26.6		31.6	23.9	28.6	24.2	22.7	20.9	21.7	25.6	23.6	23.96	25.83333333
96	7	25.5	26.6		31.5	24	28.6	24.2	22.5	21	21.8	25.6	23.6	23.96	25.85
97	8	25.6	25.7		31.5	24.3	28.8	24	22.8	21.2	21.9	25.2	23.7	23.86	25.9
98	9	25.3	25.7		31.5	24.5	28.8	24.1	22.7	21.4	22	26.2	23.8	23.84	26.13333333
99	10	25.2	25.9		31.5	24.3	29.2	24	22.4	21.5	22.1	25	23.9	23.8	26
100	11	26	26.1		31.6	25.2	28.8	23.8	22.7	21	22.5	25.1	23.7	23.92	26.15
101	12	25.5	27		31.6	25	30	23.6	22.6	21.2	22.1	25.5	23.6	23.98	26.3
102	13	25.6	26.7		31.6	24.5	29	23.4	22.5	20.7	21.7	26.4	23.7	23.78	26.15
103	14	25.6	26.7		31.5	24	29.5	23.4	22.5	20.3	21.5	25.8	23.8	23.7	26.01666667
104	15	25.8	26.6		31.7	23.8	29.4	23.4	22.4	20.5	21.3	26.2	23.8	23.74	26.03333333
105	16	24.6	27		31.7	23.5	29.7	23.8	22.8	20.2	21.2	26.8	23.4	23.68	26.05
106	17	25	27.3		31.9	23.3	30.5	23.9	22.8	20.1	21.3	27.2	23.2	23.82	26.23333333
107	18	26	28		31.9	23	31	23.8	22.5	20.3	21.3	27.8	23.3	24.12	26.38333333
108	19	25.8	28.1		31.9	23.1	31.5	23.9	23	20.4	21.2	27.8	23.4	24.24	26.48333333
109	20	26	27.9		32.1	23	32	24.1	22.4	20.1	21.6	27.7	23.5	24.1	26.65
110	21	25.7	28		32	22.8	31.7	24.2	22.7	20.2	21.4	28.6	23.6	24.16	26.68333333
111	22	25.3	27.8		32.1	22.5	32.1	24.5	22.5	20.3	21	29.2	23.1	24.08	26.66666667
112	23	25.3	27.9		32.1	22.4	32.1	24.6	22.2	20.3	21	29.2	22.9	24.06	26.61666667
113	24	25.3	27.9		32.1	22.6	31.9	24.9	22.1	20.4	21.5	29	23	24.12	26.68333333
114	25	25.2	28.9		32.2	22.4	32	25.1	22.5	19.3	21.5	29.5	23.1	24.2	26.78333333
115	26	25.3	28.1		32.6	22.9	32	24.8	22.8	19.4	21.8	29.3	23.2	24.08	26.96666667
116	27	25.8	28.8		32.7	23.2	32	24.8	22.5	20.5	21.3	30.3	23.5	24.48	27.16666667
117	28	25.8	28.8		32.9	22.9	32.4	24.9	22.7	19.8	21.8	30.5	23.6	24.4	27.35
118	29	26.1	29		32.9	22.9	32.9	25.2	22.6	19.7	22	31	23.4	24.52	27.51666667
119	30	26	29.1		33.2	23.1	32.8	25.4	22.7	19.6	21.6	31	23.9	24.56	27.6
120	31	25.5	28.7		33.5	22.4	32.8	24.7	23.1	20.2	21	29.1	23.4	24.44	27.03333333
121	32	26.2	28.6		33.6	22.1	32.7	25.2	22.7	19.7	21.5	29.7	23.9	24.48	27.25
122	33	26.1	29.3		33.7	21.8	33	25.1	23.5	19.3	22.2	30.2	24.2	24.66	27.51666667
123	34	26.2	29.1		33.8	22.4	32.9	25	23.1	20	22.3	30.2	23.9	24.68	27.58333333
124	35	26.3	29.4		33.9	22.4	32.9	25.4	23.3	20.5	21.6	30.1	23.4	24.98	27.38333333
125	36	25.8	29		34	22.5	32.3	25.2	23.6	19.7	21.5	29.6	23.7	24.66	27.26666667
126	37	26.1	29.4		34.2	23.1	32.3	24.5	23.6	20	21.6	30.1	23.8	24.72	27.51666667
127	38	26.2	29.3		34	23	32.2	24.6	23.2	20	21.6	30.2	23.5	24.66	27.41666667
128	39	26.4	29.5		34.2	23.1	32.4	24.8	24.3	20	21.5	30.1	23.5	25	27.46666667
129	40	25.8	29.3		34.3	23.2	32.4	25.5	24	20.3	21.4	29.2	23.7	24.98	27.36666667
130	41	26.1	29.5		34.4	22.7	32.3	25.7	24.1	19.7	22.5	29.3	23.5	25.02	27.45
131	42	26.2	30.3		34.5	22.9	32.5	25.8	23.8	20.6	22.4	29.8	23.2	25.34	27.55
132	43	26.1	29.2		34.6	22.9	32.7	25.7	24	20.4	22.6	29.3	23.2	25.08	27.55
133	44	26.8	29.7		34.7	22.8	33.2	25	24	20.5	22.8	30.8	23.4	25.2	27.95
134	45	26.3	29.5		34.8	22.5	33.6	25.1	24.3	20.7	22.6	30.5	23.5	25.18	27.91666667
135	46	27.1	30.4		35	23.2	33.5	25.2	24.1	20.2	23	30.7	23.7	25.4	28.18333333
136	47	27	31.5		35	23.3	34.9	25.3	23.8	20.3	23.1	30.5	23.5	25.58	28.38333333
137	48	27.1	31.5		35.2	23.6	35	25.6	23.5	20.8	23.7	30.6	23.2	25.7	28.55
138	49	27	31.3		35.3	23.4	34.7	25.5	23.3	20.8	22.9	30.7	23.3	25.58	28.38333333
139	50	26.2	30.7		35.4	22.6	35.2	25.1	23.7	20.6	22.8	31	24.2	25.26	28.53333333
140	51	25.9	31		35.4	22.8	34.6	25.2	24	20.8	22.8	30.8	23.5	25.38	28.31666667
141	52	25.8	31.1		35.6	23.1	34.8	26.1	23.6	20.8	23.2	30.3	23.5	25.48	28.41666667
142	53	27.2	32.1		35.7	23.1	35.2	25.8	24	21.5	23.3	30.9	23.7	26.12	28.65
143	54	27.2	32		35.8	23	35.2	25.9	23.9	20.7	23	31.7	23.8	25.94	28.75
144	55	27.3	32.1		35.8	23.1	35.2	26.3	24	20.7	23.7	31.5	23.5	26.08	28.8
145	56	25.9	31.9		35.8	23.5	35.3	25.8	24	20.7	23.9	30.5	23.2	25.66	28.7
146	57	25.9	31.6		35.8	24	35	25.1	23.7	20.7	24	29.9	23.7	25.4	28.73333333
147	58	26.7	32.1		35.9	24.1	35.3	25.2	23.5	20.8	23	31.3	24.2	25.66	28.96666667
148	59	26.2	31.4		36	24.5	34.9	25.6	24	20.7	23.5	30.6	24.3	25.58	28.96666667
149	60	26.6	32.7		36.1	24.9	35.8	26	24	20.7	22.9	31.2	24.5	26	29.23333333
150	61	26.9	33.1		36.2	24.9	35.8	25.5	23.4	20.7	23.5	31	23.9	25.92	29.21666667
151	62	26.9	33.3		36.3	24.9	35.8	26.4	23.7	20.1	23.5	31	23.8	26.08	29.21666667
152	63	26.5	33.2		36.4	25.1	36	26.2	24.1	20.4	23.5	31.2	24	26.08	29.36666667
153	64	26.9	33.9		36.5	25.2	37	26	24.1	21.3	23.6	32	24.5	26.44	29.8
154	65	26.9	33.7		36.8	25	36.6	26.3	24	20.7	23.5	31.2	25	26.32	29.68333333
155	66	26.4	33.8		36.8	25.2	36.8	26	24.2	21	23.3	32	25	26.28	29.85
156	67	26.8	33.6		37	25.2	36.8	26.9	25	20.6	23.3	31.2	24.7	26.58	29.7
157	68	26.9	33.2		37.2	25	36.3	26	25.2	20.6	23.7	31	24.9	26.38	29.68333333
158	69	26.9	33		37.3	24.6	36.1	26.1	25	20	23.7	31.1	24.9	26.2	29.61666667
159	70	26.6	33.9		37.4	24.5	37.8	26.1	25	20.1	23.6	30.9	25.5	26.34	29.95
160	71	26.4	33.3		37.5	24.7	37.9	25	25.1	20.7	23.9	31	25.9	26.1	30.15
161	72	26.2	33.8		37.6	25	37.5	25.4	25.4	20.6	23.9	30.7	25.8	26.28	30.08333333
162	73	26.2	33.3		37.8	24.8	37.2	25.8	26.1	20.7	23.8	31.7	25.8	26.42	30.18333333
163	74	27	35.3		37.9	24.8	38	25.6	26.1	20.6	24.3	31.7	25.7	26.92	30.4
164	75	27.5	35.2		38	25	38	26.3	26.2	21.2	23.3	32.1	25.2	27.28	30.26666667
165	76	27.5	35.1		38.1	24.9	37.9	26.5	26.1	20.3	23.7	31.7	25.7	27.1	30.33333333
166	77	27.7	35.1		38.2	25	38	26.7	26.6	20.6	23.8	31.5	25.3	27.34	30.3
167	78	27.4	35.5		38.3	25.2	38.4	26.5	27	20.5	23.7	31.9	25.5	27.38	30.5
168	79	27.9	35.9		38.4	25.1	38.5	26.5	27	20.7	23.5	32.9	24.7	27.6	30.51666667
169	80	27.3	35.8		38.4	24.6	38.1	26.9	27	20.3	23.7	32.4	24.3	27.46	30.25
170	81	28.4	37		38.5	24.6	38.3	26.5	27.1	20.5	24.8	32.7	25.7	27.9	30.76666667
171	82	28.3	37.1		38.7	25	38.6	26.3	27.2	21.3	24.2	32.5	25.4	28.04	30.73333333
172	83	28	36.7		38.8	24.8	37.8	26.6	27.2	21.5	24.2	32.2	26.8	28	30.76666667
173	84	28.1	37.8		39	25.2	38.3	26.2	27.4	21.7	24.3	32.2	26.3	28.24	30.88333333
174	85	28.7	37.5		39.1	25.2	38.2	26.3	27.4	21.3	24.6	32.4	26	28.24	30.91666667
175	86	28.3	36.7		39.1	25.1	37.6	27.6	27.8	21.6	24.3	32.4	26.2	28.4	30.78333333
176	87	28.7	36.3		39.5	25.3	37.6	27.2	28	21.9	24.3	32.4	26.9	28.42	31
177	88	29.2	37.7		39.7	25.2	38.1	27.2	28.2	21.6	24.6	32.7	26.7	28.78	31.16666667
178	89	28.9	37.7		39.9	25.3	37.5	28.1	28.6	21.7	23.9	32.8	26.8	29	31.03333333
179	90	28.7	37.7		40.1	25.7	37.9	28.3	29	21.7	23.5	32.4	27.1	29.08	31.11666667
180	91	28.6	37.5		40.3	24.7	37.9	28.3	29.1	21.6	24	31.7	27.5	29.02	31.01666667

181	92	28.9	37.4		40.5	25.2	37.5	28.1	29.7	22.1	24.2	31.6	27.3	29.24	31.05
182	93	29	38.1		40.8	25.7	38.3	28.3	30	21.9	24.1	32.3	26.5	29.46	31.28333333
183	94	28.9	37.9		41	26.1	38	28.5	30.5	22.3	24.2	32.6	26.3	29.62	31.36666667
184	95	28.8	37.9		41.1	26.1	37.9	27.9	30.9	22.3	23.8	32.5	27.4	29.56	31.46666667
185	96	28.9	37.7		41.3	25.1	37.7	28.8	31.4	21.6	23.4	32.3	26.8	29.68	31.1
186	97	29.1	37.5		41.4	25	37.9	29.7	32	21	23.4	32.2	27	29.86	31.15
187	98	29.3	38.7		41	25.8	38.6	29.6	32.5	21.4	23.8	32	27.1	30.3	31.38333333
188	99	29	38.6		41.3	25.2	38.2	29.7	32.9	21.5	24.2	31.7	27.2	30.34	31.3
189	100	28.5	38.3		41.5	25.7	37.4	28.2	33.1	21.7	24.2	32.5	27.8	29.96	31.51666667
190	101	28.4	37.9		41.6	25.2	36.3	28.6	33.4	21.7	24.4	32	28.2	30	31.28333333
191	102	29.2	38.8		41.7	26.1	37.5	28.3	33.5	22.1	24.8	31.4	27.6	30.38	31.51666667
192	103	29.6	39		41.8	26	37.6	28.3	33.8	21.6	25.3	31.6	27.5	30.46	31.63333333
193	104	29.1	38.8		42.1	26.3	37.5	28.1	34.1	22.6	25.7	31.5	27.8	30.54	31.81666667
194	105	29.5	38.9		42.1	26.4	38	28	34.2	22.9	25.6	31	27.3	30.7	31.73333333
195	106	29.8	39.5		42.2	26.3	38.1	28.9	34.6	22.6	25.6	31.8	27.4	31.08	31.9
196	107	29.4	39.5		42.3	26.4	37.6	28.8	34.9	22.7	26.2	31.9	27.3	31.06	31.95
197	108	28.6	38.8		42.3	26	37.2	29.5	35	22.3	25	31.7	28.2	30.84	31.73333333
198	109	28.9	39.7		42.4	26.2	38.3	29.6	35.4	22.4	24.7	31.8	28	31.2	31.9
199	110	29	40.2		42.5	25.5	38.2	29.5	35	21.9	25.6	31.2	28.1	31.12	31.85
200	111	28.9	39.5		42.6	26.7	38.2	29.6	35.1	22.6	25	31.9	28	31.14	32.06666667
201	112	29	40.5		42.8	25.7	38.7	29.4	34.5	22.1	24.6	32	27.9	31.1	31.95
202	113	29.1	41.2		43	25.4	39.8	29.5	33.8	21.6	25.1	32.3	28.2	31.04	32.3
203	114	28.9	39.9		43.2	26.4	37.9	29.1	33.4	21.6	25.2	32.8	28.6	30.58	32.35
204	115	28.4	40.7		43.2	26.5	37.9	30.7	33.3	21.6	25.2	32.9	28.4	30.94	32.35
205	116	29	40.4		43.4	26.4	38.1	30.1	33	22.6	26	32.4	29	31.02	32.55
206	117	28.8	40.5		43.4	26.9	38.5	29.8	32.9	22.5	25.6	32.5	28.7	30.9	32.6
207	118	28.6	40.9		43.5	26.3	39.3	30.4	32.2	22.3	26.1	33.1	29	30.88	32.88333333
208	119	28.8	40.5		43.5	26.7	39.1	30.1	32.4	22.2	25.7	32.3	29.2	30.8	32.75
209	120	28	40.4		43.8	27	39.1	30	32.3	22.3	26.2	33.5	29.6	30.6	33.2
**Percentage body weight change from baseline (initial weight) Based on initial(day 1) and end**		**8.527131783**	**59.68379447**		**37.30407524**	**14.89361702**	**39.64285714**	**23.45679012**	**41.66666667**	**8.780487805**	**21.86046512**	**28.35249042**	**25.95744681**		

	**Percentage body weight change from baseline (initial weight) Based on initial(day 1) and end of study(day 120)**	
	**Control Wildtype**	**Treatment Wildtype**
	8.527131783	37.30407524
	59.68379447	14.89361702
	23.45679012	39.64285714
	41.66666667	21.86046512
	8.780487805	28.35249042
		25.95744681
**Percentage mean change**	**28.42297417**	**28.00182529**

Longitudinal Survival Monitoring

NS-1-2 was originally developed in the lab. Later on, trademarked as Borsantrazole (BSZ).

BSZ Treatment (N=12) including age and sex-matched animals (6 males and 6 females)

Control Treatment (N=12) including age and sex-matched (6 r

	Sex	Female	Female	Female	Male	Male	Female	Female	Female	Male	Male	Male	Male	Female	Female
	Animal ID:	R605L	R639R	R638L	R627R	R635L	R700LR	R640LL	R701L	R711R	R715LR	R736L	R739RR	R647LL(R)	R634LL
	Time(Treatment Days)	HetG37R,Female(R605L) BSZ(10mg/kgbodyweight)	HetG37R,Female(R639R) BSZ(10mg/kgbodyweight)	HetG37R,Female(R638L) BSZ(10mg/kgbodyweight)	HetG37R,male(R627R) BSZ(10mg/kgbodyweight)	HetG37R,male(R635L) BSZ(10mg/kgbodyweight)	HetG37R,female(R700LR) BSZ(10mg/kgbodyweight)	HetG37R,female(R640LL) BSZ(10mg/kgbodyweight)	HetG37R,female(R701L) BSZ(10mg/kgbodyweight)	HetG37R,male(R711R) BSZ(10mg/kgbodyweight)	HetG37R,male(R715LR) BSZ(10mg/kgbodyweight)	HetG37R,male(R736L) BSZ(10mg/kgbodyweight)	HetG37R,male(R739RR) BSZ(10mg/kgbodyweight)	HetG37R,female(R647LL(R)) Sham treatment (1:20 DMSO/PBS)	HetG37R,female(R634LL) Sham treatment (1:20 DMSO/PBS)
1	90 (First dose)	23	22.6	23.3	33.7	27	24	23.3	20.5	28.7	27.1	29.1	29.1	23.5	23.4
2	91	23.6	23	23.6	33.3	27.6	24	23.9	20.4	28.8	27.3	29.2	29.3	23.4	23
3	92	23	23	23.3	33.3	28	24	24.2	20.5	28.6	27.5	29.3	29.4	23.8	23
4	93	22.8	23.1	23.4	33.2	28.1	24.1	24.2	20.4	28.9	27.6	29.5	29.2	23.2	22.7
5	94	23	23.2	23.5	33.5	28.2	24.2	24.3	20.5	28.5	27.8	29.6	29.3	23	22.5
6	95	23	23.8	24.1	33.4	27.7	24	24.7	20.5	28.6	27.5	29.6	29.3	23.1	22.6
7	96	23.3	23.8	24.1	33.5	27.9	23.9	24.8	20.6	28.6	27.6	30	29.7	23	22.6
8	97	23.2	23.8	24.1	33.4	28	23.9	24.8	20.8	28.9	27.8	30.2	29.9	23.1	22.6
9	98	23.2	23.9	24.1	33.8	28.1	24	24.8	21	28.8	27.7	30.5	30.2	23	22.7
10	99	23.1	23.5	23.5	33.7	28	24.1	24.6	21.1	28.7	27.6	30.5	30.4	23	22.6
11	100	23.1	23.6	23.6	34	28.2	24	24.6	21	28.9	27.5	30.6	30.3	23.6	22.7
12	101	23.8	24	23.9	34.1	28.5	23.9	25	21.2	29.1	27.8	30.9	30.5	23.4	23.1
13	102	23.8	24.1	23.8	34.2	28.7	23.9	25.1	21.3	29	27.5	31	30.6	23.1	23.2
14	103	23.7	24.3	23.7	34.4	29.3	24	25.2	21	28.9	27.5	31	30.3	23.3	22.8
15	104	23.6	24.5	24	34.9	29.2	23.9	25.9	21	28.5	27.3	30.6	30.5	23.4	23
16	105	24	24.6	24.2	35.1	29.4	23.9	25.9	20.9	28.6	27	30.6	30.6	23.4	23.2
17	106	24.2	24.4	24	35.6	29.4	23.7	26.2	20.9	28.5	26.9	30.6	30.9	23.1	23.6
18	107	24.4	24.5	24.3	36	29.5	23.8	26.3	20.8	28.6	26.9	30.3	30.9	23.3	23.7
19	108	24.9	24.6	24.4	36.4	29.8	23.9	26.4	20.9	28.5	26.8	30.1	30.7	23.7	23.6
20	109	24.8	24.6	24.5	36.8	29.9	23.9	26.5	20.9	28.6	27.1	30.2	30.6	23.8	23.9
21	110	24.9	24.5	24	37	30.6	23.9	27	20.2	28.5	27.5	30.4	30.8	23.6	24.2
22	111	24.4	24.7	24.4	37.1	31	23.8	27.3	20.2	28.3	27.4	30.8	30.9	24.4	24.3
23	112	24.5	24.9	24.6	37.5	31.4	23.8	27.6	20.3	28	27.5	30.2	31	24.5	24.5
24	113	24.7	25.3	24.3	37.8	31.8	23.9	27.3	20.6	28.2	27.6	30.9	31.3	24.6	24.5
25	114	24.8	25.6	24.5	37.8	32.1	23.9	27.7	20.6	28.4	27.7	30.6	31	24.6	24.3
26	115	25.1	25.9	24.8	37.9	32.6	23.9	28	21	28	27.8	30.6	30.8	24.2	24.5
27	116	24.8	26.2	25.3	37.1	32.7	23.9	28.2	21.1	28	28.1	30.1	31.1	24.7	24.5
28	117	25.2	26.4	25.6	37.6	32.5	23.8	28.5	21.4	28.1	28.4	30	31	25.1	24.6(Maximum Weight)
29	118	25.1	26.1	25.1	38	31.9	23.8	28.8	21.5	28.2	28.6	29.9	31.2	25.4	24
30	119	25.6	26.3	25.3	38.2	32.3	23.9	29.1	21.6	28.3	28.7	30	31.5	25.8	23.7
31	120	25.4	26.5	25.6	38.6	32.5	23.8	29	21.6	28.6	28.5	30.3	31.6	26.1	23.9
32	121	25.7	26.9	25.8	39	32.9	23.8	29.4	21.7	28.7	28.5	30.6	31.8	26.4	23.6
33	122	25.8	27.3	26.1	39.3	33.3	23.9	29.8	21.8	28.9	28.5	31	31.9	26.5	23.6
34	123	26	27.6	26.4	39.3	33.5	23.9	30	21.8	28.9	28.6	31.2	31.7	26.8	23.6
35	124	25.6	28	26.8	39.6	33.8	23.9	30.4	21.4	28.4	29	31.5	31.7	27.1	23.3
36	125	25.8	28.5	26.2	39.9	34.2	23.9	30.8	21.5	28	29.1	31.5	31.1	27.2	22.8
37	126	25.8	28.6	26.5	39.8	34.3	23.9	30.9	21.6	28.3	29.4	31.4	31	27.6	22.7
38	127	25.5	28.8	26.7	40	34.3	23.9	31	21.6	28.2	29.5	31	31	27.8	22.7(10%Reduction)
39	128	25.3	28.8	26.1	40.3	34.3	24	31.4	21.5	28.5	29.5	30.8	30.9	27.9	21.9
40	129	25.6	29.2	26	40.6	34.2	24	31.8	21.4	28.6	29.5	30.6	31.1	28.4	21.3
41	130	25.3	29.5	26.1	40.8	34.6	24	32.1	21.4	28.7	29.5	30.5	31.2	28.8	20.9
42	131	25.1	29.8	26.1	41	34.4	23.9	32.6	21.7	29.1	29.4	30.2	31.6	29.1	20.6
43	132	25.1	29.9	26.4	41.2	34.4	23.9	32.7	21.6	29.2	29.5	30.1	31.6	29.3	20.3
44	133	25	30	26.6	41.2	34.5	24	33.1	21.8	28.9	29.3	30	31.4	29.5	19.9
45	134	25.3	30	26.6	41.3	34	24.1	33.1	21.9	28.9	29.4		31.8	29.6	19
46	135	25.2	30	26	41.2	34.4	24.1	33.5	21.8	28.8	29.1	31	31.7	29.4	19.4
47	136	25.2	30.1	26.5	41.4	34.4	24.1	33.5	21.6	28.9	29.6	30.4	32	29.9(Maximum Weight)	19
48	137	25.1	30.2	26.9	41.3	34.8	24.1	33.8	21.7	28.9	29.8	30.1	32	29.1	18.8
49	138	25.4	30.6	27.1	41.5	34.8	24.1	34.1	22	28.9	29.9	30.1	32.1	29.2	18.3
50	139	25.7	30.6	27.4	41.8	34.8	24.2	34.3	22.2	28.8	29.8	30	32	28.6	17.3(Humane End Point)
51	140	26.3	30.4	27.1	41.9	35	24.2	34.2	22.4	28.7	30	30.3	32.3	28.8	
52	141	25.7	30.5	27.2	42.3	35.1	24.2	34	22.5	29	30.1	30	32.4	28.1	
53	142	25.6	30.3	27.4	42.5	35	24	34.1	22.5	29.4	29.9	31.1	32.4	27.8	
54	143	25.6	30.5	27.6	42.8(Maximum Weight)	34.8	24.2	34.3	22.7	29.6(Maximum Weight)	30.2	30.4	32.4	27.3	
55	144	26	30.7	27.9	42.6	35	24.3	34.7(Maximum Weight)	22.7(Maximum Weight)	29.1	30.2	29.8	32.6	27.4	
56	145	26.1	31	27.8	42	35.2	24.5	34.4	22.6	28.7	30.1	31.1	32.5	27.4	
57	146	26.5	31.2	27.9	41.8	35.4	24.6	34.5	22.2	28.5	29.9	31	32.8	27.5	
58	147	26.1	31.3	27.9	41.8	35.1	24.7	34.5	22.1	28.6	29.8	31.5(Maximum Weight)	32.7	27.1	
59	148	26.3	31.4	27.9	41.8	35.3	24.8(Maximum Weight)	34.6	22.6	28.6	29.9	31.4	32.6	27	
60	149	26	31.5	28	41.9	35.4	24.8	34.6	22.4	28.2	30	30.1	32.7	26.9 (10%Reduction)	
61	150	26	31.6	28	42	35.2	24.8	34.5	22.6	28	30	30.1	32.8	26.8	
62	151	25.9	31.6	28.2	41.8	35.1	24.8	34.5	22.1	28.1	30.1	29.9	32.8	26.5	
63	152	26.1	31.5	28	42.2	35	24.6	34	21.7	27.8	30	30.9	33.1	26.8	
64	153	26.1	31.3	28.1	42.2	35.2	24.5	34.1	21.6	27.7	30.2	30.2	33.4	26.5	
65	154	26.2	31.5	28.3	42	35.6	24.7	33.8	21.6	27.6	30.3	30.1	33.8(Maximum Weight)	26	
66	155	25.6	31.4	28.2	42	35.5	24.7	33.6	21.2	27.7	30.4	30.6	33.5	25.9	
67	156	26.2	31.8	28.6	42.1	35.5	24.6	33.2	21.3	27.5	30.5(Maximum Weight)	29.3	33.2	25.6	
68	157	26.3	31.9	29	41.8	35.6	24.5	32.7	21.1	27.6	30.2	29.4	33	25.6	
69	158	26.4	31.9	29.3	41.8	35.8	24.2	32.2	21.3	27.1	30.1	29	33	25.9	
70	159	26.4	32	29.5	42	35.9	24.2	31.6	21.4	27	30.2	29.9	33.1	25.8	
71	160	26.5	32.3	29.6	42.1	36.2	24.1	31(10%Reduction)	21.1	27.2	30	29.9	33	25.7	
72	161	26.8	32.4	29.8	42.1	36.1	24.1	30.5	21	27.2	29.8	29.9	32.5	25.9	
73	162	27.1	32.5	29.9	42	35.8	24.3	30	21	27.6	30	29.8	32.9	25.7	
74	163	27.2	32.6(Maximum Weight)	30	42	36.1	24	29.1	20.9	27.3	30	30.1	32.3	25.5	
75	164	27.5	32.5	30.2(Maximum Weight)	42.2	36.3	24.1	28.2	20.9	27	29.6	30.2	32	25.2	
76	165	27.4	32.1	29.9	42.1	36.5	24.1	27.8	20.5(10%Reduction)	27.6	28.6	30.1	31.8	25	
77	166	27.1	31.6	29.6	41.8	36.6	24.3	26.8	20.3	27.8	28.6	30.1	31.8	24.8	
78	167	27.4	31.2	29.2	42	36.5	24.3	25.8(Humane End Point)	19.8	27.4	28.5	30.5	31.6	25	
79	168	27.1	31.5	29.3	41.3	36.6(Maximum Weight)	23.9		19.8	26.5	28.5	30.6	31.2	24.5	
80	169	27	31.7	29.4	41.1	36.2	23.8		19.8	25.8	28.9	31	31.6	24.3	
81	170	27	31.7	29.2	41.3	36.3	24.1		19	26.1	29.3	30.5	31.6	23.9	
82	171	26.7	31.5	28.8	41.6	36	24		18.6	26.3(10%Reduction)	29.5	30	31.7	23.6	
83	172	26.5	31.4	28.8	41.4	36	24		18.1	26.8	28.8	29.8	31.8	23.6	
84	173	26.2	31.3	28.7	41.4	35.8	23.7		18	26.9	28	29.6	31.8	23.7	
85	174	25.7	31.2	28.6	41.2	35.5	23.5		17.4(Humane End Point)	26.2	28.2	29.3	32	23.4	
86	175	25.4	31.3	28.8	41.2	35.4	23.6			26.2	28	29	32.2	23.1	
87	176	25.5	31	28.8	41	35.6	23.9			26	27.7	29.3	32.5	23.1	
88	177	25.8	31.2	29.9	40.8	34.9	23.3			26	27.7	29.3	32.7	23	
89	178	26.2	31	29.2	41	34.2	23.4			25.9	27.6	29.1	32.6	22.9	
90	179	26.8	31.1	29.3	41.3	33	23.2			26	27.5	29.1	32.6	22.7	
91	180	26.8	30.9	29.5	41.4	31.9	23.4			25.8	27.5	28.5(10%Reduction)	31.9	22.4	
92	181	27.1	30.9	29.4	41.5	30.5	23.1			25.8	27.6	28	31.7	22.2	
93	182	27.2	30.5	29	41.3	29.9(Humane End Point) (10%Reduction)	23.1			25.6	27.5	28	31.7	21.8	
94	183	26.9	30.3	28.9	41.2		23.5			26	27.4	27.7	31.7	21.5	
95	184	27.2	30.3	28.4	41.4		23.7			26	27.4(10%Reduction)	27.4	31.5	21.4	
96	185	27.5	30.4	28.3	41.5		23.9			25.8	27	27.6	31.6	21.2	
97	186	27.5	30.8	29.1	41.3		23.7			25.1	26.9	27	31.5	20.3(Humane End Point)	
98	187	27.7	31.2	28.9	41.3		23.5			25.2	27.3	26.9	31.5		
99	188	27.8(Maximum Weight)	31.5	29.1	41.4		23.6			25.3	27.3	25.9	31.7		
100	189	27.4	31.5	29	41.3		23.4			25	27.6	25.2(Humane End Point)	31		
101	190	27.2	31.7	28.8	39.9		23.5			25.3	28		31		
102	191	27.4	31.6	28.5	40.8		22.9			25.3	27.9		30.6		
103	192	27.5	31.4	28.3	41		23			25	28.2		30.5		
104	193	27.1	31.6	28.4	41.2		22.9			25.3	28.6		30.4(10%Reduction)		
105	194	27.6	31.8	28.1	40.5		22.5			25	29		30		
106	195	26.3	31.5	27.6(10%Reduction)	40.2		22(10%Reduction)			25	28.9		28.5(Humane End Point)		
107	196	25.1 (10%Reduction)	31.7	27	40.1		21.8			25.2	28.8				
108	197	24 (Humane End Point)	31.7	25.8	40		22.4			25.2	29				
109	198		31.5	24.6(Humane End Point)	40.1		22			25.2	28.4				
110	199		31.4		38.1(10%Reduction)		22			25.3	28				
111	200		30.7		36.3		22			25	27.8				
112	201		30.4		35.5(Humane End Point)		21.9			24(Humane End Point)	27.3				
113	202		30.1				21.5				26.8				
114	203		29.3 (Humane End Point) (10%Reduction)				21.2				26.6				
115	204						20.2(Humane End Point)				25.5				
116	205										24.5(Humane End Point)				
117	206														

males and 6 females)

Male	Male	Male	Male	Male	Male	Female	Female	Female	Female
R712LL	R738LL	R751L	R644LL	R651RR	R624R	R697LL	R699R	R705LR	R703LL
HetG37R,male(R712LL)<br>Sham treatment<br>(1:20 DMSO/PBS)	HetG37R,male(R738LL)<br>Sham treatment<br>(1:20 DMSO/PBS)	HetG37R,male(R751L)<br>Sham treatment<br>(1:20 DMSO/PBS)	HetG37R,male(R644LL)<br>Sham treatment<br>(1:20 DMSO/PBS)	HetG37R,male(R651RR)<br>Sham treatment<br>(1:20 DMSO/PBS)	HetG37R,male(R624R)<br>Sham treatment<br>(1:20 DMSO/PBS)	HetG37R,Female(R697LL)<br>Control(Sham, 1:20,DMSO:PBS)	HetG37R,Female(R699R)<br>Control(Sham, 1:20,DMSO:PBS)	HetG37R,Female(R705LR)<br>Control(Sham, 1:20,DMSO:PBS)	HetG37R,Female(R703LL)<br>Control(Sham, 1:20,DMSO:PBS)
29.5	30.3	29	30.8	29.2	30.4	23.1	22.5	20.9	20.4
29.7	30.4	29.3	30.4	28.8	30	23.1	22.5	20.8	20.5
29.7	30.2	29.5	30	29.2	30.2	23.1	22.5	21	20.4
29.6	29.8	29.8	30.1	29	30.1	23.2	22.6	21.1	20.6
29.5	29.7	29.4	30	29.2	30.3	23.2	22.4	21.5	21
29.6	29.9	28	29.8	29.3	30.2	23.1	22.3	21.5	21
29.6	30.3	27.7	29.8	29	30.3	23	22.2	21.5	21.1
29.7	30.6	27.6	29.9	29.1	30.2	23	22.3	21.6	21.2
29.7	31.1	27.3	30.3	29	30.3	23	22.1	21.6	21.4
29.4	31.5	27.5	29.9	29	30	22.9	22.3	21.5	21.1
29.3	31.9	27.6	30.3	29	30.1	23	22.6	21.4	21
29.5	32.1	26.9	30.6	28.8	30	23.1	22.8	21.8	21.3
29.6	32	26.7	30.5	29.1	30.1	23.1	22.7	21.7	21.4
30	32.4	26.4	30.6	29	30.4	23.2	22.6	21.8	20.9
30.1	32.7	27.2	30.9	28.9	30.8	23.3	22.6	21.9	20.8
29.9	32.8	27.9	30.9	29.2	31	23.3	22.6	22	20.9
29.4	32.8	27.2	30.8	29	30.5	23.4(Maximum Weight)	22.7	22.1	20.9
29.7	32.5	27.5	30.9	29.3	30.8	23.4	22.7	22	20.9
29.6	32.3	27.7	30.7	29.4	31	23	22.6	22	20.9
29.8	32.7	27.3	30.4	29.7	31.1	22.9	22.5	21.9	20.9
30.1	32.8	26.5	31.1	29.6	31	22.6	22.5	22	20.8
30.5	32.8	26.7	31.3	29.5	31.4	22.5	22.3	22.1	20.7
30.2	32.9	27.2	31.5	29.7	31.9	22.5	22.2	21.8	20.8
30.4	32.8	28.3	31.7	30.1	31.8	22.4	22.1	21.8	20.6
30.8	33	28.3	31.7	30.3	32	22.3	22.2	21.9	20.6
30.6	33.1	27.7	31.9	30.4	32	22.1	22.4	21.8	21
30.5	33	27.1	32	29.9	32	22.1	22.4	22	21.2
30.7	33	27.7	32.4	30	32.4	22.1	22.3	22.1	21.5
31.2	33.1	27.1	32.4	29.8	32.1	22.1	22.3	22.2	21.6
31.1	33.2	28.3	32.2	30	32.2	22.1	22.4	22.3	21.6
30.6	33.2	28	32.2	30	32.2	22.2	22.5	22.1	21.4
31.2	33.6	27.6	32.6	30.1	32.4	22.4	22.4	22	21.5
31.2	33.9	28.5	32.7	29.9	32.4	22.4	22.4	22.2	21.5
30.5	33.9	28.9	32.9	30.1	32.4	22.5	22.5	22.3(Maximum Weight)	21
31.4	34	29.3	33.1	30	32.5	22.5	22.5	22	20
31	34.1(Maximum Weight)	29	33.2	30.2	32.8	22.7	22.6	21.9	20.4
31	34	29.1	33.7	30.1	32.3	22.6	22.7	22.2	20.5
30.9	33.5	29.2	33.8	30	32.6	22.7	22.6	22	20.8
31	33.5	29.8(Maximum Weight)	34	30	32.8	22.6	22.6	21.8	20.4
31.1	33.8	27.3	34(Maximum Weight)	30.4	32.9	22.7	22.7	21.8	20.5
31.5	33.8	27.9	33.9	30.7	33.1	22.8	22.8	21.7	20.8
31.7(Maximum Weight)	33.9	27.4	33.9	30.8(Maximum Weight)	32.6	22.9	22.9(Maximum Weight)	21.5	21
31.5	33.5	27.3	33.8	30.4	31.8	22.8	22.8	21.6	21
30.6	33.3	27.8	33.8	30.4	32.1	22.9	22.8	21.2	21
30.8	33.3	27.7	33.5	30.4	32.2	22.9	22.8	21.2	21
31.3	33.4	27.5	33.8	30.2	32.4	22.8	22.8	21.3	21.1
31.4	33.6	27.2	33.6	29.8	32.6	22.7	22.8	21.2	21.3
31.6	33.5	27.3	33.5	30.3	32.8	22.7	22.7	21.3	21.2
30.5	33.6	27.9	33.3	30.3	33.1	22.6	22.8	21.3	21.3
30.2	33.2	27.4	33.1	30.3	33.5	22.7	22.8	21.4	21.3
30.2	33.6	27.7	33	30.2	33.6(Maximum Weight)	22.7	22.7	21.3	21.2
30.9	33.6	27.4	33.2	30.5	33.4	22.5	22.6	20.4	21.5
30.2	33.1	27.2	33	30.4	33.3	22.4	22.6	20.1(10%Reduction)	21.3
29.8	32.9	28.2	33.1	30.5	33.2	22.3	22.9	19.8	21.6
30.5	33	27.4	33	30.5	33	22.4	22.9	19.3	21.7
30.6	32.9	26.7	33	30	32.8	22.3	22.9	19	21.7
29.9	32.7	27.7	33	30	32.4	22.2	22.7	18.8	21.6
29.7	31.9	27.4	32.7	30	32.4	22.2	22.7	18.4	21.8
29.1	31.9	27.4	32.7	29.9	32.4	22.1	22.7	17.4	21.6
29	31.4	27.7	32.1	29.7	32.3	22.2	22.7	17.3	21.7
29.3	31	27.2	32	29.6	31.9	22.2	22.8	16.3(Humane End Point)	21.8
30.1	31	27	32	29.6	31.6	22.1	22.7		21.6
30.4	31	26.9(10%Reduction)	31.9	29.7	32	21.9	22.6		21.5
29.7	31	26.4	31.5	29.7	31.7	21.3	22.5		21.5
30	30.7(10%Reduction)	26.4	31.6	29.6	31.4	21.6	22.7		21.5
29.1	30	26.1	31.5	29.1	31.3	21.7	22.8		21.6
28.9	29.6	26.1	31.4	29.1	30.5(10%Reduction)	21.7	22.8		21.6
29.2	29.2	25.9	31.7	29	30	21.7	22.7		21.9
28.6(10%Reduction)	28.1	25.5	31.6	29.3	29.5	21.6	22.7		21.9
28.7	27	25.9	31.5	29.4	30	21.5	22.6		22.1(Maximum Weight)
27.2	26.1	25.4	31.7	29.6	30	21.4	22.6		22
27.2	25.2	25	31.4	29.5	30.2	21.5	22.7		21.8
27.2	24.6(Humane End Point)	25	31.4	29.7	30	21.3	22.5		21.7
27.3		24.9	31	28.9	30	21.1(Approx10%Reduction)	22.6		21.9
27		24.7	31	28.5	30	21.1	22.6		21.7
27		24.4	31	28.3	29.4	21.1	22.6		21.4
26.5		24.4	30.9	28.2	29.4	21.1	22.5		21.1
26.5		24.6	31.2	28.3	28.2	21.2	22.6		21.4
26.5		24.4	31	27.9	28.1	21.2	22.5		21.5
25.6		24.4	30.4(10%Reduction)	27.9	28.2	21.1	22.4		21
23.9(Humane End Point)		24	30.2	27.8	27.9	20.2	22.8		20.8
		23.7	30.3	27.9	27.8	20.3	22.6		20.4
		23.7	30.1	27.8	27.8	20	22.4		20.3
		23.6	30	27.8	27.4	20	22		20.3
		23.7	29.9	27.8	27.5	19.8	22.1		20.1
		23.6	29.8	27.7	27	19.4	22		20.1
		23.2	29.6	27.6	26.9	19.4	22.8		20.3
		23.4	29.7	27.6	25.8	19.4	22.3		20.2
		23.4	30	28	24.8	19.2	21		20.1
		23.3	29.8	27.9	24.7(Humane End Point)	19	21.3		20.2
		22.9	29.5	27.5(10%Reduction)		19	21.3		20.3
		22.8	29	27.5		19	21.2		20.3
		22.8	28.1	26.6		18.9	21.3		20.3
		22.4	27.2	26.5		18.5	21		20.4
		22(Humane End Point)	26(Humane End Point)	25.8		18.1	21		20.2
				25.7		18	21.1		20
				25.8		17.3	20.4(10%Reduction)		20
				25.7		17.1(Humane End Point)	20.1		20.3
				25.9			20.3		20.3
				26			20.4		19.8(Approx10%Reduction)
				26.4			20.2		19.6
				26.1			20.1		19.3
				25.8			19.8		19.3
				24.9			19.7		19.1
				24.6			19.2		18.8
				23(Humane End Point)			19.1		19
							19		18.7
							18.5		18.4
							17.5(Humane End Point)		18.3
									17.8
									17.5
									16.6(Humane End Point)
